# Tracing metallurgical links and silver provenance in Balkan coinage (5th -1st centuries BCE)

**DOI:** 10.1007/s12520-024-02106-1

**Published:** 2024-11-13

**Authors:** Katrin Julia Westner, Janne Blichert-Toft, Liesel Gentelli, Eftimija Pavlovska, François de Callataÿ, Francis Albarède

**Affiliations:** 1 Ecole Normale Supérieure de Lyon and CNRS, Lyon, France; 2 National Bank of the Republic of North Macedonia, Skopje, North Macedonia; 3 Royal Library of Belgium, Brussels, Belgium and Ecole Pratique des Hautes Etudes, Paris, France

**Keywords:** Coinage, Silver, Pb isotopes, Ag isotopes, Balkans, Mixing

## Abstract

Local types of coinage testify to the emerging use of silver in the Balkan interior, possibly related to abundant ore deposits in the region. Here, we present Pb isotope data for silver coins minted by local tribes and settlements (anepigraphic coins attributed to the Derrones/Laeaei, Damastion, Pelagia, Kings of Paeonia) between the 5th and 3rd centuries BCE. For comparison, we analysed coinage of the potential Greek *emporia* Dyrrhachium and Apollonia for their main element and Pb-Ag isotope compositions. Statistical data evaluation demonstrates close material connections between coins from the Balkan interior and identify a predominantly local raw material provenance. The majority of Damastion’s issues defines a tight cluster fitting Strabo’s (VII.7.8) account that the settlement possessed nearby silver mines in the Balkan interior. Novobërdë/Novo Brdo (Kosovo) can be plausibly hypothesised among the available reference data to have been one of the main ore districts supplying the mint. Mixing is evident for coins from the Kings of Paeonia and coinage attributed to the Derrones/Laeaei. Virtually identical end-members suggest that Paeonian regal coinage recycled tribal issues with contribution of metal obtained from Damastion’s hypothesised mines. Contemporaneous coinage struck by Dyrrhachium as well as end-members calculated for Thasos and the Macedon kingdom (Albarede et al. in Bullion mixtures in silver coinage from ancient Greece and Egypt, J Archaeol Sci 162:105918, 2024a) signal metal sourced from the Balkans, presumably the Macedonian/Paeonian border area. Comparison of data from Greek city-states and coinage issued by Apollonia and Dyrrhachium for the Romans demonstrates a change in the type and origin of raw materials and bullion composition, indicating a shift in monetary customs and possibly metal production technology.

**Supplementary Information:**

The online version contains supplementary material available at 10.1007/s12520-024-02106-1.

## Introduction

While production and use of silver in the Aegean dates back to at least the 4th and 6th millennia BCE, respectively (Bassiakos et al. 2019; Malamidou et al. 2022; Maran 2021), the metal makes a rather late appearance in the interior of the Balkan Peninsula (e.g. Vukmanović and Medović 1990). Local types of silver coins are among the most distinctive evidence for the emerging use of the metal. They were issued in two chronological phases, i.e. between the late 6th and the first half of the 5th century BCE, and between the 4th and 3rd centuries BCE. This period was a changeful time with the Greco-Persian wars, the rise of the Macedon kingdom under Philipp II to the dominating power in Greece, and the creation of an empire by his successor Alexander III the extent of which was unprecedented. Furthermore, the appearance of coinage in the Balkan interior falls in a period of increasing Greek contacts up to the northern parts of North Macedonia and southern Serbia and Kosovo, manifested by imported Greek pottery and metal vessels and the establishment of so-called “Hellenised” settlements characterised by Greek-derived or -inspired architecture and material culture (Baraliu and Muharremi 2019; Pare 1997; Sanev 2013; Vranić 2014, 2022).

**Fig. 1:**
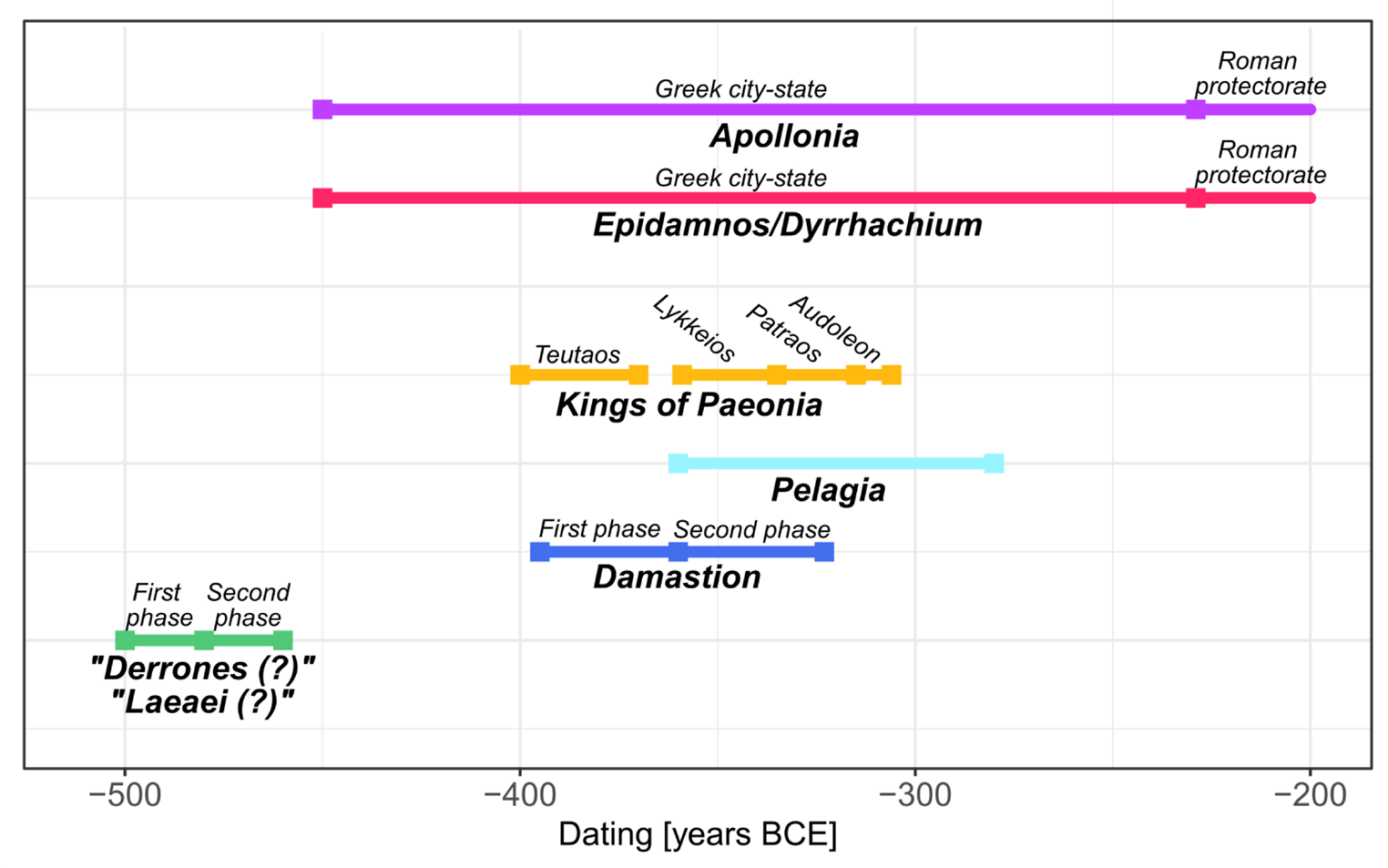
Timeline of coin series issued by the investigated minting authorities. The “Laeaei (?)” coins all belong to the second minting phase dated to between c. 480 − 460 BCE. Note that the timescale is cut at 200 BCE to facilitate the readability of the figure

The earliest coin series in the Balkan interior were minted by local *ethne* inhabiting the territory around the rivers Vardar/Axios, Struma/Strymonas, and Mesta/Nestos in ancient Macedonia, Thrace, and Paeonia (principally modern-day northern Greece, North Macedonia, and western Bulgaria) between the late 6th and the first half of the 5th century BCE (Fig. 1; Paunov 2015). Since these coinages were often minted in large denominations (c. 30–40 g) and were found predominantly in Egypt and the near East, they either have been linked to the Persian military presence in the region (Picard 2000; Tzamalis 2011) or have been alternatively interpreted as large-scale monetary transactions traded to an area lacking substantial silver ore deposits (Dahmen 2010). With Persian control of Greece being significantly diminished after the end of the empire’s failed second invasion in 479 BCE, it is not clear if later coinage series issued in the 470s to 450s BCE could be related to payments made to the Persians (Wartenberg 2015).

The chronology and weight standards as well as the exact localisations of the tribes and the organisation of minting continue to be much discussed (Psoma 2006, 2015; Tzamalis 2012a; Wartenberg 2015). Due to many similarities among their issues, a general consensus exists which considers dodecadrachms, decadrachms, octodrachms, staters, and drachms of the Bisaltai, Derrones, Ichnai, Orrescii, Getas, and the herdsman/incuse and centaur/maenad issues as part of a broader Pangaeon numismatic district (de Callataÿ et al. 2023), implying that the metal for these coinages was sourced from the Pangaeon mountains and other deposits in northern Greece (Tzamalis 2012a). The end of tribal coinages in the late 460s BCE consequently has been linked to expansion of the Macedon kingdom under Alexander I who gained control over prolific mines in the Dysoron and Pangaeon mountains previously exploited by local *ethne* (Paunov 2015). The attribution of mostly anepigraphic smaller-scaled denominations to specific tribal groups is more ambiguous as different obverse and reverse imagery is frequently combined with a variety of symbols and legends. Several hypotheses on their classification have therefore been put forward (Gaebler 1906; Svoronos 1919; Tzamalis 2012b).

In the 4th and 3rd centuries BCE, coins were struck in the Balkan interior by the following minting authorities:

- Damastion, a city reportedly founded by Greek settlers near silver mines (Strabo, VII.7.8 and VIII.6.16; Aly 1931), presumably to export the locally extracted metal to Aegean Greece in the form of coinage (May 1939);
- In the name of indigenous groups, rulers, or settlements (e.g. Pelagia) that mostly used similar types as Damastion; and.
- The Kings of Paeonia (Gaebler 1927; Pavlovska 2008).

The resurgence of coins issued in the name of indigenous entities in the Balkan interior appears to be tied to Damastion’s minting activity. Due to the alignment of the weight standard and style of Damastion’s later issues with those of the Kings of Paeonia, Damastion has been placed in the neighbourhood of Paeonia (May 1939), which in turn is located north of ancient Macedonia and mainly comprises the territory around the northern lower and middle parts of the Vardar/Axios river until the Struma/Strymonas river in the east (Theodossiev 2000). With the defeat of the Paeonian kingdom by Philip II in 359/8 BCE (Diodorus Siculus, 16.4.2), it is hypothesised that the mines of Damastion came under the control of the Macedon kingdom (Hammond 1994; Strauss 1984).

The interpretation of the coinages issued by local *ethne* and Damastion, which had the widest circulation of these coin types, share some similarities. The coins have been viewed in the framework of abundant mineral wealth and the metal demand of external powers which triggered the production of export-driven coinage in regions whose indigenous inhabitants had little to no primal use or need for it. This model, however, does not incorporate the existence of widespread small denominations, overstrikes contradicting consistent minting of freshly extracted metal, and evidence for domestic use of currency, i.e. monetisation in the true sense of the word (Wartenberg 2015). It also does not consider the coinages in their local cultural and historical context (e.g. the connection and reciprocal influences between Damastion and the Kings of Paeonia).

Lead isotope provenance studies have a high potential to contribute to the understanding of these coinages and their production and use by.

- Reconstructing their raw material sources with respect to the chronology and/or typology of the issues and by helping to identify “fresh” bullion with respect to remelting and recycling,
- Narrowing down possible locations of minting centres,
- Identifying potential material connections between the issuing authorities, and
- Assessing their role in the circum-Mediterranean silver cycle.

For this study, 84 silver coins were sampled, issued from the 5th to 3rd centuries BCE by local *ethne* and Greek-founded city-states in the Balkan interior:

- Anepigraphic and attributed to the tribe of the Derrones (*n* = 16);
- Anepigraphic and attributed to the tribe of the Laeaei (*n* = 1);
- Damastion (*n* = 17);
- Pelagia (*n* = 3);
- Kings of Paeonia (*n* = 47).

This set of coins represents different denominations and was partially derived from known find locations (i.e. single finds, hoards, archaeological excavations) in North Macedonia and Kosovo. In addition, 17 coins were investigated from Apollonia (*n* = 7) and Dyrrhachium (*n* = 10), two Greek-founded cities in modern-day Albania that were advantageously located for trade with the inland as well as seaborne exchanges and became Roman protectorates in 229 BCE. The aim of the study is to test the assumption that Apollonia and Dyrrhachium accessed silver mined from deposits in the interior (Crawford 1985; Hammond 1994; May 1939) and to detect possible changes in the metal sourced for their coinage after the cities became associated with the Roman republic. Detailed information on the investigated minting authorities as grouped by chronological and numismatic aspects is provided in the following section.

## The investigated minting authorities

### Anepigraphic coins attributed to the Derrones and Laeaei

**Fig. 2:**
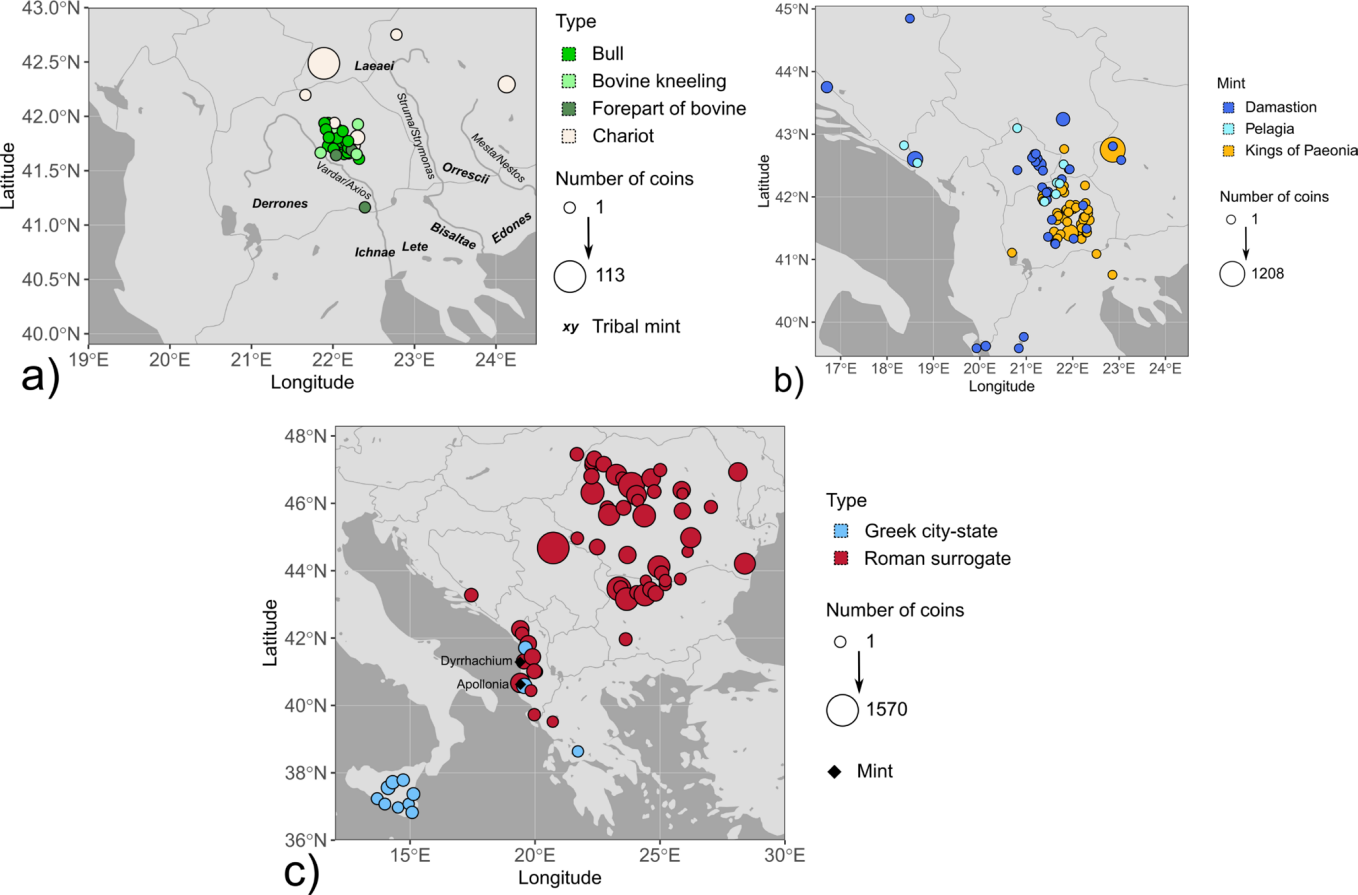
Maps with coin finds of the investigated mint groups. The size of the symbols is related to the number of coins per find and their fill colour reflects different coin types or minting authorities. The mint locations are shown on the maps if known. The data points are slightly jittered to reduce overlap. Find spots were compiled from Alaj (2019), Jakimovski and Ilievski (2022), Josifovski (2006), May (1939), Pavlovska (2012), Pavlovska (2017), Popović (2007), Popović and Vranić (2013), Prokopov (2007), Thompson et al. (1973), Tzamalis (2012a), Ujes (2002) and Wright (2012). (**a**) Single and hoard finds of coins found in the Balkan interior showing a bull, bovine kneeling, forepart of a bovine, and a chariot, which are dated to the first half of the 5th century BCE and attributed to the tribes of the Derrones and Laeaei. Locations of tribal mints were taken from http://nomisma.org/ (accessed 18 February 2024). (**b**) Single and hoard finds of coins from Damastion, Pelagia, and the Kings of Paeonia issued in the 4th and 3rd centuries BCE. (**c**) Hoard finds of coins from Apollonia and Dyrrachium as Greek city-states and Roman protectorates (after 229 BCE), which were struck between the 5th and 1st centuries BCE. Note that coins from Apollonia and Dyrrhachium are not distinguished in this map due to their comparable distribution

Anepigraphic coins in small denominations with different obverse imagery (forepart of bovine, bovine kneeling, and bull) are among the earliest coins from the Balkan Peninsula and were issued in the first half of the 5th century BCE (Fig. 1). They were found in eastern-central North Macedonia near the modern-day settlements of Veles, Shtip, Sveti Nikole, and Radovish (Fig. 2a; Josifovski 2006; Pavlovska 2008). The coin types have been proposed to be attributed to the Derrones, as their iconography is similar to dodecadrachms and decadrachms linked to this tribe, which show an ox-driven chariot on the obverse combined with different reverses and additional symbols (Josifovski 2006). Due to their small denomination values, it has been hypothesised that these issues were intended for domestic use and that their find distribution therefore indicates the territory inhabited by the Derrones (Josifovski 2006; Pavlovska 2008). Based on the link by legends (ORR and ΛΙΤΑ series) and symbol (flower; one series), Tzamalis (2012b) proposes that all coins of the kneeling bull series might be considered as fractions of the triple staters depicting a herdsman with two oxen. The herdsman issues are either anepigraphic or possess inscriptions presumably referring to Getas, the Ichnae, Tyntenoi, or Orrescii (Tzamalis 2012a).

Most of the investigated coins belong to the second minting phase of the Derrones dodecadrachms and decadrachms (c. 480 − 460 BCE). Only one coin, which was previously attributed to the Orrescii (Svoronos 1919, pp 57, 13), a tribe placed in the broader Thasian Peraia between the rivers Struma/Strymonas and Mesta/Nestos (Psoma 2015), is contemporaneous with their earliest minting phase (c. 500 − 480 BCE; Fig. 1). Coin hoards from the second minting phase of the Derrones were predominantly discovered in the interior of the Balkans – as opposed to coins from the first phase being known mostly from hoards in Egypt – and were linked to Persian military payments (Tzamalis 2011, 2013); their find spots overlap with those of the small denominations (Fig. 2a). An octodrachm contemporaneous with the latter minting phase of the Derrones belongs to a series which, based on letters identified on these coin types, is commonly assigned to the tribe of the Laeaei (first by Svoronos 1919) mentioned by Thucydides (II.96.3) to live at the upper course of the river Struma/Strymonas. Tzamalis (2012a), in contrast, groups these coins together with issues of the Derrones, with whose coinage it is iconographically closely related (Pavlovska 2008). As the attribution of the anepigraphic coin series investigated in this study is not concludingly resolved, their minting authorities will in the following text be labelled as “Derrones (?)” and “Laeaei (?)”, respectively.

### Damastion and Pelagia

Damastion was a settlement reportedly founded by Greek settlers from Aegina and Mende (Strabo, VIII.6.16; Aly 1931) near silver mines (Strabo, VII.7.8) at an unknown location in the interior of the Balkan Peninsula and issued silver coinage in the 4th century BCE (Fig. 1). Due to the wide distribution (Fig. 2b) and large volume of Damastion’s coinage (de Callataÿ et al. 2021), which has been deemed too extensive for serving purely domestic purposes, it was assumed that its main function was to export silver extracted from its mines in the form of bullion, accessed by, for example, Corinth, Corcyra, or their colonies on the Adriatic coast (May 1939). Aegean Greek influences, specifically similarities to the coinage of the Chalcidian League, were seen as predominant in the iconography and style of coins from Damastion’s earlier minting period (c. 395 − 360 BCE). In the later phase (c. 360 − 323 BCE), its issues were orientated closer towards the coinage of the Kings of Paeonia, manifested by alignment of the weight standard and sharing of dies, as well as a change in style, with the imagery and craftsmanship of many coins being comparable to Paeonian issues. Damastion therefore was placed in the neighbourhood of the kingdom of Paeonia (Gaebler 1927; May 1939). Based on the concentration of coin finds (Fig. 1b) and the presence of silver-bearing ore deposits (Janković 1997), Damastion was proposed to have been situated north of Paeonia, in the area of modern-day southern Serbia and Kosovo (Petrova 1991; Ujes 2002). The closure of the mint at the end of the 4th century BCE has been linked to the territorial expansion of neighbouring Dardanian tribes from the north (Petrova 1991), possibly in the course of the Celtic invasions.

Several lesser coin series based on the issues of Damastion are known from the Balkan interior, of which the coinage issued in the name of the settlement Pelagia is the largest (Figs. 1 and 2b). Pelagia is assumed to have been located close to the northwestern part of Paeonia and not far south from the border of Dardania (May 1939), which corresponds to modern-day Kosovo and parts of Serbia and North Macedonia, with Sokolovska (1990) placing it near modern-day Tetovo in northwestern North Macedonia. While the earlier issues of Pelagia presumably were struck at Damastion (until closure of the mint), subsequent coins are believed to have been produced locally and were later based on types of Alexander III and Lysimachus instead of Damastion’s iconography, indicating that Pelagia adopted the imagery of well-known coin types to increase the acceptance of its coinage (May 1939) in the area it circulated. In this sense, Pelagia’s issues are similar to Celtic silver coins whose imagery derived from the Kings of Paeonia, the Macedon kingdom in the name of Philipp II and Alexander III, and Thasos, as the most frequently circulating types in southeastern Europe (Hiriart et al. 2020). Pelagia’s coinage ceased with the Celtic migrations.

### Kings of Paeonia

A diobol from Teutaos (c. 400 − 380/70 BCE) which depicts a standing bull on the obverse, the same iconography as an imagery type of the anepigraphic “Derrones (?)” coins, is thought to be the earliest regal Paeonian coinage (Fig. 1; Pavlovska 2008). Subsequently, three dynasts of Paeonia issued silver coinage in their name, Lykkeios (c. 359/8-340/35 BCE; *n* = 40, most of them from the Vozarci-Shivec hoard; Pavlovska 2008), Patraos (c. 340/35–315 BCE; *n* = 3), and Audoleon (c. 315 − 285/4 BCE; *n* = 4). The sharing of weight standard, dies, and iconographic traits document a close connection with Damastion’s coinage. The distribution of Paeonian coins (Fig. 2b) overlaps with the occurrences of small denominations of the “Derrones (?)” (Fig. 2a) and largely covers the core territory of the kingdom, suggesting a primarily local use.

The Paeonians were defeated by Philipp II in 359/8 BCE and forced to pledge loyalty to the Macedon kingdom (Diodorus Siculus, 16.4.2). Paeonian autonomous silver coinage, however, is taken as an indication that Paeonia was not annexed but rather a vassal state required to pay tribute and supply soldiers for the army of the Macedon kingdom. Paeonia probably became fully independent under Patraos’ rule between 331 BCE and the death of Alexander III in 323 BCE, when it disappears from the records of military service and is no longer listed as a subject of the Macedon kingdom (Merker 1965). Frequent depiction of a fallen enemy wearing a shield of Macedonian type being speared by a horseman on several of Patraos’ tetradrachm issues might refer to Paeonia’s newly gained independency (Pavlovska 2008), but in any case, appears to reflect an incessantly antipathetic stance towards the Macedon kingdom (Wright 2012). Audoleon, the successor of Patraos, struck standard Alexander-type coins of which some series displayed the king’s name in the style of the diadochs (Waggoner 1983), emphasising Paeonia’s claim as a sovereign kingdom. The silver coinage of the Kings of Paeonia ceases after Audoleon’s reign with the Celtic migrations, and later currency was only made in bronze.

### Apollonia and Dyrrhachium

In contrast to the other minting authorities investigated in this study, Apollonia (Illyria) and Dyrrhachium (founded under the name of Epidamnos) are not situated in the Balkan interior but along the Adriatic coast in modern-day Albania. Their advantageous location at endpoints of the later Via Egnatia enabled comparatively easy access to the inland as well as Italy and mainland Greece by a short sea-crossing, suggesting that they might have functioned as *emporia*. Epidamnos and Apollonia were founded by Corcyrians with the participation of Corinthians in the middle of the 7th century BCE and became Roman protectorates in the course of the Illyrian Wars (229 − 228 BCE; Antonetti and Matijašić, 2013). Apollonia and Dyrrhachium started minting coins in the 5th century BCE and issued large-scale coinages for the Romans (“surrogate coinage”; cf. de Callataÿ 2016). Based on the composition of coin hoards, the output of Dyrrhachium’s mint was approximately four times larger than that of Apollonia (de Callataÿ 2016). The coinages of Apollonia and Dyrrhachium widely circulated in southeastern Europe between 120 − 60/55 BCE (Meta 2012; Picard and Gjongecaj 2000) and were discovered in numerous hoard finds that were deposited particularly in Romania and Bulgaria by Thracian auxiliaries returning home after serving in Rome’s military (de Callataÿ 2023). This is in stark contrast to the distribution of earlier coin finds from Apollonia and Dyrrhachium as autonomous Greek city-states, which concentrate in Sicily and are mostly found together with local issues or coins from Corinth and its other colonies (Fig. 2c). Coinage from Dyrrhachium was used for overstrikes by Greek colonies in southern Italy and Sicily (MacDonald 2009), partially serving as material basis for their issues. The coinage of Apollonia and Dyrrhachium ceases in the 1st century BCE. Due to a lack of silver deposits in the broader vicinity of Apollonia and Dyrrhachium, it is hypothesised that the cities possibly obtained bullion from Damastion when this mint was still active (Crawford 1985). For the later surrogate coinage, silver circulating in Greece prior to the Romans, collected by the Romans from sanctuaries and private persons, or metal sourced from specialised private moneylenders (Roman or not) have been proposed as possible raw material sources (de Callataÿ 2016).

## Materials and methods

### Sampling and sample preparation

A total of 101 coins was investigated. They derive from four different collections and from professional dealers (Table S1):

- The Museum of the National Bank of the Republic of North Macedonia (NBRM) in Skopje, North Macedonia (*n* = 73);
- The Archaeological Museum in Münster, Germany (*n* = 9);
- The Royal Library in Brussels, Belgium (*n* = 8);
- The Archaeological Institute of Kosovo (AIK; *n* = 1) and Museum of Kosovo (*n* = 1) in Prishtinë/Priština, Kosovo; and.
- Purchased from certified professional dealers (*n* = 9).

Coins from the Archaeological Museum in Münster and purchased specimens were sampled by drilling their edges. Coinage from the NBRM and the Royal Library of Brussels was sampled by etching, a method that extracts minute quantities of material by rolling the coin edges on chromatographic paper strips loaded with an etching solution consisting of a 1:1:1 mixture of H_2_O_2_, NH_4_OH, and H_2_O with SiC powder added to increase abrasion efficiency (Gentelli et al. 2021; Milot et al. 2021a). The drilled metal samples and chromatographic paper strips were processed for chemical and isotopic analyses according to the procedures detailed in Milot et al. (2021b) and Gentelli et al. (2021), respectively. Lead separation of sample solutions derived from drilled metal samples and leached chromatographic paper strips was carried out in the clean laboratory of the Ecole Normale Supérieure (ENS) de Lyon following the procedures outlined in Milot et al. (2021b).

Coins from the AIK and Kosovo Museum were sampled during a previous project of the first author (Gassmann et al. 2022; Westner et al. 2017) by gently abrading their edges with roughened quartz glass rods, which were subsequently stored in centrifuge tubes until transferring them into the clean laboratory. Metal adhering to the quartz glass rods was recovered by filling the storage tubes with 6 M HNO_3_ and letting them sit for several days to ensure complete dissolution. The sample solution was subsequently transferred into Teflon beakers, evaporated, taken up with 1 M HBr, and used for chromatographic Pb separation in the clean laboratory of the Deutsches Bergbau-Museum Bochum (German Mining Museum Bochum; DBM).

While Pb purification was carried out for all samples regardless of the sampling method used (drilling or etching), Ag separation was performed only on material obtained by drilling, which allows accessing the core metal of the coins unaffected by surface processes such as post-depositional corrosion and conservational treatment. Extensive analytical work by our group has shown that Pb isotopes are not affected by the same surface processes as Ag isotopes (Milot et al. 2021a). Sample solutions of drilled specimens were analysed for their Ag, Cu, and Pb concentrations nd their Pb and Ag isotope compositions. Lead and Ag were separated for isotope analysis according to the protocols detailed in Milot et al. (2021a). High contents of matrix elements, mainly Cu, can be difficult to separate from Ag and sample P347 had to be excluded from Ag isotope analysis for this reason.

### Analytical techniques

The Ag, Pb, and Cu element abundances of the samples were determined with a quadrupole ICP-MS (iCAP-Q), and their Pb and Ag isotope compositions were measured with a Nu Plasma 500 HR MC-ICP-MS, both housed at the ENS de Lyon. The compositional data are provided as ratios of Cu and Pb scaled to Ag since the amount of the drill samples was too small for weighing and therefore no absolute elemental quantities could be calculated. Detailed descriptions of the analytical protocols used at ENS de Lyon are available in Westner et al. (2023). The Pb isotope composition of the coins from the AIK and Kosovo Museum were determined at the CEZA Mannheim using a NeptunePlus MC-ICP-MS.

### Statistical evaluation of data

The mint groups as described in Sect. 2 were first checked by a mixing model based on principal component analysis (PCA) for the prevalence of recycled metal in their coin Pb isotope compositions. Quasi-binary mixing between two ore source end-members is assumed if the first component c_1_ makes up for the majority of the data set’s total variation, typically 97–99% (Albarede et al. 2024a). If mixing was not corroborated based on the proportion of the first component, cluster analysis was carried out to establish distinct data subsets related to raw material source groups. For the cluster analysis, natural Pb isotope abundances were re-calculated from the Pb isotope ratios (Tomczyk and Żabiński 2023) to remove correlations particularly affecting ^204^Pb-based ratios (Albarede et al. 2020). The data were subsequently centred and squared and the ideal number of clusters was determined based on the Hubert and D indexes, yielding two to three clusters per group. Clusters were then calculated by k-means clustering. For the drilled samples (i.e. all coins from Apollonia and Dyrrhachium), data evaluation was carried out based on their complete data sets which also comprise Ag isotopic and Ag-Cu-Pb compositional data.

Possible metal sources of the end-members and coins from the mint group clusters, respectively, were assessed by an algorithm that calculates distances between an individual Pb isotope datum and ore reference data (Albarede et al. 2024b). A major difference of this approach to other statistical evaluation methods applied in Pb isotope provenance studies (e.g. Euclidean distances) is that it takes mass-dependent isotopic fractionation ubiquitously present in ore data into account. Ore reference data along the mixing line between end-members were considered in the data evaluation to assess the potential additional presence of geologically similar raw material sources in the binary mixing model. The Pb isotope reference database used in this study is an updated version of the dataset compiled by Blichert-Toft et al. (2016), currently comprising c. 7000 entries of Pb-Ag ores from Europe, northern Africa, and the near and middle East. We applied the following strategy for assessing the calculation results:

1. Although reference data with a distance within the 95% confidence level (chi-squared value ≤ 5.99) have a sufficient proximity to the artefact data to be considered as permissible sources (termed “hits” for short), the threshold value was reduced to 3 to eliminate most random hit results.
2. Only mining districts with multiple hits (> 3) were accepted as prospective source regions to avoid coincidental findings.
3. Areas with little or no historical-archaeological evidence for noticeable mining activity and/or for trade connection with the study area, i.e. Tunisia but also Germany, France and the United Kingdom, are currently regarded as unsupported metal sources and were not included in the provenance assessment.
4. Hits not positively fulfilling the three previous conditions likely are caused by the presence of Pb mixtures of different origin in the coinage bullion.

## Results

**Fig. 3:**
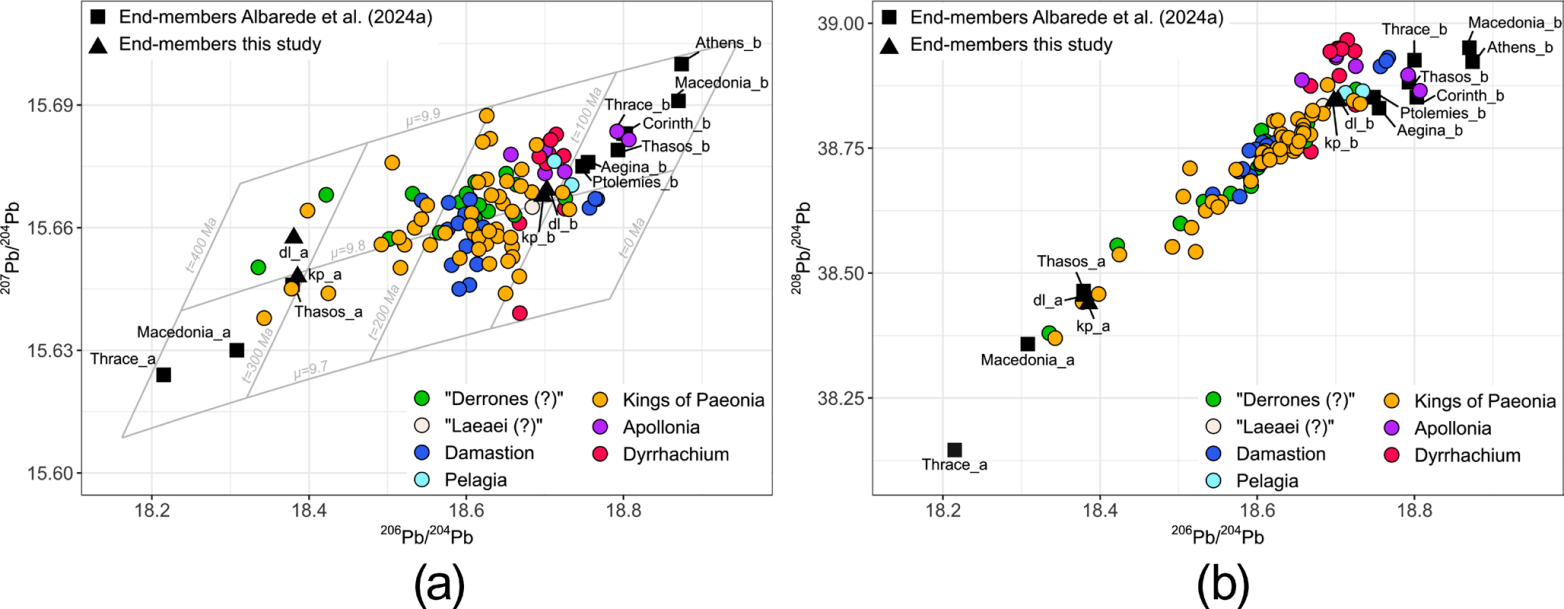
Diagrams of ^204^Pb-based Pb isotope ratios of the investigated coins. The symbol fill colour relates to the respective minting authorities. Mixing end-members were calculated according to Albarede et al. (2024a) on the basis of the Pb isotope data for the mint groups, which in turn are based on chronological and numismatic aspects as described in Sect. 2 (dl=”Derrones (?)” and “Laeaei (?)”; kp = Kings of Paeonia). Mixing was not found to be supported for the Apollonia and Dyrrhachium mint group (see text for further details). Mixing end-members calculated for ancient Greek coinage (Albarede et al. 2024a) are shown for comparison; note that some of these geologically old end-member values plot outside the range of the diagrams. Lead isotope model parameters were calculated according to Albarede and Juteau (1984)

Fig. 3 details conventional diagrams of Pb isotope data with mixing end-members calculated for the mint groups. Significant overlap exists between data from the Balkan interior coins and coinage from Dyrrhachium issued before the city became a Roman protectorate.

### ”Derrones (?)” and “Laeaei (?)”

**Fig. 4:**
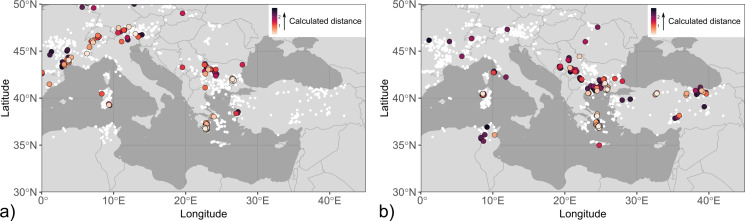
Maps of Pb isotope hits determined by a distance-based algorithm including correction for mass-dependent fractionation (Albarede et al. 2024b) for mixing end-members calculated by PCA (Albarede et al. 2024a) for anepigraphic coins attributed to the tribal groups of the Derrones and Laeaei. The fill colour of the symbols refers to the value of the distance *d* between end-members and ore reference data. The localities of ore data from the Lyon database are shown as white circles. (**a**) Low-^206^Pb/^204^Pb end-member, (**b**) high-^206^Pb/^204^Pb end-member

Mixing is evident for the “Derrones (?)” and “Laeaei (?)” coins, with the first component accounting for 99% of the total variation (Figure S2). Permissible sources for the low-^206^Pb/^204^Pb end-member (Fig. 4; Table S1) comprise the Sakar Mountains in southeast Bulgaria (Elchovo district), the Parnon Mountains in the Peloponnese (Tyros and Molaoi districts), and the Bulgarian Panagyurishte district of the Apuseni-Banat-Timok-Srednogorie (ABTS) belt (von Quadt et al. 2005). The ABTS, however, is particularly in its Bulgarian zone dominated by Cu-Au mineralisation. The low-^206^Pb/^204^Pb end-member overall is not well constrained. Most hits from the database occur in areas of Europe with pronounced remnants of the Variscan orogenies particularly in France, Great Britain, and Germany, which are considered historically unsupported for the investigated coins, but might also be proxies for hitherto uncharacterised local mineralisations with similar Pb isotope ratios. Multiple hits for the high-^206^Pb/^204^Pb end-member are concentrated predominantly in the southern part of the Serbomacedonian-Rhodope belt (SMRB; Heinrich and Neubauer 2002), including deposits in the eastern Rhodopes (e.g. Zvezdel, Kirki districts), the Pangaeon, Palaea Kavala and Zletovo districts and other deposits in the Balkan interior. The location of Zletovo is within the find spot distribution of the coins. Further permissible sources include deposits in Siphnos, the eastern Pontides/Turkey and northwestern Sardinia (Castello di Bonvei). The hits determined at Kythnos and Sardinia predominantly are from Cu-dominant mineralisation.

The earliest issue of the “Derrones (?)” coins (sample SK064) dated to c. 500 − 480 BCE (Pavlovska 2008) is distinguished from the remaining coins (dated c. 480 − 460 BCE) by its higher ^206^Pb/^204^Pb ratios and ranges outside the calculated mixing end-members (Figures S2, S6). The hypothesis of Delev (2014) who links the Derrones with the Agrianes and suggests that the tribe migrated northwards to the upper Struma/Strymonas area between the two emissions (Figs. 1 and 2a) might provide a possible explanation for the observed variation in Pb isotope signatures. The similarity of the Pb isotope ratios of the “Laeaei (?)” coin to those of the contemporaneous anepigraphic “Derrones (?)” issues corroborates the hypothesised close relation of the minting authorities, which appears to have also extended to the raw material supply.

### Damastion and Pelagia

For the complete data set of the Damastion and Pelagonia coins, a first component score of 98% was determined (Figure S2). However, there is a significant gap between coins with a large proportion of the high-^206^Pb/^204^Pb end-member dp_b (> 70%), which all date to the second half of the 4th century BCE, and the main share of the data set (0–40% of the end-member dp_b), whose dating spans across the 4th century BCE (Figures S2, S5). Furthermore, the regression line fit is significantly better for coins closer to the high-^206^Pb/^204^Pb end-member (Figure S3). Cluster analysis of the Pb isotope data confirms the presence of two clusters (dp1, dp2) according to the observed mixing gap (Table S1). The high first component score of the bulk data set presumably results from consistency of µ and κ model parameters at variable model ages (Table S1), which minimises randomised variation of components c_2_ and c_3_ and maximises spread along the c_1_ axis. It therefore gives the impression of mixing but in fact is related to contribution of geologically similar ore sources to the bullion.

Cluster dp1 contains the majority of the data set, including coins from the two minting phases of Damastion and coinage from Pelagia. The extent of isotopic variation and continuous spread of data specifically along the ^206^Pb/^204^Pb axis (Figure S6c, d) documents contribution of isotopically different ore Pb sources to the bullion. Application of the mixing algorithm to the data from cluster dp1 determines a first component score of 90% supportive of mixing (Figure S2) and a high-^206^Pb/^204^Pb end-member whose values (^206^Pb/^204^Pb=18.6199; ^207^Pb/^204^Pb=15.6668; ^208^Pb/^204^Pb=38.765) are within the isotopic range of coins with the maximum ^206^Pb/^204^Pb values in dp1. However, since the lowest proportion of the high-^206^Pb/^204^Pb end-member does not fall below the high level of c. 75% (Figure S5), the low-^206^Pb/^204^Pb end-member is regarded as insufficiently constrained on basis of the available data.

**Fig. 5:**
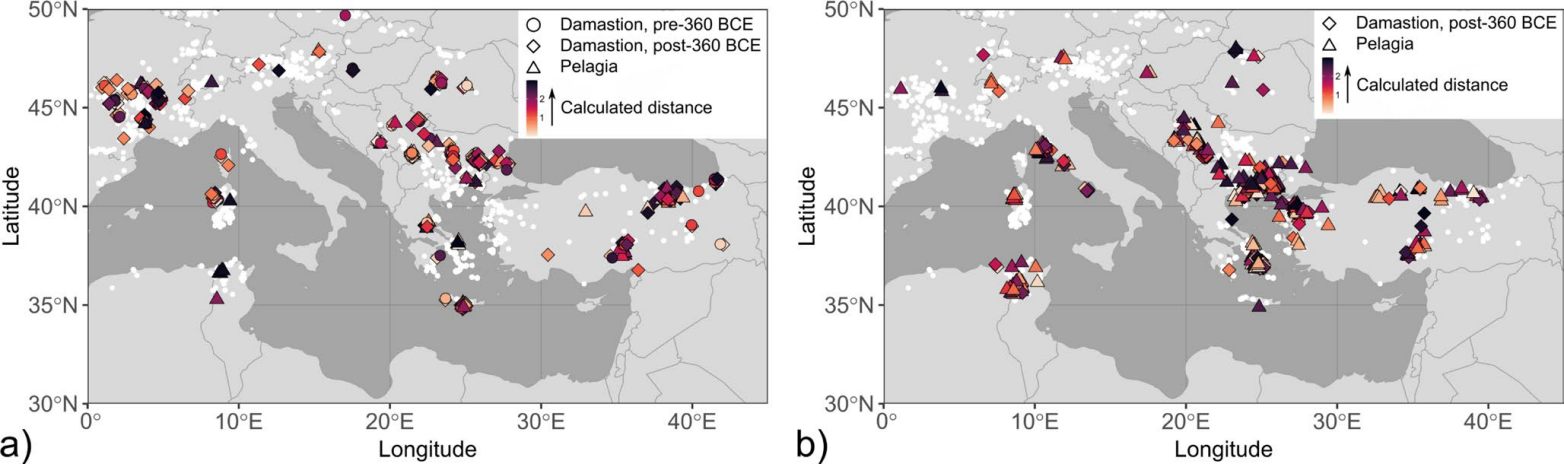
Map of Pb isotope hits calculated with a distance-based algorithm with correction for mass-dependent fractionation (Albarede et al. 2024b) for the clusters of coinage from Damastion and Pelagia and shown as coloured open symbols. The symbol shape reflects different minting authorities and chronological groups as mentioned in the text. The colour of the symbols is according to their calculated distance. The localities of ore data from the Lyon database are shown as white circles. (**a**) dp1, (**b**) dp2

Hits for the high-^206^Pb/^204^Pb coins (and analogously the potential high-^206^Pb/^204^Pb end-member) concentrate at the Novobërdë/Novo Brdo district in Kosovo within the SMRB and the Romanian Apuseni district (including the Roșia Montană deposit) within the ABTS (Fig. 5). These coins constitute the majority of dp1 (nine of 15 coins) and include issues from Damastion’s first minting phase prior to c. 360 BCE. Among the determined permissible sources, the district of Novobërdë/Novo Brdo is the most plausible option for the high-^206^Pb/^204^Pb issues as well as the potential mixing end-member. Novobërdë/Novo Brdo is situated in the neighbourhood of Paeonia, within an area where coin finds from Damastion are concentrated, has a location broadly fitting the description of Strabo (cf. Chapter 2), and the Ag isotope ratios of most of its analysed ores are within the typical coin range (± 1 ε^109^Ag; Westner et al. 2023). Finds of Pb-Ag-rich metallurgical debris produced from local ore (Gassmann et al. 2022; Westner et al. 2023) from a fortified Hellenistic settlement (Gumnishtë/Zlatno gumno) at the Novobërdë/Novo Brdo village (Alaj 2019; Čerškov 1969) support the possibility of local silver production in the 4th century BCE. The permissible sources for the low-^206^Pb/^204^Pb issues, which possibly are isotopic mixtures between the two end-members, include the Parnon Mountains, Sakar Mountains, districts in the Bulgarian and Serbian parts of the ABTS, Menderes Massif and eastern Pontides. The fact that for one of these coins (SK051) no permissible sources agreeing with the criteria outlined in Chap. 3.3 could be determined further argues for Pb of mixed origin being present in the low-^206^Pb/^204^Pb issues.

Coinage from Damastion’s second minting phase and Pelagia in cluster dp2 has higher ^206^Pb/^204^Pb isotope ratios than the coins in dp1. The permissible sources of dp2 (Fig. 5) are centred around the Aegean and include deposits particularly in the southern part of the SMRB (i.e. northeast Chalkidiki, Pangaeon, Palaea Kavala, eastern Rhodopes), the Cyclades, and the Biga Peninsula, Taurus Mountains and eastern Pontides in Turkey.

While the Pb isotope ratios of the few Pelagian specimens analysed do not overlap with those of Damastion’s coins, their general isotopic similarity with issues from the “Derrones (?)” and the Kings of Paeonia firmly connects Pelagia with other coinage from the Balkan interior. The frequent occurrence of plated coins, particularly among Pelagia’s later issues (May 1939), indicates a much more irregular access to silver (also in the form of for example coined silver that could be re-used) than observed for the other minting authorities, which could be linked to the comparatively remote location of Pelagia (cf. Chapter 2).

### Kings of Paeonia

**Fig. 6:**
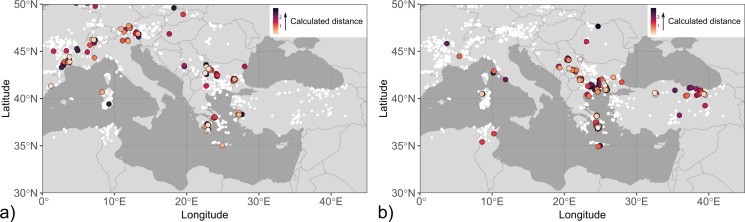
Map of Pb isotope hits determined for mixing end-members calculated for coinage from the Kings of Paeonia. See the caption of Fig. 4 for further details. (**a**) Low-^206^Pb/^204^Pb end-member, (**b**) high-^206^Pb/^204^Pb end-member

Mixing was confirmed for coinage from the Kings of Paeonia based on a first component score of 98%. Three of the coins with the geologically youngest (and one with the geologically oldest) Pb isotope ratios are outside the c_1_ axis range of the mixing end-members (Figure S2). Stronger noise of coin data at low values of c_1_ might indicate variable proportions of components with low-^206^Pb/^204^Pb lead other than the calculated end-member, raising the possibility of multiple geologically old ore sources contributing to the bullion. The similarity of the calculated end-members with those of the “Derrones (?)” and “Laeaei (?)” coins reflects the large overlap of data from the two mint groups. Consequently, permissible ore sources for the end-members are largely identical (Fig. 6). Exceptions are the identification of Menderes Massif in Turkey and Mount Hymettus near Athens (low-^206^Pb/^204^Pb end-member) and South Euboea (high-^206^Pb/^204^Pb end-member) as additional permissible sources while Pangaeon does not fulfil the criteria for prospective source regions. The Pb isotope signatures of most coins issued under the kings Audoleon and Patraos in the second half of the 4th century BCE are dominated by geologically young Pb (Figures S5, S6).

### Apollonia and Dyrrhachium

**Fig. 7:**
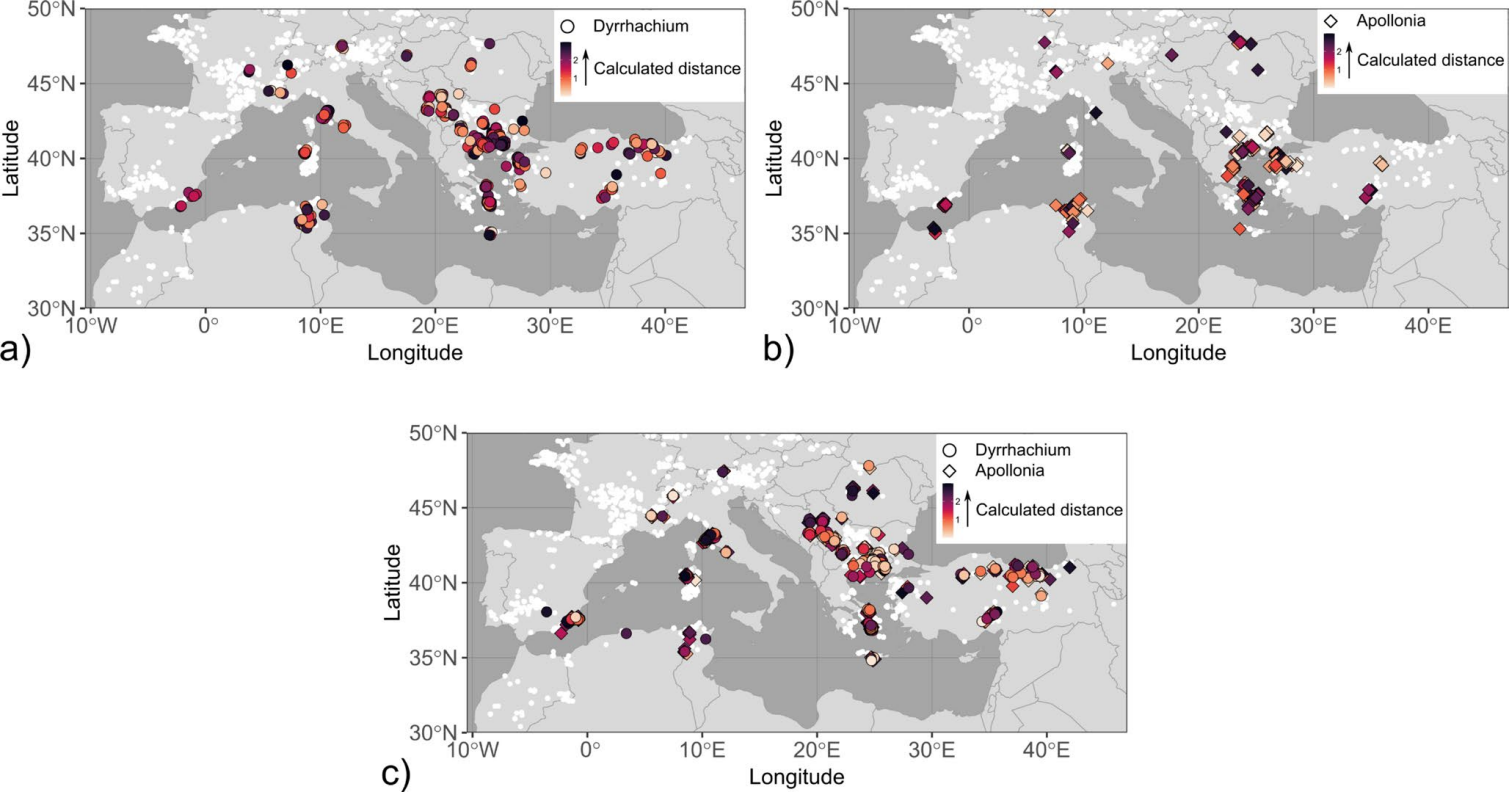
Map of Pb isotopic hits calculated for the clusters of Apollonia and Dyrrhachium. Symbol shapes represent the two minting authorities. See the caption of Fig. 5 for further information. Note that the longitude range has been enlarged to include hits in southeast Spain. (**a**) ad1, (**b**) ad2, (**c**) ad3

Based on a first component value of only 68%, mixing is unsupported for the coins from Apollonia and Dyrrhachium (Figure S2). Cluster analysis separates coins from Dyrrhachium dating to before 120 BCE (ad1) from the later series (ad2, ad3) that are also set apart from the rest of the data set by their Pb isotope ratios (Table S1). Hits for cluster ad1 (Fig. 7) overlap with hits for cluster dp2 and the high-^206^Pb/^204^Pb end-members of the “Derrones (?)” and “Laeaei (?)” and Kings of Paeonia data sets, and are dominated by mineralisation in the SMRB (e.g. Palaea Kavala, Zletovo, eastern Rhodopes) and Sifnos. Among the later coin series, the Pb isotope ratios for two samples from Apollonia in cluster ad2 are markedly different from those of contemporaneous issues minted by Apollonia and Dyrrhachium and similar to high-^206^Pb/^204^Pb mixing end-members calculated for Pb isotope data of coinage from Thasos, Thrace, and Corinth (Fig. 3; Albarede et al. 2024a). Likewise, the hits for ad2 are concentrated in Chalkidiki, Thasos, the Cyclades, and the Biga Peninsula. Permissible sources for cluster ad3 are dominated by deposits in the central (e.g. Madan, Laki districts) and eastern Rhodope Mountains while some hits also occur in other parts of the SMRB (Zletovo, northern Kopaonik) as well as the eastern Pontides, Colline Metallifere (Italy) and the Sierra Cartagena in southeast Spain. Only one sample (P343) has scattered hits with comparatively high distances being indicative of mixing.

**Fig. 8:**
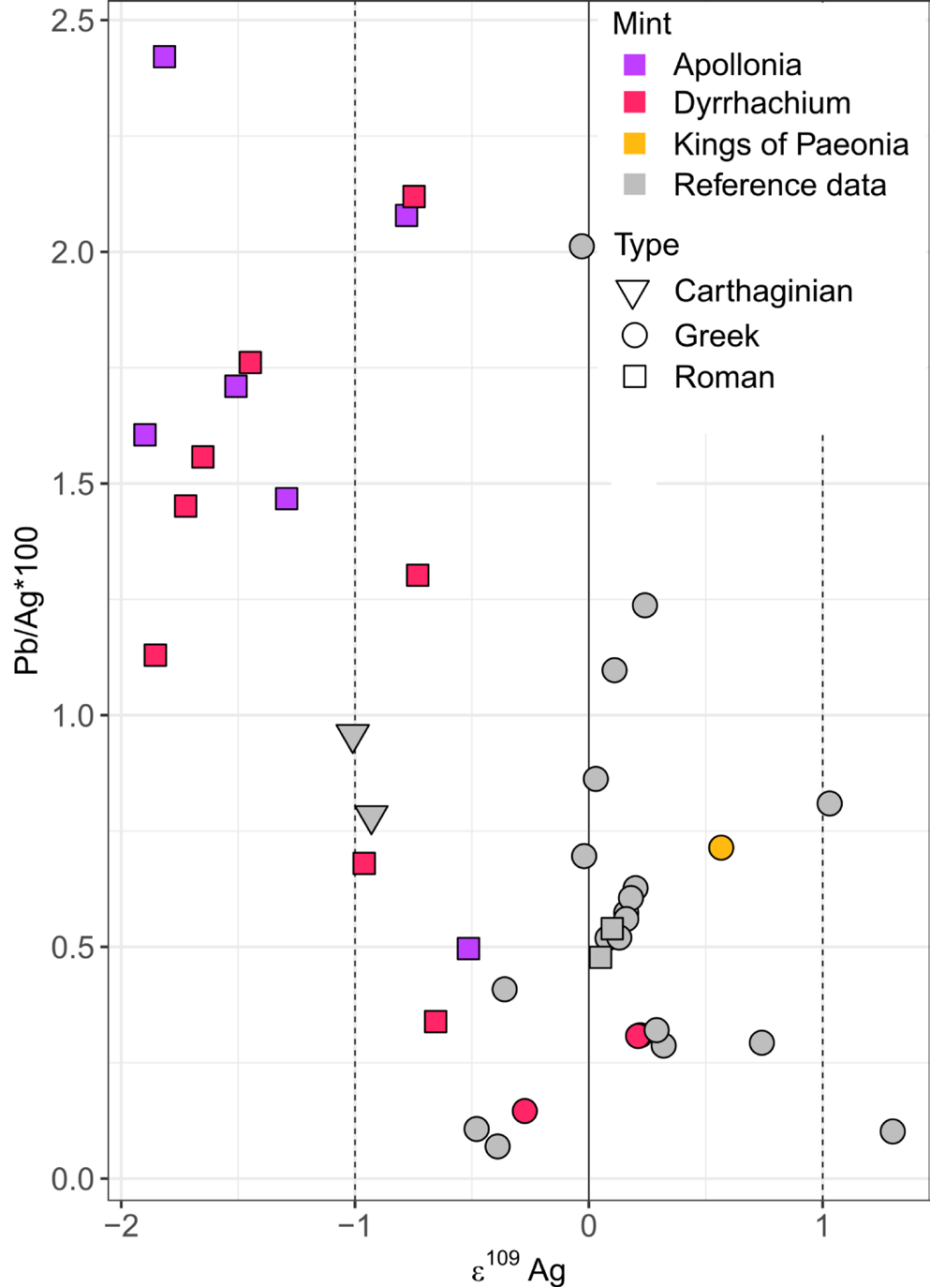
Diagram of Pb/Ag*100 versus ε^109^Ag ratios of the drilled coins issued by Apollonia, Dyrrhachium, and the Kings of Paeonia compared to data from Greek city-states, Rome, and Carthage (Albarède et al. 2021; Birch et al. 2020a, b; Westner et al. 2020). The colour of the symbols corresponds to their minting authority, and the fill colour distinguishes autonomous from Roman surrogate issues and literature data. The ε^109^Ag range (-1 to + 1) of most silver coins analysed so far is indicated with stippled lines

The Roman surrogate coinages generally are distinguished from the earlier issues by their variable, and partially heavily debased, bullion compositions (Cu/Ag*100 ratios between 0.5 and 30) and their mostly comparatively light Ag isotope ratios (< -1 ε^109^Ag) that are coupled with Pb/Ag*100 ratios > 1, which furthermore set them apart from data of Greek, Carthaginian, and Roman silver coinage struck before the end of the Second Punic War (Fig. 8). In contrast, Ag isotope compositions of autonomous issues (-0.66 to + 0.21 ε^109^Ag) and of a coin minted in the name of the Paeonian king Audoleon (ε^109^Ag = + 0.57) fully overlap with the Ag isotopic range (ε^109^Ag= -1 to + 1) of most ancient coinage published so far (Albarède et al. 2016, 2021; Desaulty et al. 2011; Milot et al. 2021a; Vaxevanopoulos et al. 2022b). The absence of correlation between the Cu/Ag ratios and the Pb isotope signatures emphasises that debasement with copper is generally unlikely to alter the isotopic composition of silver bullion due to the low solubility of lead in copper (Chakrabarti and Laughlin 1984; Gentelli et al. 2021) and the oversaturation of lead in cupelled silver (Albarède et al. 2023).

## Discussion

### Relations between minting centres and contemporaneous coin circulation

The Pb isotope data of coins from the Balkan interior generally demonstrate their close relation. This is particularly evident for coins from the “Derrones (?)” and “Laeaei (?)” and Kings of Paeonia data sets, whose Pb isotope signatures exhibit a high degree of mixing with virtually identical end-members. Reuse of coins from the first issuing power that minted the anepigraphic small denominational coinage by the Kings of Paeonia as the latest one active in the region constitutes a historically plausible scenario to explain the overlapping Pb isotope ratios. Recycling by melting down locally abundant metal, for example in the form of silver coins, might have also been relevant for the anepigraphic coinage dated to the first half of the 5th century BCE investigated in this study. Additional analytical data, particularly of large tribal denominations, are warranted to assess material connections as well as to potentially further elucidate the background of the small-denominational anepigraphic coins of northern Greece and the Balkan interior.

Permissible sources for the high-^206^Pb/^204^Pb end-member concentrate in the ancient regions of Macedonia, Thrace, and Paeonia in agreement with the concept of a broader Pangaeon numismatic district (cf. Chapter 1). They also include argentiferous mineralisation at Zletovo in North Macedonia, which is situated amidst the findspot distribution of the coins, raising the possibility of local pre-Roman mining far within the interior of Paeonia. The low-^206^Pb/^204^Pb end-member could not be convincingly identified. Attempts to narrow down potential localisations presumably are furthermore hampered by the presence of several ore sources contributing to isotopic noise surrounding the end-members. While the majority of the known and volumetrically most important Pb-Ag deposits of the Balkan Peninsula were formed in connection with the Alpine orogeny (Heinrich and Neubauer 2002), the complex geology of the region (Schmid et al. 2020) might have preserved remnants of ore-forming events characterised by geologically older Pb isotope compositions. They either have not been identified so far, have not been described in modern literature due to their comparatively small size, or have been mined out since antiquity. The similarity of end-members determined for the “Derrones (?)” and “Laeaei (?)” and Kings of Paeonia data sets to those calculated for issues from the Macedon kingdom and Thasos (Fig. 3) corroborates the hypothesis of one or several bullion sources with Paleozoic Pb isotope model ages in the Balkans (Albarede et al. 2024b). The widespread and chronologically consistent presence of coins closer to the low-^206^Pb/^204^Pb end-member in the Macedonia data set (Albarede et al. 2024a) is in agreement with the history of the region, where deposits previously exploited by local tribes were overtaken by the Macedon kingdom (cf. Chapter 1). Isotopically broadly comparable mineralisations in northern Greece and North Macedonia with local evidence for ancient mining (Kroussia district; Vaxevanopoulos et al., 2022; Westner et al. 2023) provide further arguments that the ore source(s) of this end-member might have been situated close to or within the Macedonian-Paeonian border area.

Overlap of a concentration of “Derrones (?)” coins with coinage from Damastion and Pelagia in cluster dp1 and the comparable Pb isotopic range of bull-type “Derrones (?)” coins to that of dp1 samples (Figure S6; Table S1) indicates that the deposits of Damastion might have been already mined in the first half of the 5th century BCE. Following Strabo’s account, they might have been actively sought for by the Greek colonists from Aegina and Mende that reportedly founded Damastion (cf. Chapter 2). Isotopic overlap suggests contribution of dp1-type metal presumably representing output from Damastion’s mines to the coinage from the Paeonian king Lykkeios in line with the close connection of the minting authorities during this period. The differences in Pb isotope signatures of coinage issued by his successors Audoleon and Patraos in the second half of the 4th century BCE might be linked to disruptions in the raw material supply. In the aftermath of the Celtic migrations, the Paeonian kingdom under its rulers Leon (c. 278/7–250 (?) BCE) and Dropion (c. 250 − 230 BCE) issued only bronze coinage, whose circulation area was limited to today’s southern central North Macedonia (Pavlovska 2011) and thus starkly diminished in comparison to that of its previously issued silver coinage (Fig. 2b). Altogether, this suggests that at some point in the 3rd century BCE, Paeonia no longer had access to silver. The appearance of Damastion issues with geologically younger Pb isotope signatures (dp2), which are notably different from those of earlier issues (dp1), contemporaneous to the observed changes in the bullion provenance of Paeonian coinage, suggests the decline and eventual cessation of local metal production as a possible reason contributing to or causing this development. Decisive factors include silver deposits becoming inaccessible or exhausted, or necessary knowledge, know-how, and infrastructure no longer being available. Furthermore, the Paeonian kingdom apparently did not have sufficient reserves of silver metal that could have been recycled into new coinage issues, and also could not import silver, likely due to its impoverished state (Pavlovska 2008).

Analytical data of Greek city-state coinage from Dyrrhachium (ad1) indicate bullion sources comparable to contemporaneous silver coinage from the Balkan interior. Specifically, a stater minted by Dyrrhachium between c. 340 − 280 BCE (sample M368) has a Pb isotope composition similar to chronologically overlapping issues from Patraos (B016; c. 340 − 315 BCE) and Audoleon (M373; c. 315 − 306 BCE) and a bullion trace element and Ag isotope composition (Cu/Ag*100 = 4.7 and 4.5, and ε^109^Ag=0.21 ± 0.30 and 0.57 ± 0.06, respectively) virtually identical to the latter sample (i.e. M373). An overstrike of a Dyrrhachium issue on a coin from Patraos from the Rezhantsi hoard (IGCH 411; Penchev 2011) further indicates a close relation between coinage from Dyrrhachium and the Kings of Paeonia. The data are too few for an overall assessment of the bullion supply for the autonomous issues from Dyrrhachium (and Apollonia) but generally agree with the hypothesis that silver was sourced from the Balkan interior, thus raising the possibility that the settlements might indeed have functioned as *emporia* for trade with the inland.

Coinage issued by Apollonia and Dyrrhachium as Roman protectorates in cluster ad3 is distinguished from their previous issues as Greek city-states and the rest of the investigated coins by their concentration of isotopic hits in the central Rhodopes. Consequently, the Pb isotope signatures of samples from ad3 are notably different from published Greek coin data (Albarède et al. 2021; Birch et al. 2020a, b; Blichert-Toft et al. 2022; Gale et al. 1980) but comparable to Roman coin data from the second half of the 2nd century BCE (Figure S7; Westner et al. 2020). This suggests that at least the lead was freshly extracted and not recycled. The similarity of Pb isotope ratios of coins from cluster ad2 with that of geologically young end-members calculated for coinage issued by Greek city-states suggests that either the mining districts in the circum-Aegean supplying the bullion were still actively producing metal or that Apollonia recycled silver metal for these series. Results from Pb isotope analysis of Roman lead ingots from an Augustan shipwreck (Bode et al. 2021) indicate that deposits in Chalkidiki and Thasos, which appear as hits for the coins from ad2, were still exploited in the 1st century BCE.

### Types of raw material sources

The change of bullion sources observed for coins from Apollonia and Dyrrhachium is one aspect of a larger transformation that also includes the coin metal composition and ε^109^Ag ratios, which for their previous issues as Greek city-states were aligned with the majority of coinage from the Greek world. The extent of debasement observed for the surrogate coins of Apollonia and Dyrrhachium is uncommon in Greek coinage (Birch et al. 2020a, b; Gale et al. 1980) and the most similar predecessors are Roman victoriati and quadrigati series from the Second Punic War (Burnett 2000; Debernardi et al. 2018), emphasising their affiliation with Roman rather than Greek monetary practice and customs.

The comparatively light Ag isotope ratios (-2 < ε^109^Ag < -1) determined for most semi-autonomous issues from Apollonia and Dyrrhachium can be linked to utilisation of different types of raw materials:

- Tennantite-tetrahedrite compounds [Ag(Sb, As)S_3_] were found by calculation with ab initio methods to generally have low Ag isotope ratios (Fujii and Albarede 2018);
- Cerussite, the most common weathering product of galena, was occasionally found to have ε^109^Ag < -1 (Vaxevanopoulos et al. 2022; Westner et al. 2023); and.
- Galena as the principal Pb ore mineral, particularly comparatively Ag(Sb + As)-poor specimens with Ag contents < 1000 ppm (Westner et al. 2023).

Based on the coupling of elevated Pb contents (Pb/Ag*100 > 1) with ε^109^Ag < -1 in the investigated samples and the deposits determined as permissible sources, the bullion might have been produced by either extracting silver from Pb ore or adding lead of different origins to an argentiferous raw material. In the first case, the ore probably was dominated by galena since cerussite is a poor host for silver (Keim et al. 2016). Exploitation of deposits that primarily produced lead with silver as a by-product was common practice in the Roman period and might have been linked to metallurgical developments (Tylecote 1976). For the second scenario, possible raw materials could be Pb-poor argentiferous ores, including recycled mining debris or beneficiation tailings, to which extraneous lead had to be added for efficient silver collection during smelting. An alternative explanation might be the purification of recalled silver by smelting it with lead metal, as proposed for Roman coinage issued under economic distress during the Second Punic War (Albarède et al. 2016, 2021). Altogether, the fact that the Pb isotope signatures of the surrogate coins are markedly different from Greek coinages but comparable to some contemporaneous Roman denarii (see above), favours the hypothesis that the bullion for these coinages was provided by the Romans (de Callataÿ 2016).

The Ag isotope ratios of the Roman surrogate issues contrast with the unfractionated Ag isotope signatures of most Greek coinages (Albarède et al. 2016, 2021; Desaulty et al. 2011; Milot et al. 2021a; Vaxevanopoulos et al. 2022), which were derived from Ag-rich ores mostly comprising galena and sulphosalt minerals (Milot et al. 2022; Vaxevanopoulos et al. 2022b; Westner et al. 2023). They thus signal the utilisation of a different type of raw material, the concept of which in general dynamically varies as a function of time and place due to, for example, ongoing removal of ore from deposits, newly discovered mineralisations, demands or needs for metal, the technological state of the art, security and the political situation, access to infrastructure, and abundance of skilled workers (Merkel 2020). In itself, the proportion of Pb within silver bullion characterises the cupellation process in which silver is separated from lead, implying that higher Pb contents signal either a lower efficiency and/or a larger scale of the process (cf. Gitler and Ponting 2007).

## Concluding remarks

The largely overlapping Pb isotope ratios of the Balkan interior coinage highlight their close connection, which extends from iconographic and metrological relations to accessed raw material sources, requiring relative spatial proximity. Bullion was produced both as freshly extracted metal, with the mines of Damastion playing a particular role for local coinage, as well as by recycling of earlier issues, evident for coins struck in the name of the Paeonian king Lykkeios. Permissible silver sources concentrate in the ancient regions of Macedonia, Thrace, and Paeonia and include deposits far in the interior of the Balkan Peninsula. In light of the observation from large-scale studies of Greek coinage that mints strike locally sourced metal whenever possible (Albarede et al. 2024a), argentiferous mineralisation nearby and within the findspot distribution of the coins should take precedence in the interpretation. As such, Pb isotope provenance studies can indirectly indicate possible local pre-Roman silver mining and production, and provide valuable insight to identify target areas for field work to test these hypotheses, e.g. in the Kroussia (northern Greece, North Macedonia), Zletovo (North Macedonia), and Novobërdë/Novo Brdo (Kosovo) districts. The available data suggest that the metal sources used for coinages from the Balkan interior were also accessed by the Macedon kingdom, Thasos (Albarede et al. 2024a), and Dyrrhachium, emphasising the importance of the Balkans for the bullion supply of Greek coinage and identifying these mints as potential pathways to distribute metal extracted from these deposits within the circum-Mediterranean silver cycle.

In particular for the investigated coins with geologically older Pb isotope ratios, the interpretation of their likely metal sources was ambiguous. Scattering low-^206^Pb/^204^Pb data suggest that several, possibly rather small-scaled, deposits were primarily exploited. The knowledge of their existence might have been lost because they were considered uneconomic or had been completely mined out. The silver sources used therefore also reflect changes in the types of raw materials as well as possibly of the technology and scale of mining and metallurgical processes through time, as emphasised by the differences between coinages from Greek city-states and coin series issued under Roman control by Apollonia and Dyrrhachium.

## Electronic supplementary material

Below is the link to the electronic supplementary material.

Supplementary Material 1

Supplementary Material 2

Supplementary Material 3

## Data Availability

All data are provided within the manuscript or supplementary information files.
