# Supplementary material for: Tracing metallurgical links and silver provenance in Balkan coinage (5th -1st centuries BCE)
Source: Archaeol Anthropol Sci. 2024 Nov 13;16(12):198. doi: 10.1007/s12520-024-02106-1 (PMC11561118; doi:10.1007/s12520-024-02106-1)
Supplement: Supplementary file 2 — Supplementary Material 2 [file 12520_2024_2106_MOESM2_ESM.pdf]

**Supplementary material ESM2**

# Reconstructing bullion sources and material connections of silver coinage from local tribes and settlements in the Balkan interior (5th-3rd centuries BCE)

**Archaeological and Anthropological Sciences**

Katrin Julia Westner*, Janne Blichert-Toft, Liesel Gentelli, Eftimija Pavlovska, François de Callataÿ, Francis Albarède

*Corresponding author; Ecole Normale Supérieure de Lyon and CNRS, Lyon, France; Katrin.Westner@bergbaumuseum.de

*Testing with the mixing algorithm*

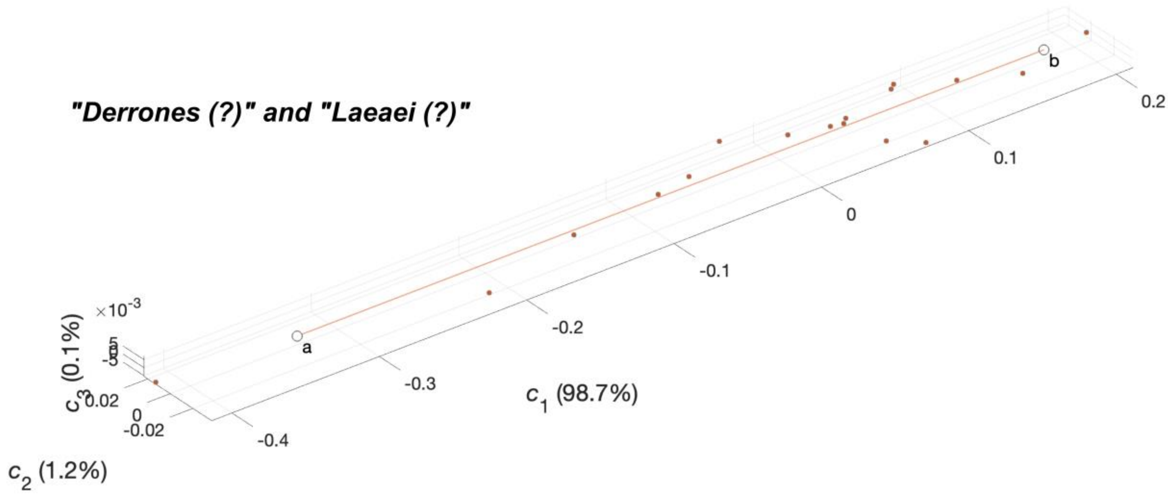


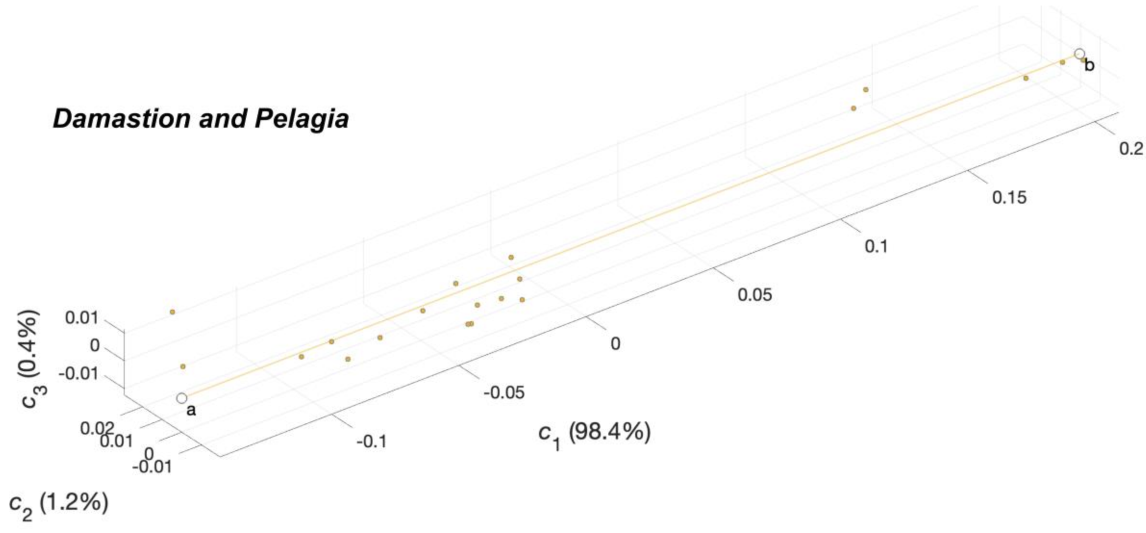


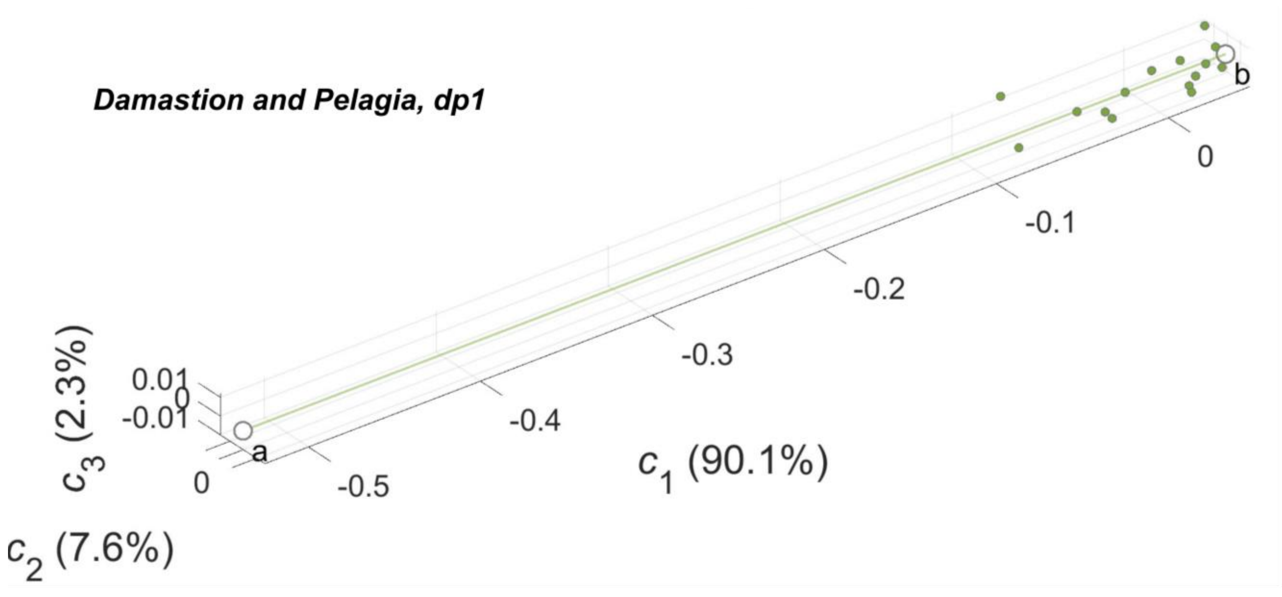

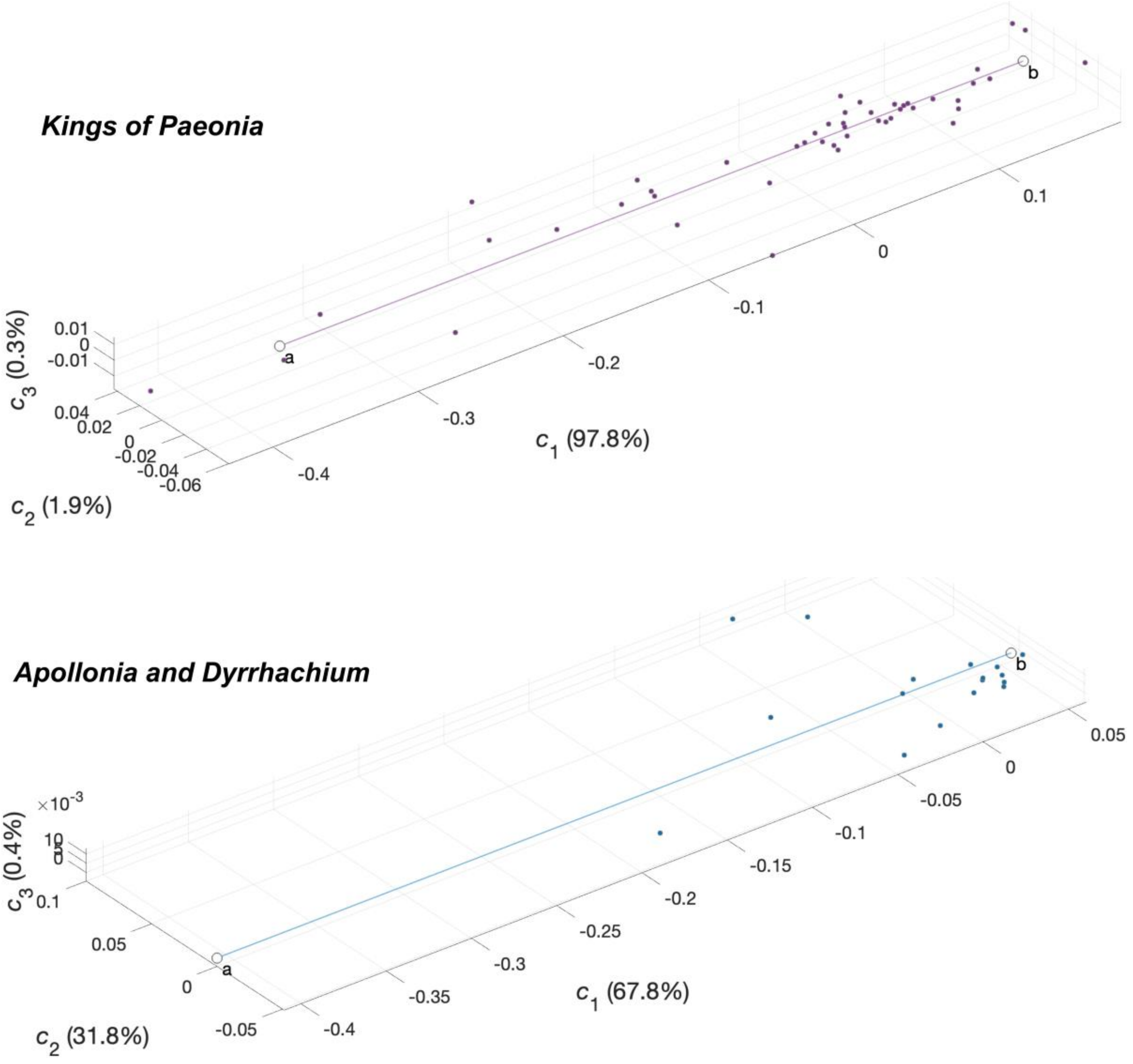


**Fig S2** Graphical representation of the components $c_1$, $c_2$, and $c_3$ calculated by principal component analysis (PCA) of $^{204}Pb$-based isotope data of coins from the four mint groups investigated in this study and cluster dp1. With exception of the Apollonia and Dyrrhachium dataset, the data largely spread along the $c_1$ axis and form a mixing line between raw material sources of the low-$^{206}Pb/^{204}Pb$ end-member a and the high-$^{206}Pb/^{204}Pb$ end-member b (open circles). The coordinates of the end-members were calculated from the fits of Figure S3. Note the gap between the coins of the Damastion and Pelagia dataset on the mixing line and the large distance of the low-$^{206}Pb/^{204}Pb$ end-member a and the coins of cluster dp1.

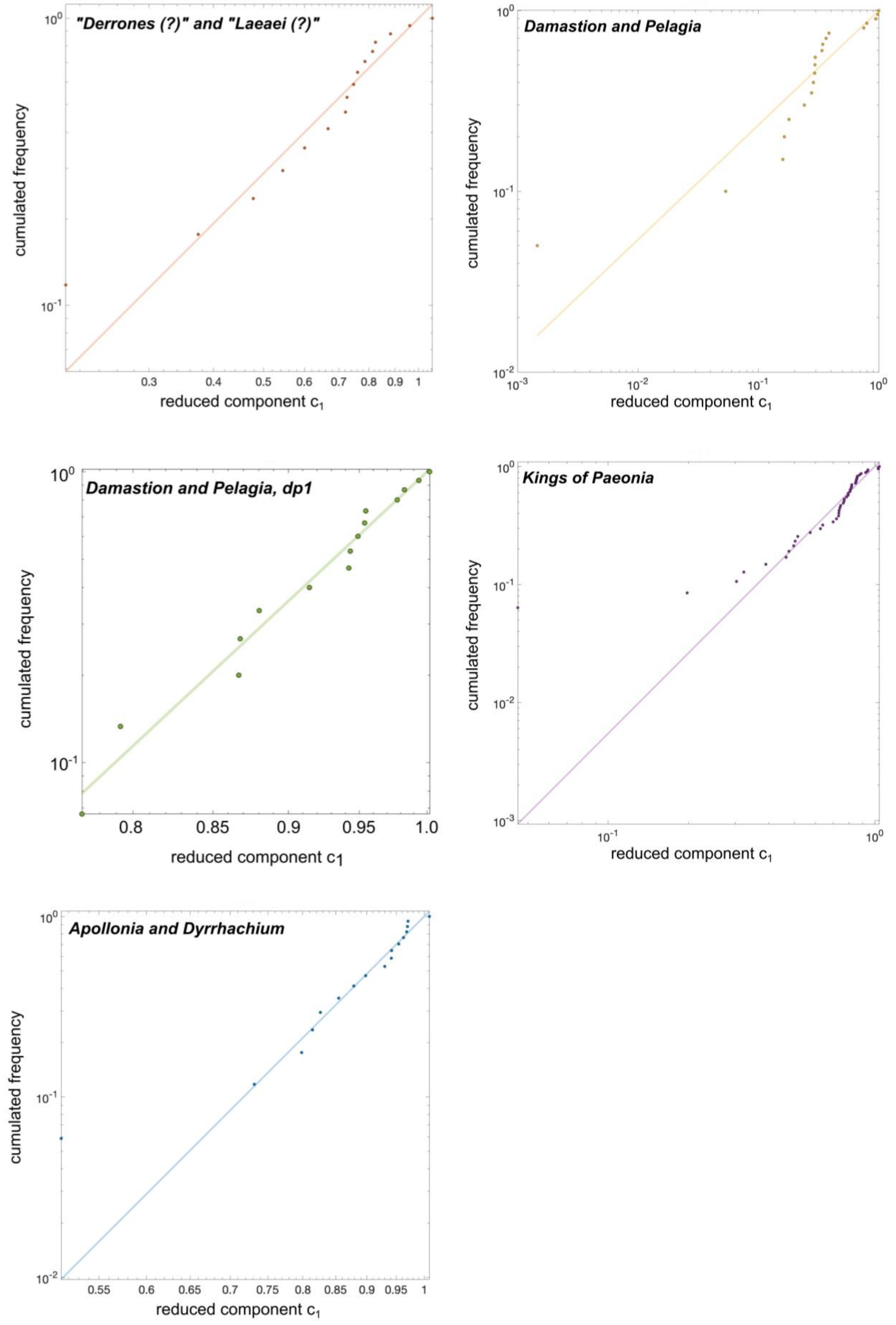


**Fig S3** Fit of the cumulated density function of the reduced component $c_1$ by a power law for the investigated mint groups and cluster dp1. The slope of the regression line is α. The

stronger noise around the straight-line at low values of $c_1$ for the Kings of Paeonia dataset may result from variable proportions of components with low-$^{206}Pb/^{204}Pb$ lead other than the calculated end-member. Note the alignment of the major share of the Damastion and Pelagia dataset (corresponding to cluster dp1 as calculated by PCA), which is seemingly different to the regression line slope excellently fitting the coins closer to the high- $^{206}Pb/^{204}Pb$ end-member (cluster dp2). The alignment is notably better when only the coins from cluster dp1 are considered. See Table S1 for further information on the clusters calculated for the mint groups.

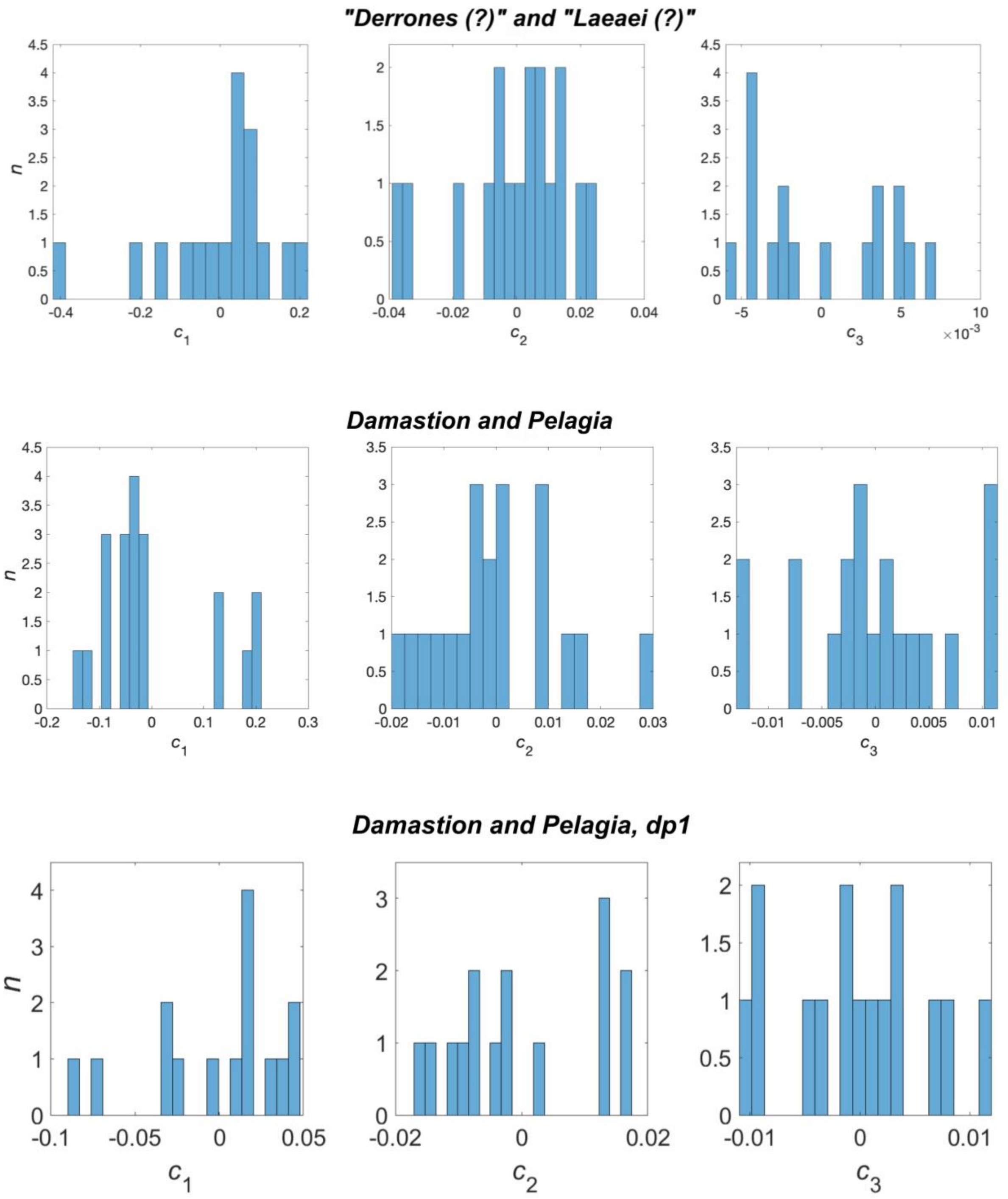

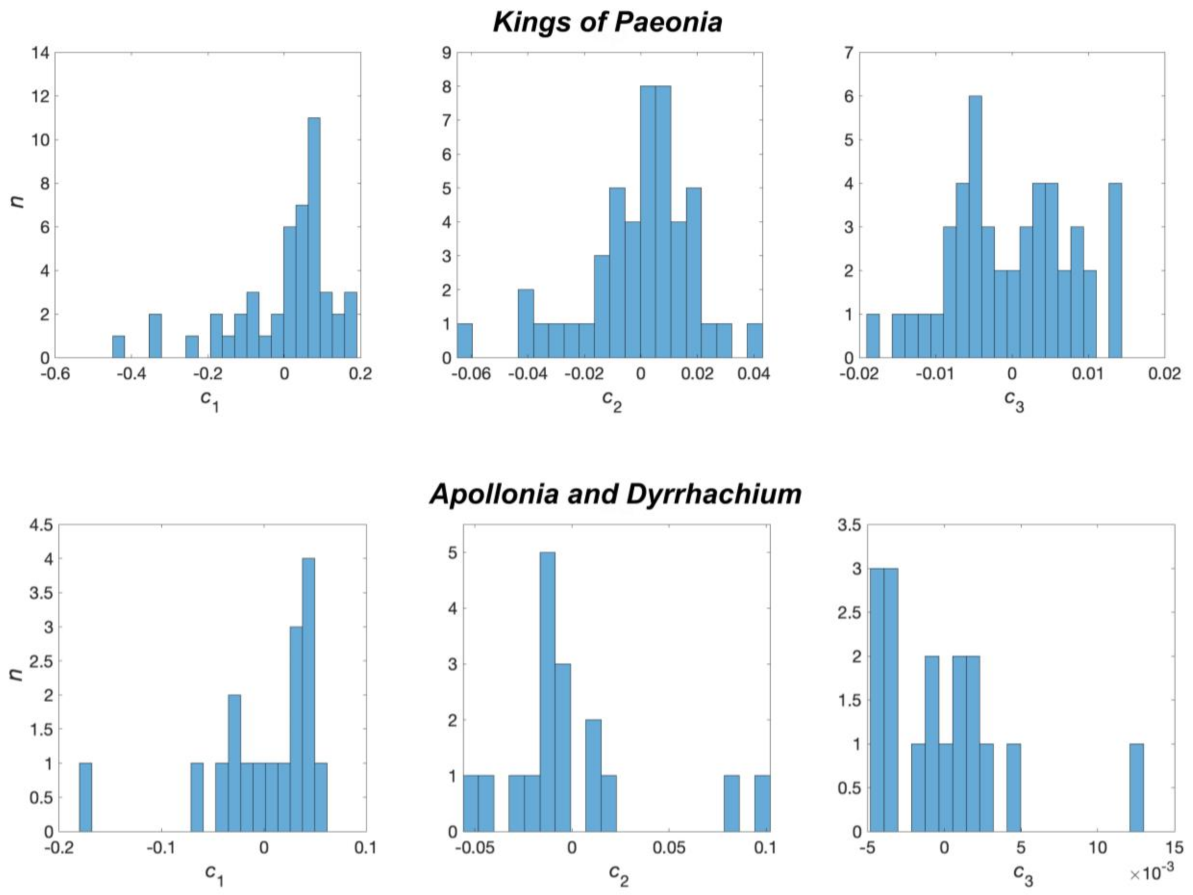


**Fig S4** Distribution of the three component scores $c_1$, $c_2$ and $c_3$ determined by PCA of Pb isotope ratios measured in the coins of the investigated mint groups and cluster dp1. Note the generally skewed distribution of component $c_1$ indicative of mixing. In the “Derrones (?)” and “Laeaei (?)”, and Kings of Paeonia datasets, the highest concentration of coins occurs close to, but not directly at the high- $^{206}Pb/^{204}Pb$ end-member. The distribution of the $c_1$ component is approximately bimodal for the Damastion and Pelagia mint group, akin to the clusters dp1 and dp2 calculated for this dataset. With exception of the Apollonia and Dyrrhachium dataset, the components $c_2$ and $c_3$ are indicative of random noise particularly abundant in the $^{207}Pb/^{204}Pb$ and $^{208}Pb/^{204}Pb$ ratios, which likely is overall increased due to the application of surficial sampling techniques (etching and abrasion) for these coins. Variation in $c_2$ and $c_3$ of the Apollonia and Dyrrhachium coins in general is not random, as expressed by the three clusters ad1, ad2 and ad3 calculated for this dataset.

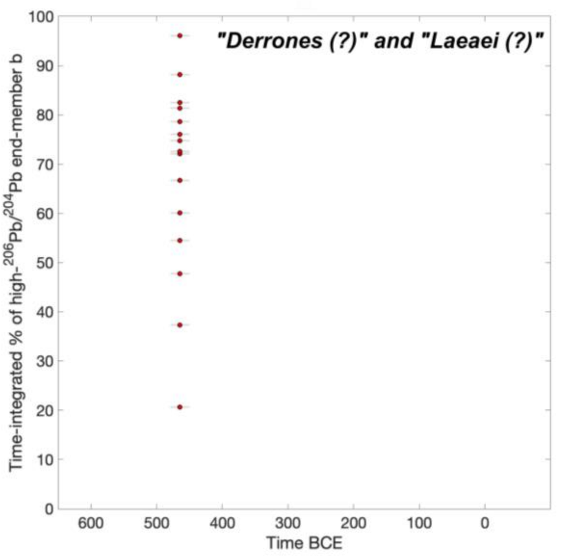
"Derrones (?)" and "Laeaei (?)"
Time-integrated % of high-$^{206}$Pb/$^{204}$Pb end-member b
Time BCE
600 500 400 300 200 100 0
0 10 20 30 40 50 60 70 80 90 100

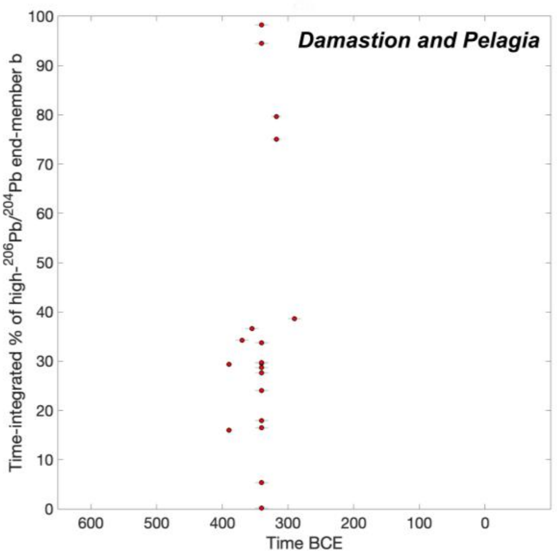
Damastion and Pelagia
Time-integrated % of high-$^{206}$Pb/$^{204}$Pb end-member b
Time BCE
600 500 400 300 200 100 0
0 10 20 30 40 50 60 70 80 90 100

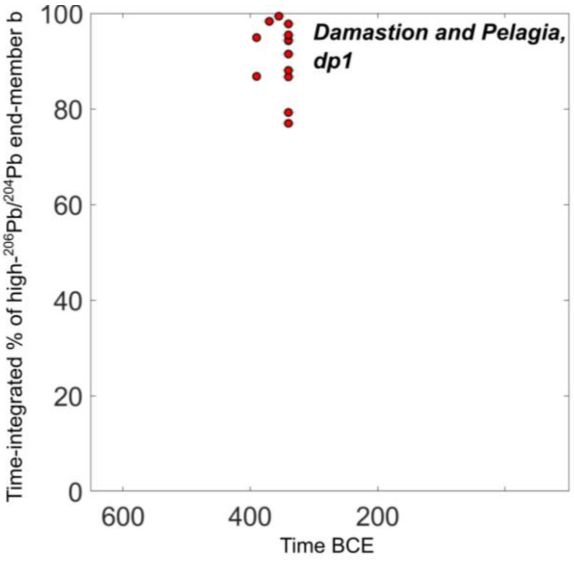
Damastion and Pelagia, dp1
Time-integrated % of high-$^{206}$Pb/$^{204}$Pb end-member b
Time BCE
600 400 200
0 20 40 60 80 100

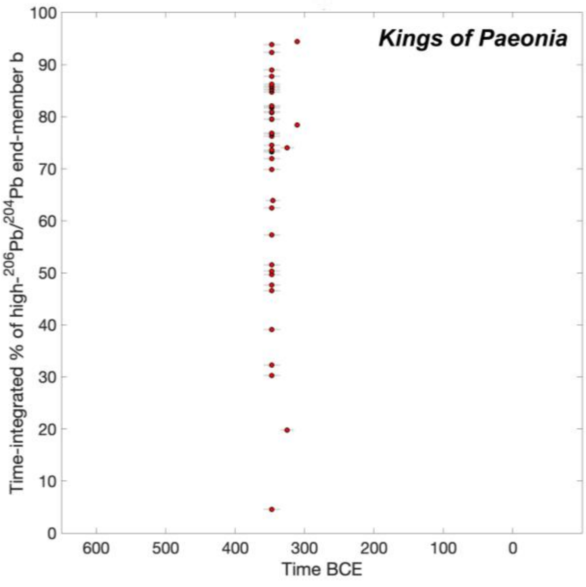
Kings of Paeonia
Time-integrated % of high-$^{206}$Pb/$^{204}$Pb end-member b
Time BCE
600 500 400 300 200 100 0
0 10 20 30 40 50 60 70 80 90 100

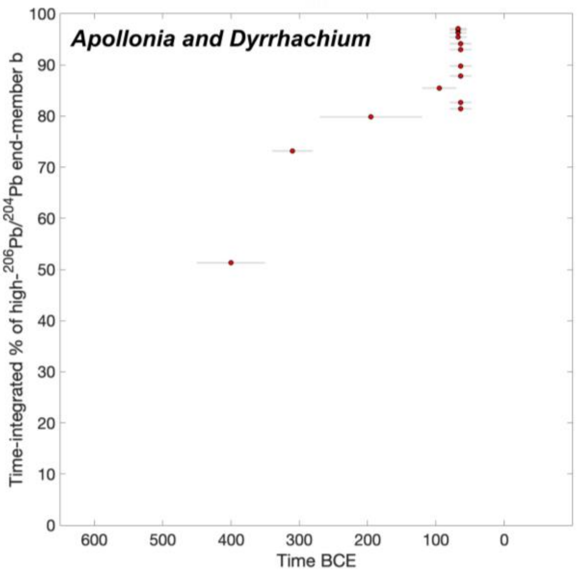
Apollonia and Dyrrhachium
Time-integrated % of high-$^{206}$Pb/$^{204}$Pb end-member b
Time BCE
600 500 400 300 200 100 0
0 10 20 30 40 50 60 70 80 90 100

**Fig S5** Variation of the time-integrated proportion r of the high-$^{206}Pb/^{204}Pb$ end-member b in the $c_1$ component in the investigated coins from four data sets and cluster dp1 relative to their chronological dating. Horizontal bars indicate the range of minting dates for each coin. In the Kings of Paeonia and Damastion and Pelagia datasets, larger proportions of the high-$^{206}Pb/^{204}Pb$ end-member are present in coins with a comparatively young dating. When only considering the coins of cluster dp1, the proportion of the high-$^{206}Pb/^{204}Pb$ end-member b in the $c_1$ component does not fall below c. 75%, rendering the low-$^{206}Pb/^{204}Pb$ end-member insufficiently specified. Note the clear chronological relation of Pb isotope ratios in the Apollonia and Dyrrhachium dataset, distinguishing coins pre- and post-dating the cities becoming Roman protectorates.

*Lead isotope diagrams of coins from specific minting authorities*

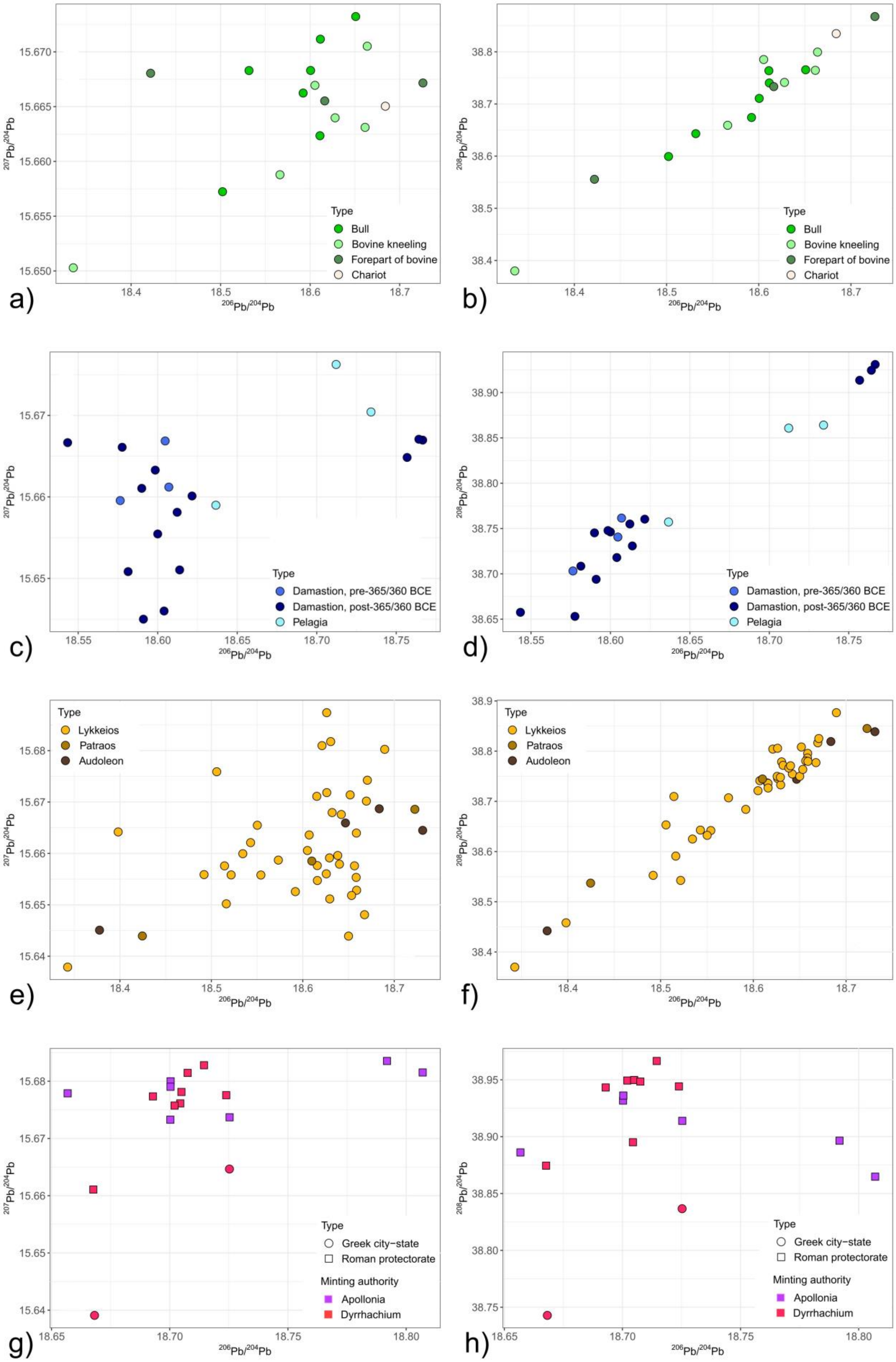

**Fig S6** Diagrams of $^{204}$Pb-based Pb isotope ratios of coins from the investigated mint groups. Symbols are coloured (and shaped in the case of Apollonia and Dyrrhachium) according to chronological and typological differences discussed in the text. Note the different scales of the diagrams. a) and b) “Derrones (?)” and “Laeaei (?)”; c) and d) Damastion and Pelagia; e) and f) Kings of Paeonia; g) and h) Apollonia and Dyrrhachium

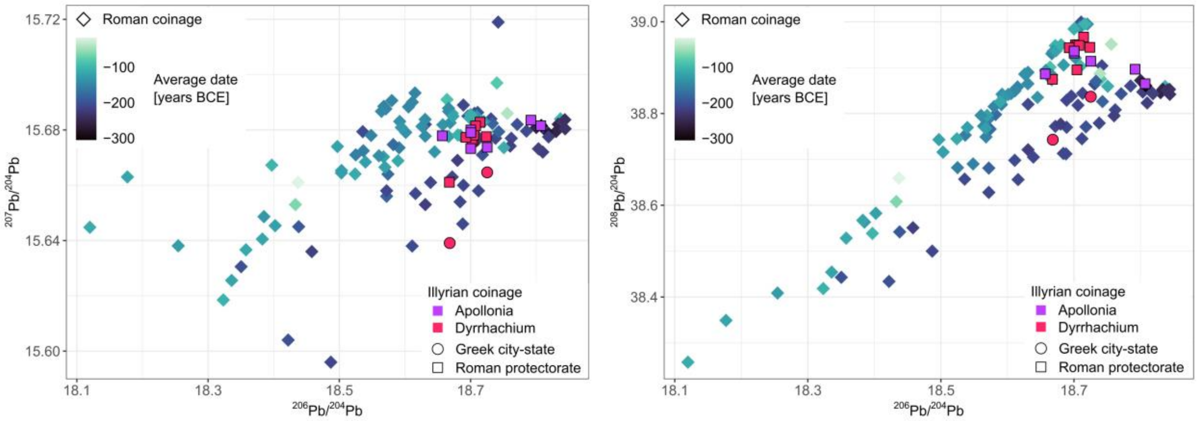


**Fig S7** Diagrams of $^{204}$Pb-based Pb isotope ratios of coins from Apollonia and Dyrrhachium in comparison with silver coinage issued by Rome. For coins from Apollonia and Dyrrhachium, the shape and fill colour of symbols distinguishes minting periods (as Greek city-states and Roman protectorates) and issuing authorities. The colour of symbols representing Roman silver coinage is according to their average dating. Coins from Apollonia and Dyrrhachium in cluster ad3 (see Table S1 for the calculated clusters) dated between the end of the 2nd century BCE and the first half of the 1st century BCE are isotopically similar to contemporaneous Roman coinage. Furthermore, coins from cluster ad2 are comparable to Roman coinage mainly dated before the Second Punic War (218-201 BCE), while coins from cluster ad1 partially overlap with Roman coins issued mostly during the Second Punic War. Reference data for Roman coinage was taken from Albarède et al. (2016), Desaulty et al. (2011), Orejas Saco del Valle et al. (2015) and Westner et al. (2020).

## References

Albarède F, Blichert-Toft J, Rivoal M, Telouk P (2016) A glimpse into the Roman finances of the Second Punic War through silver isotopes. Geochem Persp Let 2:127–137. https://doi.org/10.7185/geochemlet.1613

Desaulty A-M, Télouk P, Albalat E, Albarède F (2011) Isotopic Ag–Cu–Pb record of silver circulation through 16th–18th century Spain. PNAS 108:9002–9007. https://doi.org/10.1073/pnas.1018210108

Orejas Saco del Valle A, Montero Ruiz I, Álvarez González Y, et al (2015) Roman Denarii from North-Western Hispania, Findings from Castromaior (Lugo). A Contextual, Numismatic and Analytic Approach. Madr Mitt 56:232–257

Westner KJ, Birch T, Kemmers F, et al (2020) Rome´s Rise to Power. Geochemical Analysis of Silver Coinage from the Western Mediterranean ($4^{th}$ to $2^{nd}$ Centuries BCE). Archaeometry 62:577–592. https://doi.org/10.1111/arcm.12547
