# Supplementary material for: Tracing metallurgical links and silver provenance in Balkan coinage (5th -1st centuries BCE)
Source: Archaeol Anthropol Sci. 2024 Nov 13;16(12):198. doi: 10.1007/s12520-024-02106-1 (PMC11561118; doi:10.1007/s12520-024-02106-1)
Supplement: Supplementary file 3 — Supplementary Material 3 [file 12520_2024_2106_MOESM3_ESM.zip › ESM3/Westner et al._Supplementary material ESM3.pdf]

**Supplementary material ESM3**

# Reconstructing bullion sources and material connections of silver coinage from local tribes and settlements in the Balkan interior (5th-3rd centuries BCE)

**Archaeological and Anthropological Sciences**

Katrin Julia Westner*, Janne Blichert-Toft, Liesel Gentelli, Eftimija Pavlovska, François de Callataÿ, Francis Albarède

*Corresponding author; Ecole Normale Supérieure de Lyon and CNRS, Lyon, France; Katrin.Westner@bergbaumuseum.de

## Description

ESM3 comprises interactive conventional Pb isotope plots comparing coin and ore reference data from Aegean Greece and Turkey, and the Balkans, which are considered to be the regions the bullion of the investigated coins was most likely derived from. Each set of $^{204}$Pb-based plots corresponds to a modern country to facilitate readability of data. Coin data are shown as grey symbols whose shape refers to the mint groups described in chapter 2 (ad: Apollonia and Dyrrhachium; dl: "Derrones (?)" and "Laeaei (?)"; dp: Damastion and Pelagia; kp: Kings of Paeonia). Ore reference data are shown as circles whose fill colour refers to different mining districts within the countries. Serbia and Kosovo were grouped together as they share several districts. If available, reference data were compiled using TerraLID (prototypes were published under the name GlobaLID; cf. Klein et al., 2022; Westner et al., 2023).

Furthermore, ESM3 comprises png and kml files showing the permissible sources of individual coins and calculated end-members (according to Albarede et al. 2024a) determined by a distance-based algorithm (Albarede et al. 2024b).

## References

Albarede F, Davis G, Gentelli L, et al (2024a) Bullion mixtures in silver coinage from ancient Greece and Egypt. Journal of Archaeological Science 162:105918. https://doi.org/10.1016/j.jas.2023.105918

Albarede F, Davis G, Blichert-Toft J, et al (2024b) A new algorithm for using Pb isotopes to determine the provenance of bullion in ancient Greek coinage. Journal of Archaeological Science 163:105919. https://doi.org/10.1016/j.jas.2023.105919

Akiska S, Demirela G, Sayili S (2013) Geology, mineralogy and the Pb, S isotope study of the Kalkım Pb–Zn ± Cu deposits, Biga Peninsula, NW Turkey. Jour Geosci 379–396. https://doi.org/10.3190/jgeosci.154

Amov BG (1999) Lead isotope data for ore deposits from Bulgaria and the possibility for their use in archaeometry. Berl Beitr Archäometrie 16:5–19

Amov B, Arnaudov V, Pavlova M, et al (1981) Lead isotope data on the Paleozoic granitoids and ore mineralizations from the Western Balkan Mountains and the Trân District (West Bulgaria). 1. Isotopic ratios and geochronology. Geol Balc 11:3–26
Amov B, Kolkovski B, Dimitrov R (1993) Генезис и възраст на хидротермални рудни минерализации в родопската металогенна зона въз основа на изотопния състав на оловото в галенит [Genesis and age of hydrothermal ore mineralization in the Rhodope metallogenic zone on the basis of the isotopic composition of lead in galena]. Annu Univ Sofia “St Kliment Ohridski”, Fac Geol Geogr, Livre 1 85:73–98
Barnes IL, Shields WR, Murphy TJ, Brill RH (1974) Isotopic analyses of Laurion lead ores. In: Beck CW (ed) Archaeological Chemistry. Washington D.C., pp 1–10
Baron S, Tămaş CG, Cauuet B, Munoz M (2011) Lead isotope analyses of gold–silver ores from Roşia Montană (Romania): a first step of a metal provenance study of Roman mining activity in Alburnus Maior (Roman Dacia). J Archaeol Sci 38:1090–1100. https://doi.org/10.1016/j.jas.2010.12.004
Begemann F, Schmitt-Strecker S, Pernicka E (2003) On the composition and provenance of metal finds from Besiktepe (Troia). In: Wagner GA, Pernicka E, Uerpmann HP (eds) Troia and the Troad: Scientific Approaches. Springer, Berlin, pp 173–201
Bird G, Brewer PA, Macklin MG, et al (2010) Pb isotope evidence for contaminant-metal dispersal in an international river system: The lower Danube catchment, Eastern Europe. Appl Geochemistry 25:1070–1084. https://doi.org/10.1016/j.apgeochem.2010.04.012
Bozkaya G (2011) Sulphur- and lead-isotope geochemistry of the Arapuçandere lead–zinc–copper deposit, Biga Peninsula, northwest Turkey. Int Geol Rev 53:116–129. https://doi.org/10.1080/00206810902945090
Bozkaya G, Gökce A (2009) Lead and Sulfur Isotope Studies of the Koru (Çanakkale, Turkey) Lead-Zinc Deposits. Turkish J Earth Sci 18:127–137. https://doi.org/10.3906/yer-0806-5
Brill RH (1970) Lead and Oxygen Isotopes in Ancient Objects. Philos Trans R Soc A 269:143–164. https://doi.org/10.1098/rsta.1970.0093
Çevrim M (1984) Die Zink-Blei-Vererzungen des Aladağ-Gebietes / Türkei mit Betrachtungen zur Paläokarstentwicklung. PhD thesis, Rheinisch-Westfälisch Technische Hochschule, Aachen
Chalkias G, Vavelidis M, Schmitt-Strecker S, Begemann F (1988) Geologische Interpretation der Blei-Isotopen-Verhältnisse von Erzen der Insel Thasos, der Ägäis und Nordgriechenlands. In: Wagner GA, Weisgerber G (eds) Antike Edel- und Buntmetallgewinnung auf Thasos. Deutsches Bergbau-Museum, Bochum, pp 59–74
Chamberlain VE, Gale NH (1980) The isotopic composition of lead in Greek coins and in galena from Greece and Turkey. In: Proc. 16th Inst. Symp. on Archeometry and Archaeological Prospection, Edinburgh 1976. National Museum of Scotland, Edinburgh, pp 139–155
Chernyshev IV, Kovalenker VA, Chugaev A, et al (2014) New high-precision lead isotope analyses of galena from Romanian ore districts and a review. Rom J Mineral Deposits 87:83–86
Çiçek M, Oyman T (2016) Origin and evolution of hydrothermal fluids in epithermal Pb-Zn-Cu±Au±Ag deposits at Koru and Tesbihdere mining districts, Çanakkale, Biga Peninsula, NW Turkey. Ore Geol Rev 78:176–195. https://doi.org/10.1016/j.oregeorev.2016.03.020
Çiçek M, Oyman T, Palmer MR (2021) Variation of Cu, Fe, S and Pb isotopes in sulfides from hydrothermal mineralization from the Yenice region in Çanakkale, Biga Peninsula, NW Turkey. Ore Geol Rev 136:104255. https://doi.org/10.1016/j.oregeorev.2021.104255
Demirela G, Akıska S (2022) Evaluation of Pb isotope systematics and metal sources of the Biga Pb–Zn Province (NW Turkey) and comparison with the Pb isotope systematics of the Rhodope Massif. J Afr Earth Sci 187:104445. https://doi.org/10.1016/j.jafrearsci.2021.104445
Durali-Müller S (2005) Roman lead and copper mining in Germany their origin and development through time, deduced from lead and copper isotope provenance studies. PhD thesis,

Goethe-Universität. https://publikationen.ub.uni-frankfurt.de/frontdoor/index/index/docId/2824

Frei R (1992) Isotope (Pb, Rb-Sr, S, O, C, U-Pb) geochemical investigations on Tertiary intrusives and related mineralizations in the Serbomacedonian Pb-Zn, Sb+Cu-Mo metallogenetic province in Northern Greece. PhD thesis, ETH Zürich. https://doi.org/10.3929/ethz-a-000692261

Gale NH (1978) Lead Isotopes and Aegean Metallurgy. In: Doumas C (ed) Thera and the Aegean world I. Papers presented at the Second International Scientific Congress, Santorini, Greece, August, 1978. London, pp 529–545

Gale NH (1980) Some aspects of lead and silver mining in the Aegean. In: Doumas C (ed) Thera and the Aegean World II. Proceedings of the Second International Scientific Congress, Santorini, Greece, August 1978. Aris and Phillips Ltd., London, pp 161–195

Gale NH, Gentner W, Wagner GA (1980) Mineralogical and Geographical Silver Sources of Archaic Greek Coinage. In: Metcalf DM, Oddy WA (eds) Metallurgy in Numismatics. London, pp 3–49

Gale NH, Picard O, Barrandon JN (1988) The Archaic Thasian silver coinage. In: Wagner GA, Weisgerber G (eds) Antike Edel- und Buntmetallgewinnung auf Thasos. Deutsches Bergbau-Museum Bochum, Bochum, pp 212–223

Gale NH, Stos-Gale ZA (1985) Cyprus and the Bronze Age Metals Trade. In: Papadopoullos T, Chatzēstyllēs SA (eds) Proceedings of the Second International Congress of Cypriot Studies, Nicosia 1982. Society of Cypriot Studies, Nicosia, pp 51–66

Hirao Y, Enemoto J, Tachikawa H (1995) Lead Isotope Ratios of Copper, Zinc and Lead Minerals in Turkey – in Relation to the Provenance Study of Artefacts. In: Mikasa T, Prince HIH (eds) Essays on Ancient Anatolia and its Surrounding Civilizations. Otto Harrassowitz, Wiesbaden, pp 89–114

Kalogeropoulos SI, Kilias SP, Bitzios DC, et al (1989) Genesis of the Olympias carbonate-hosted Pb-Zn (Au, Ag) sulfide ore deposit, eastern Chalkidiki Peninsula, northern Greece. Econ Geol 84:1210–1234. https://doi.org/10.2113/gsecongeo.84.5.1210

Klein S, Rose T, Westner KJ, Hsu Y-K (2022) From OXALID to GlobaLID: Introducing a modern and FAIR lead isotope database with an interactive application. Archaeometry 64:935–950. https://doi.org/10.1111/arcm.12762

Koptagel O, Ulusoy U, Fallick AE (2007) Sulfur and Lead Isotope Investigations of the Carbonate-Hosted Pb-Zn Deposits in the Yahyalı Region, Kayseri, Southern Turkey. Turkish J Earth Sci 16:57–76

Kouzmanov K, Moritz R, Von Quadt A, et al (2009) Late Cretaceous porphyry Cu and epithermal Cu-Au association in the Southern Panagyurishte district, Bulgaria: the paired Vlaykov Vruh and Elshitsa deposits. Miner Depos 44:611–646. https://doi.org/10.1007/s00126-009-0239-1

Marchev P, Moritz R (2006) Isotopic composition of Sr and Pb in the Central Rhodopean ore fields: Inferences for the genesis of the base-metal deposits. Geol Balc 35:49–61

Marcoux E, Grancea L, Lupulescu M, Milési J (2002) Lead isotope signatures of epithermal and porphyry-type ore deposits from the Romanian Carpathian Mountains. Miner Deposita 37:173–184. https://doi.org/10.1007/s00126-001-0223-x

Nebel ML, Hutchinson RW, Zartman RE (1991) Metamorphism and polygenesis of the Madem Lakkos polymetallic sulfide deposit, Chalkidiki, Greece. Econ Geol 86:81–105. https://doi.org/10.2113/gsecongeo.86.1.81

OXALID: Oxford Archaeological Lead Isotope Database from the Isotrace Laboratory (https://oxalid.arch.ox.ac.uk/default.html)

Özen Y, Arık F (2015) S, O and Pb isotopic evidence on the origin of the İnkaya (Simav-Kütahya) Cu–Pb–Zn–(Ag) Prospect, NW Turkey. Ore Geol Rev 70:262–280. https://doi.org/10.1016/j.oregeorev.2015.04.001

Pernicka E, Begemann F, Schmitt-Strecker S, Wagner GA (1993) Eneolithic and Early Bronze Age copper artefacts from the Balkans and their relation to Serbian copper ores. Prähist Z 68:1–54. https://doi.org/10.1515/prhz.1993.68.1.1
Recław J, Sierpień P, Karasiński J, et al (2024) The origin of lead artifacts from Novae: applications of Pb isotopes in identifying the provenance of Roman artifacts from N. Bulgaria. Herit Sci 12:40. https://doi.org/10.1186/s40494-024-01151-2
Sayre EV, Joel EC, Blackman MJ, et al (2001) Stable Lead Isotope Studies of Black Sea Anatolian Ore Sources and Related Bronze Age and Phrygian Artefacts from Nearby Archaeological Sites. Appendix: New Central Taurus Ore Data. Archaeometry 43:77–115. https://doi.org/10.1111/1475-4754.00006
Seeliger TC, Pernicka E, Wagner GA, et al (1985) Archäometallurgische Untersuchungen in Nord- und Ostanatolien. Jahrb Röm Ger Zent Mus 32:597–569
Siron CR (2018) Magmatic, Structural, and Metallogenic Framework of the Kassandra Mining District, Chalkidiki Peninsula, Northern Greece. PhD thesis, Cornell University
Stos-Gale ZA, Gale NH, Annetts N (1996) Lead isotope data from the Isotrace Laboratory, Oxford: Archaeometry data base 3, ores from the Aegean, part 1. Archaeometry 38:381–390. https://doi.org/10.1111/j.1475-4754.1996.tb00784.x
Tombros SF, Kokkalas S, Seymour KSt, et al (2021) The Kallianos Au-Ag-Te mineralization, Evia Island, Greece: a detachment-related distal hydrothermal deposit of the Attico-Cycladic Metallogenetic Massif. Miner Deposita 56:665–684. https://doi.org/10.1007/s00126-020-00989-3
Ünal-Çakir E, Gökce A (2018) Sulfur and Lead Isotopic Compositions of the Akgüney (Ordu) Cu-Pb-Zn Deposit in the Black Sea Region, Turkey. Bull Earth Sci Appl Res Cent Hacettepe Univ 39:131–140
Vavelidis M, Bassiakos I, Begemann F, et al (1985) Geologie und Erzvorkommen. In: Silber, Blei und Gold auf Sifnos. Deutsches Bergbau-Museum, Bochum, pp 59–80
Vaxevanopoulos M, Blichert-Toft J, Davis G, Albarède F (2022) New findings of ancient Greek silver sources. J Archaeol Sci 137:105474. https://doi.org/10.1016/j.jas.2021.105474
Veselinović-Williams M (2011) Characteristics and origin of polymetallic mineralisation in the Kopaonik region of Serbia and Kosovo, with particular reference to the Belo Brdo Pb-Zn (Ag) deposit. PhD thesis, Kingston University
Wagner GA, Pernicka E, Seeliger TC, et al (1985) Geologische Untersuchungen zur frühen Metallurgie in NW-Anatolien. Bull Miner Res Explor 101/102:45–81
Wagner GA, Pernicka E, Seeliger TC, et al (1986) Archäometallurgische Untersuchungen an Rohstoffquellen des frühen Kupfers Ostanatoliens. Jahrb Röm Ger Zent Mus 36:732–752
Wagner GA, Pernicka E, Vavelidis M, et al (1986) Archäometallurgische Untersuchungen auf Chalkidiki. Der Anschnitt 38:166–186
Westner KJ (2017) Roman mining and metal production near the antique city of ULPIANA (Kosovo). PhD thesis, Johann Wolfgang Goethe-Universität. https://publikationen.ub.uni-frankfurt.de/frontdoor/index/index/docId/44048
Westner KJ, Rose T, Klein S, et al (2023) GlobaLID – Global Lead Isotope Database (Version 12/2023). https://doi.org/10.5880/fidgeo.2023.043
Westner KJ, Vaxevanopoulos M, Blichert-Toft J, et al (2023) Isotope and trace element compositions of silver-bearing ores in the Balkans as possible metal sources in antiquity. J Archaeol Sci 155:105791. https://doi.org/10.1016/j.jas.2023.105791
Wind SC, Schneider DA, Hannington MD, McFarlane CRM (2020) Regional similarities in lead isotopes and trace elements in galena of the Cyclades Mineral District, Greece with implications for the underlying basement. Lithos 105559. https://doi.org/10.1016/j.lithos.2020.105559
Yener KA, Sayre EV, Joel EC, et al (1991) Stable lead isotope studies of Central Taurus ore sources and related artifacts from Eastern Mediterranean Chalcolithic and Bronze Age sites. J Archaeol Sci 18:541–577. https://doi.org/10.1016/0305-4403(91)90053-R
