# Supplementary figures and images for: Tracing metallurgical links and silver provenance in Balkan coinage (5th -1st centuries BCE)
Source: Archaeol Anthropol Sci. 2024 Nov 13;16(12):198. doi: 10.1007/s12520-024-02106-1 (PMC11561118; doi:10.1007/s12520-024-02106-1)

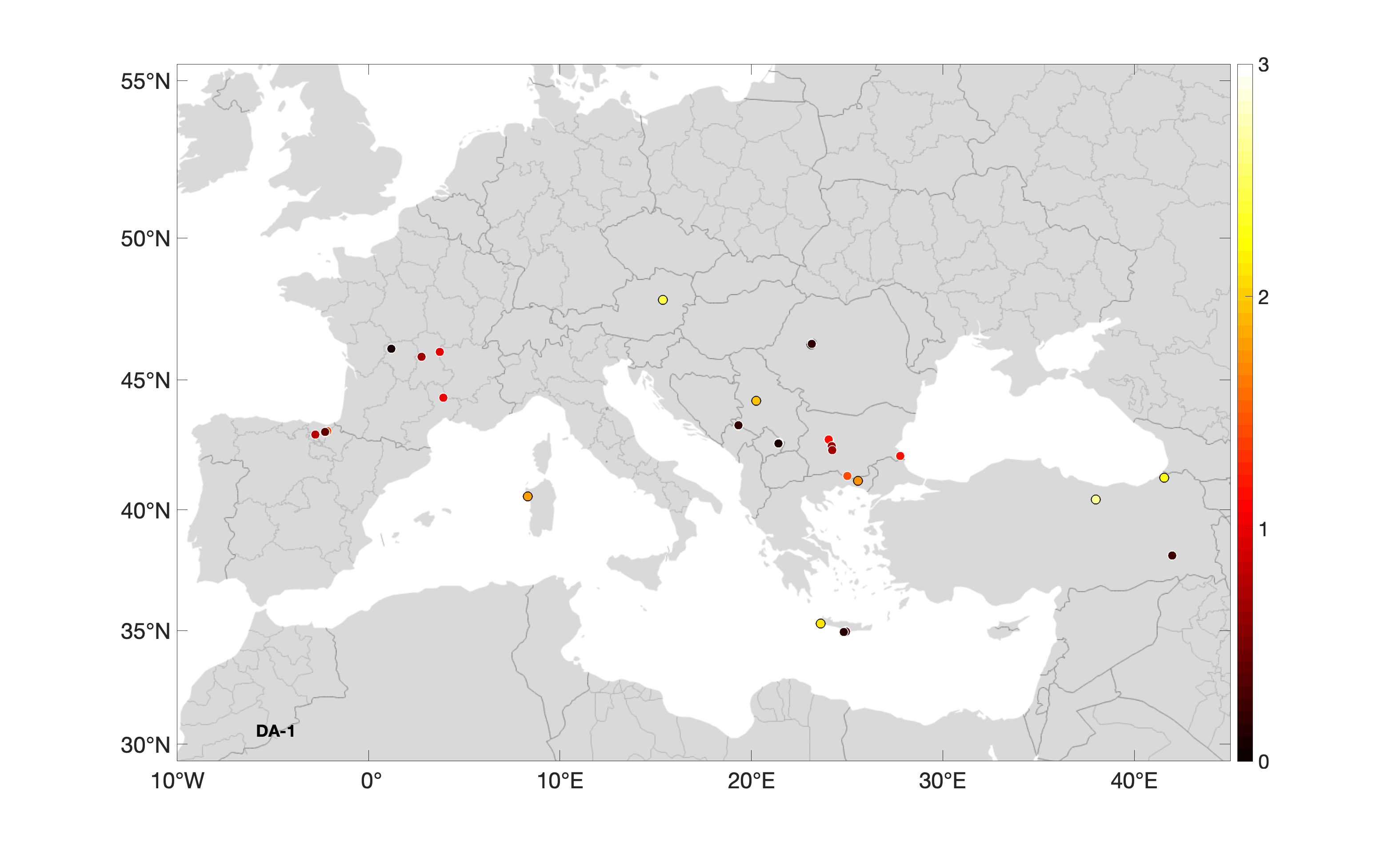

Supplement: Supplementary file 3 — Supplementary Material 3 [file 12520_2024_2106_MOESM3_ESM.zip › ESM3/png_hit maps/DA-1_map_jittered.png]

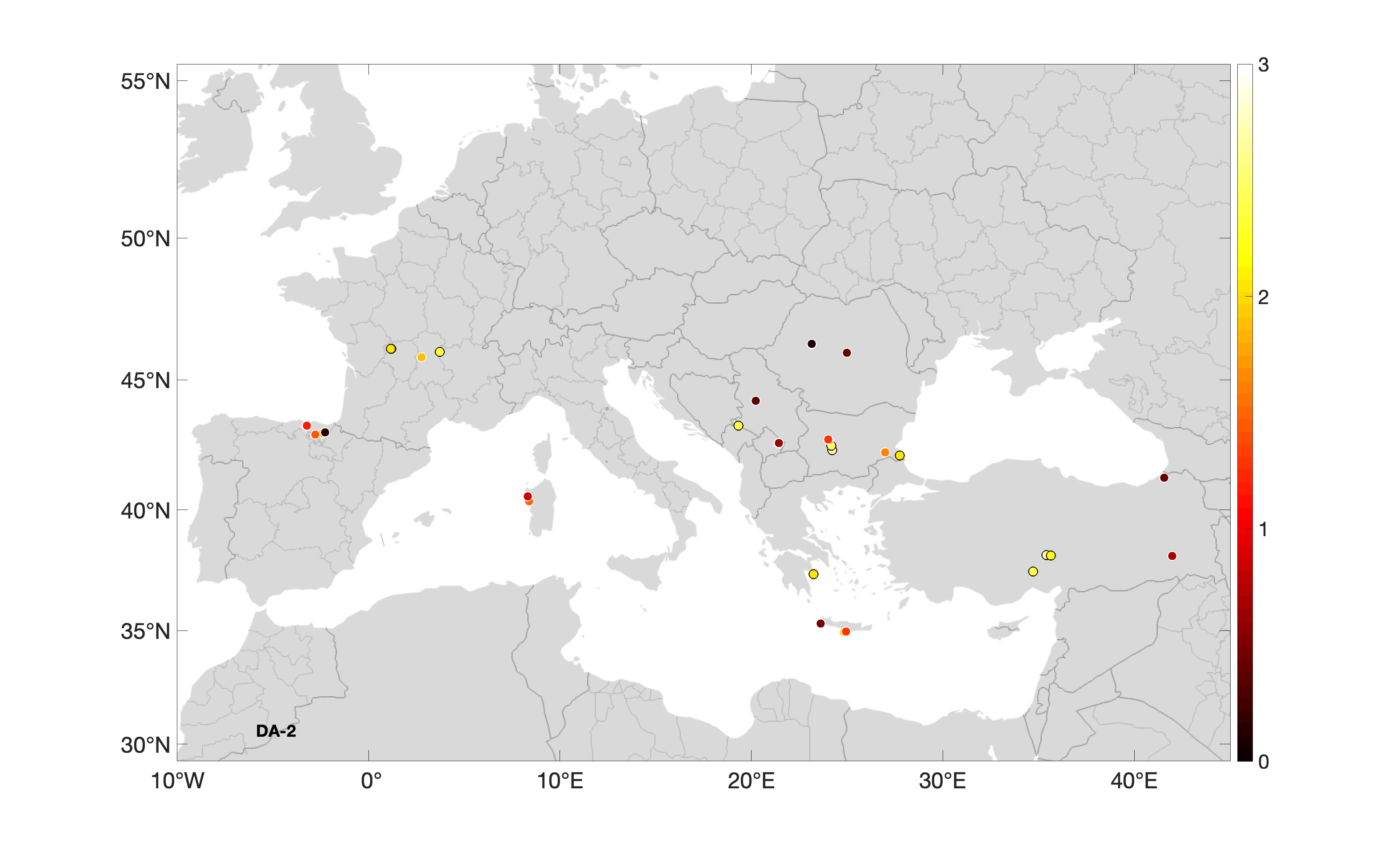

Supplement: Supplementary file 3 — Supplementary Material 3 [file 12520_2024_2106_MOESM3_ESM.zip › ESM3/png_hit maps/DA-2_map_jittered.png]

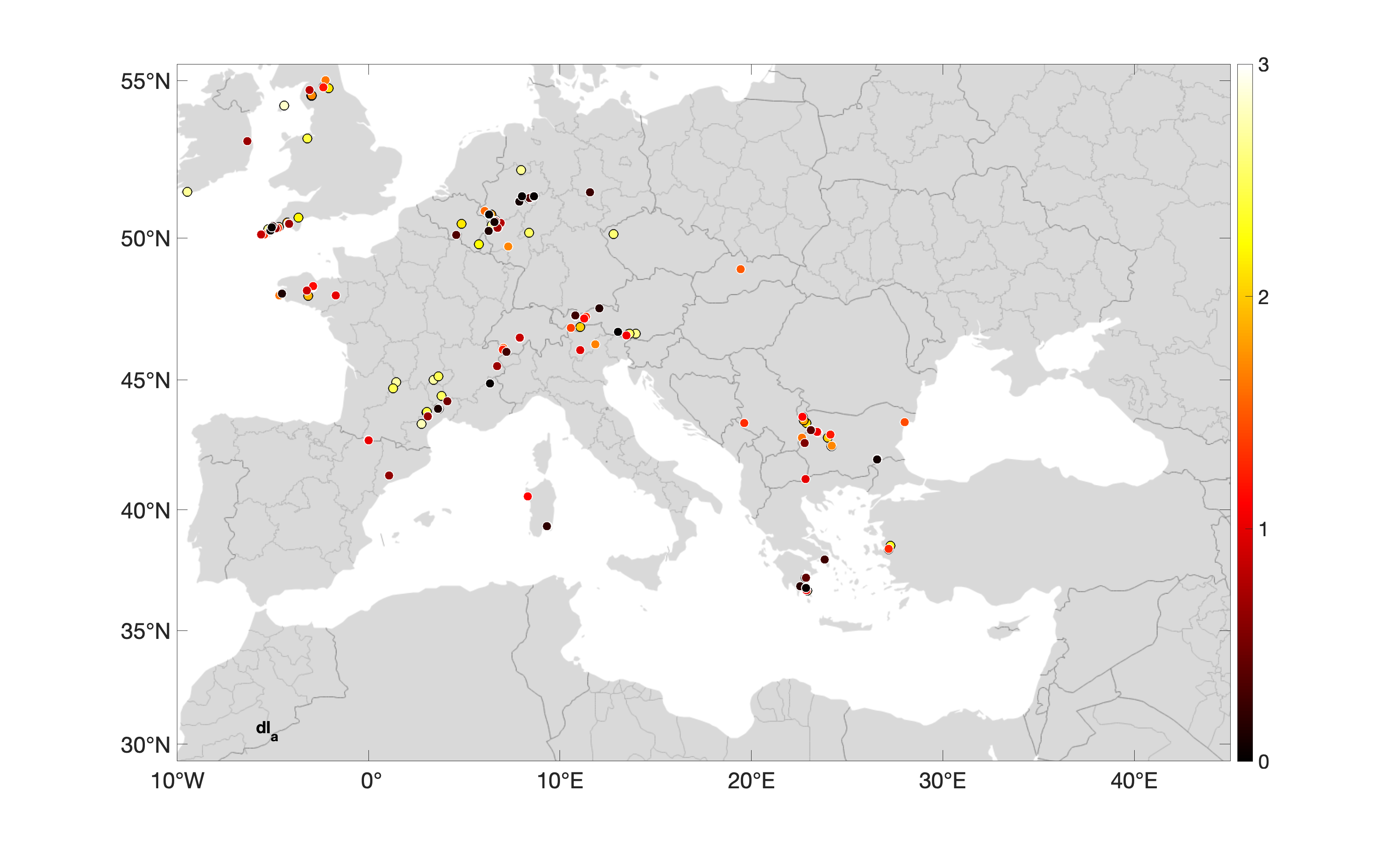

Supplement: Supplementary file 3 — Supplementary Material 3 [file 12520_2024_2106_MOESM3_ESM.zip › ESM3/png_hit maps/dl_a_map_jittered.png]

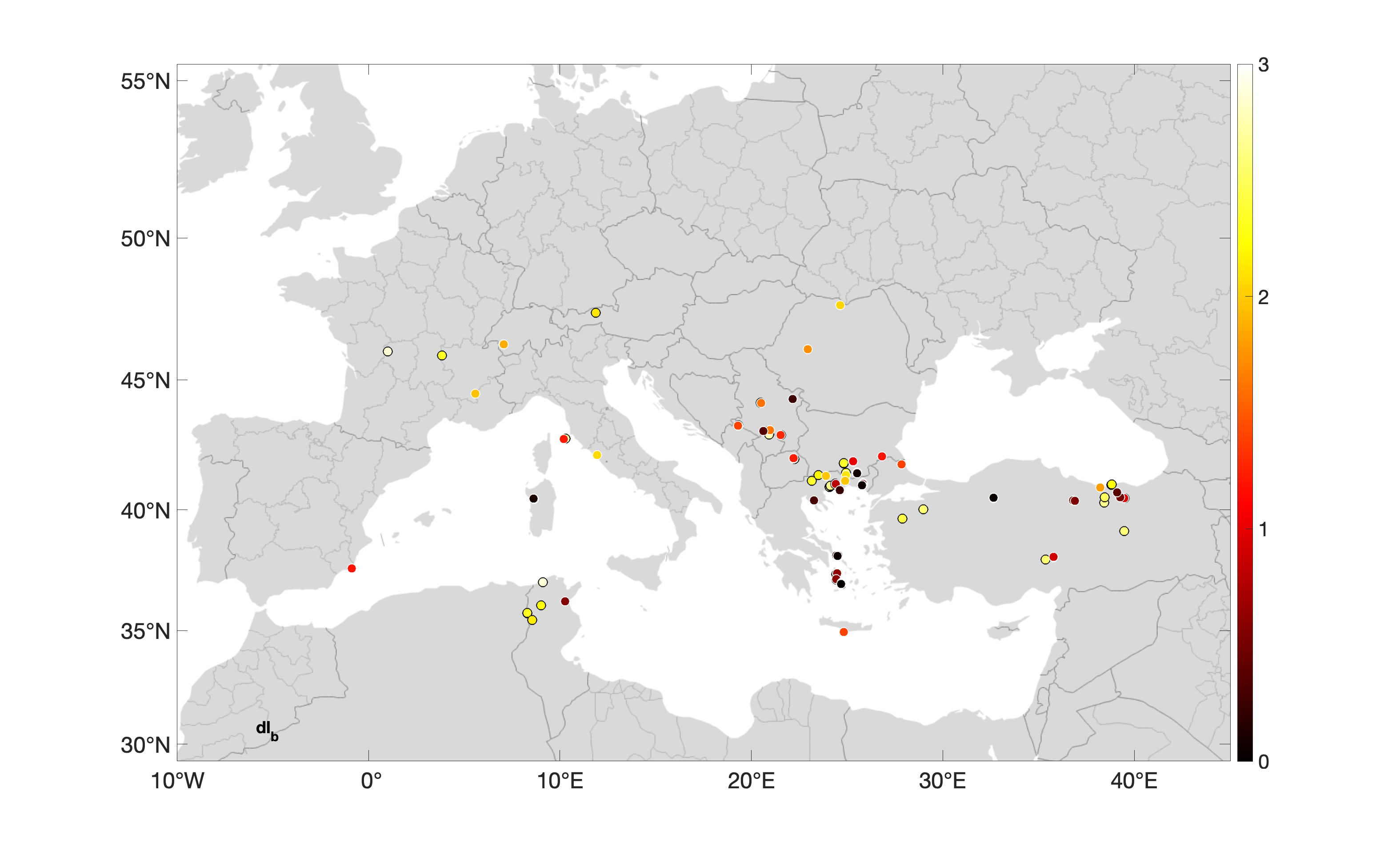

Supplement: Supplementary file 3 — Supplementary Material 3 [file 12520_2024_2106_MOESM3_ESM.zip › ESM3/png_hit maps/dl_b_map_jittered.png]

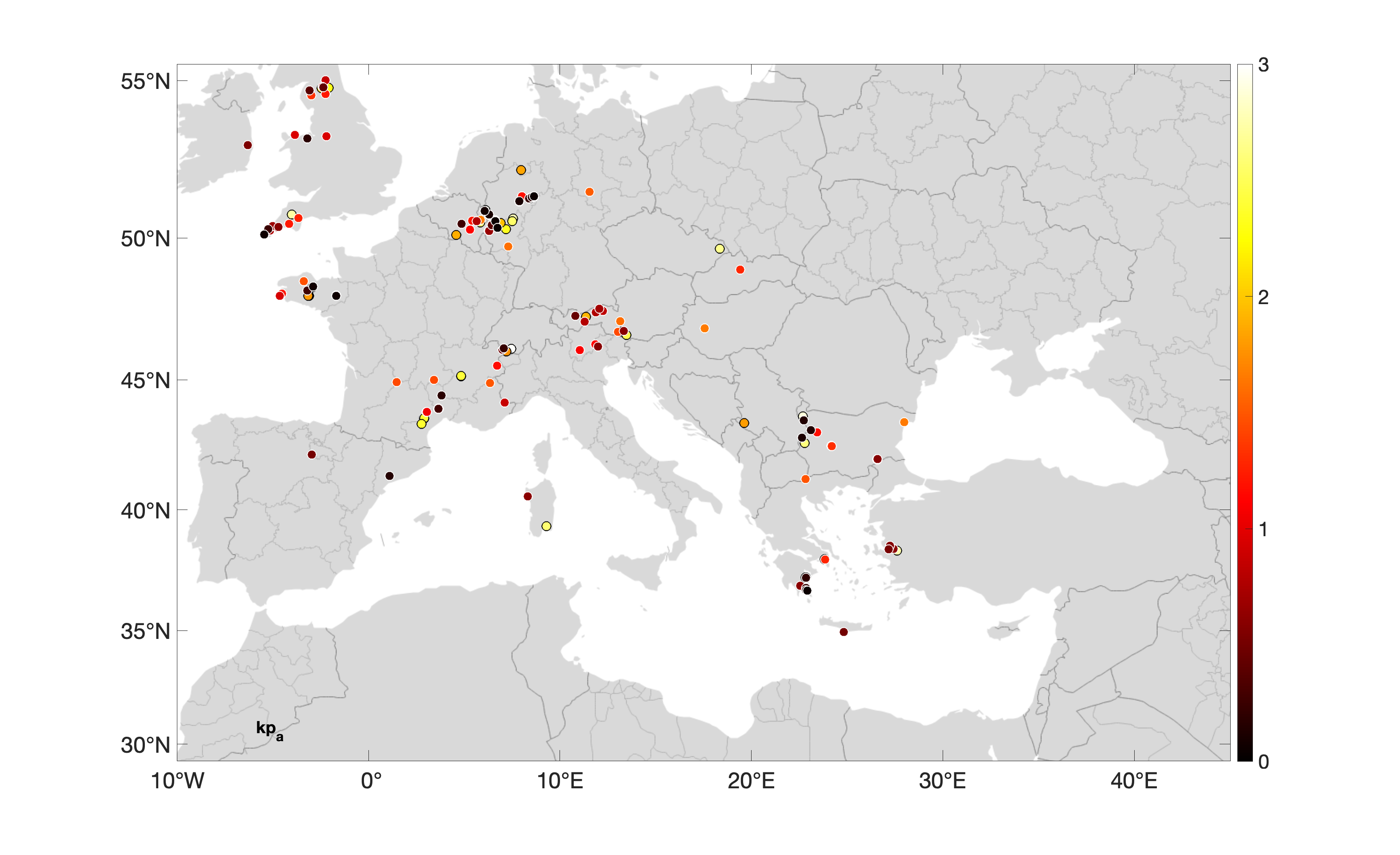

Supplement: Supplementary file 3 — Supplementary Material 3 [file 12520_2024_2106_MOESM3_ESM.zip › ESM3/png_hit maps/kp_a_map_jittered.png]

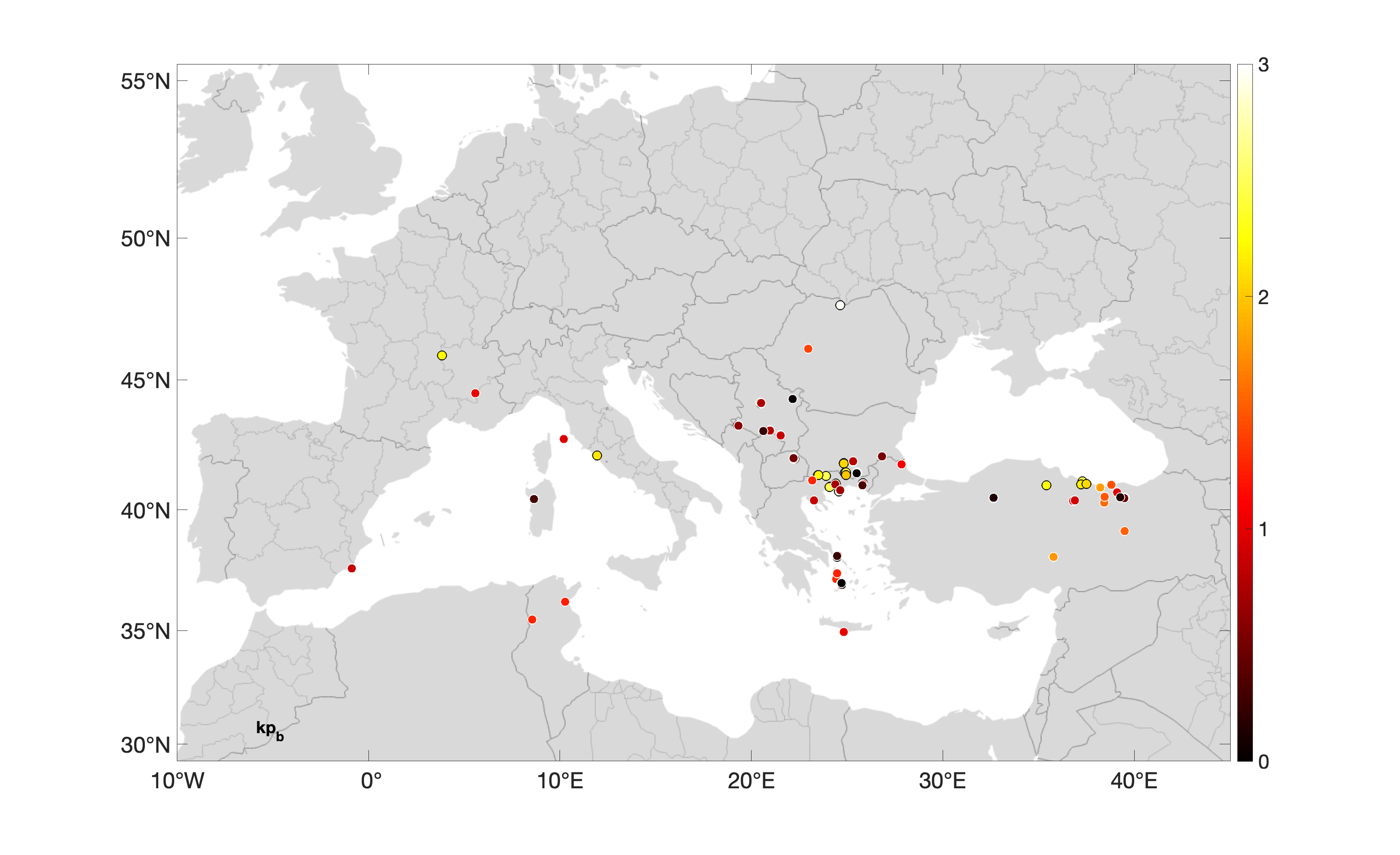

Supplement: Supplementary file 3 — Supplementary Material 3 [file 12520_2024_2106_MOESM3_ESM.zip › ESM3/png_hit maps/kp_b_map_jittered.png]

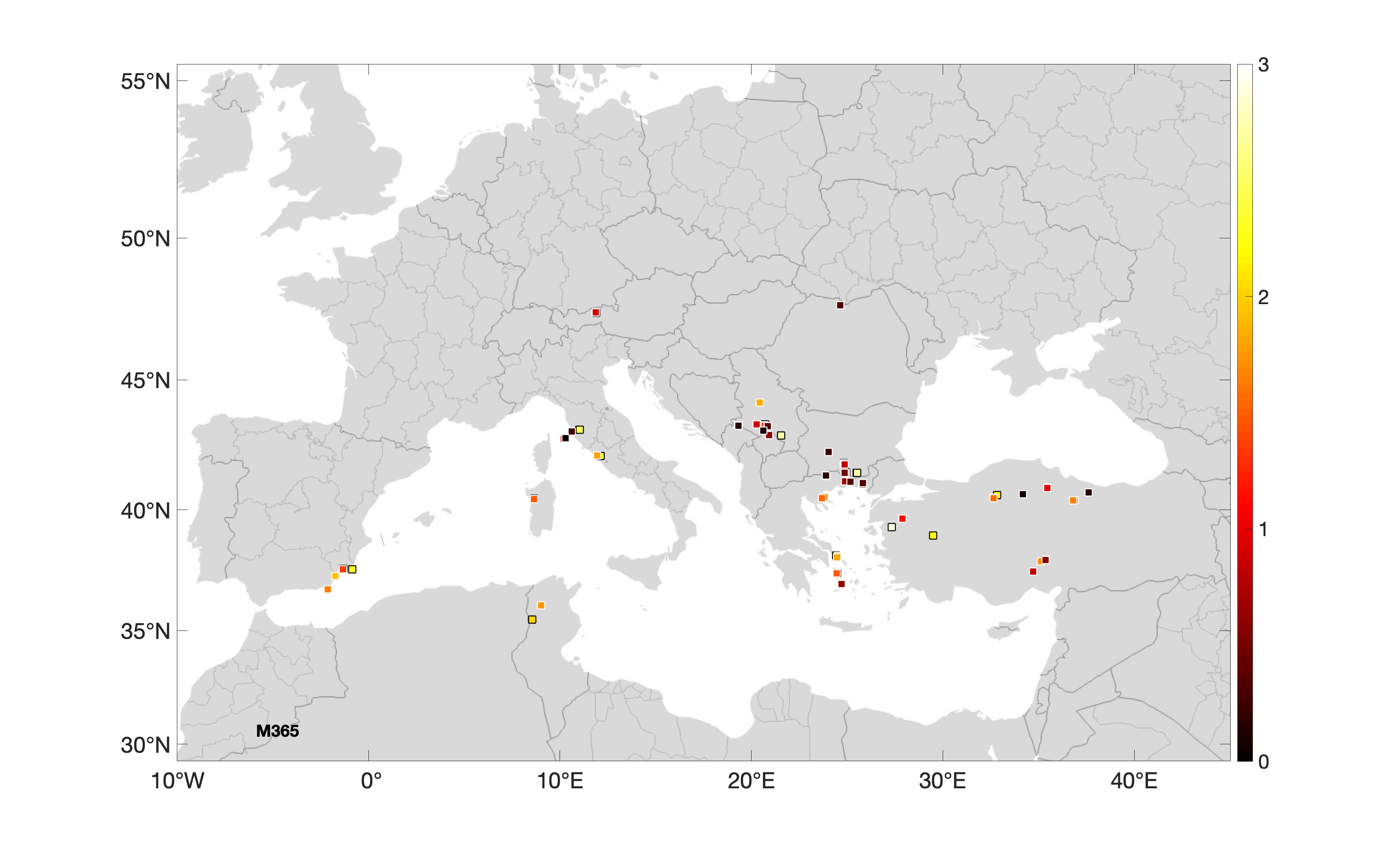

Supplement: Supplementary file 3 — Supplementary Material 3 [file 12520_2024_2106_MOESM3_ESM.zip › ESM3/png_hit maps/M365_map_jittered.png]

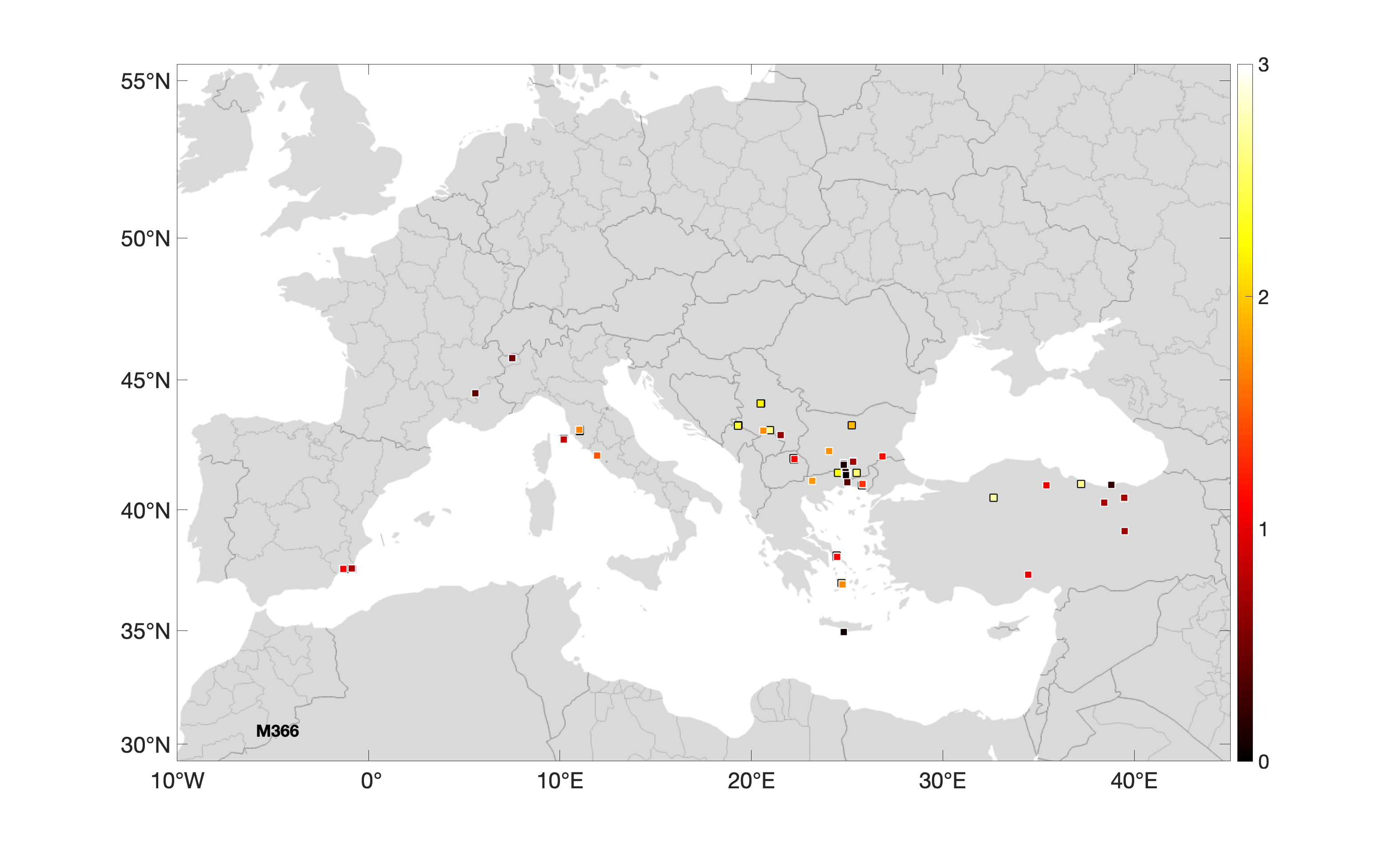

Supplement: Supplementary file 3 — Supplementary Material 3 [file 12520_2024_2106_MOESM3_ESM.zip › ESM3/png_hit maps/M366_map_jittered.png]

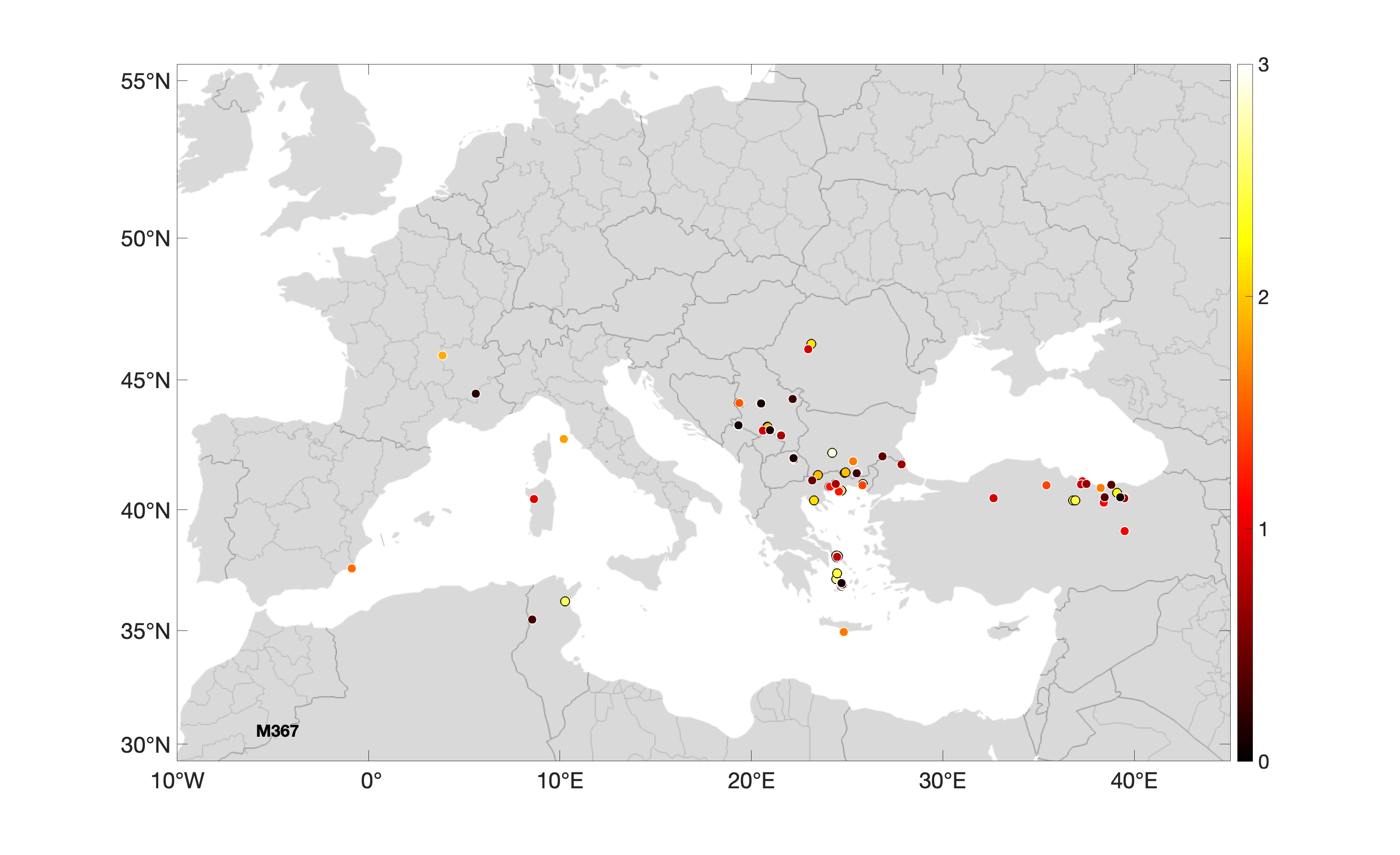

Supplement: Supplementary file 3 — Supplementary Material 3 [file 12520_2024_2106_MOESM3_ESM.zip › ESM3/png_hit maps/M367_map_jittered.png]

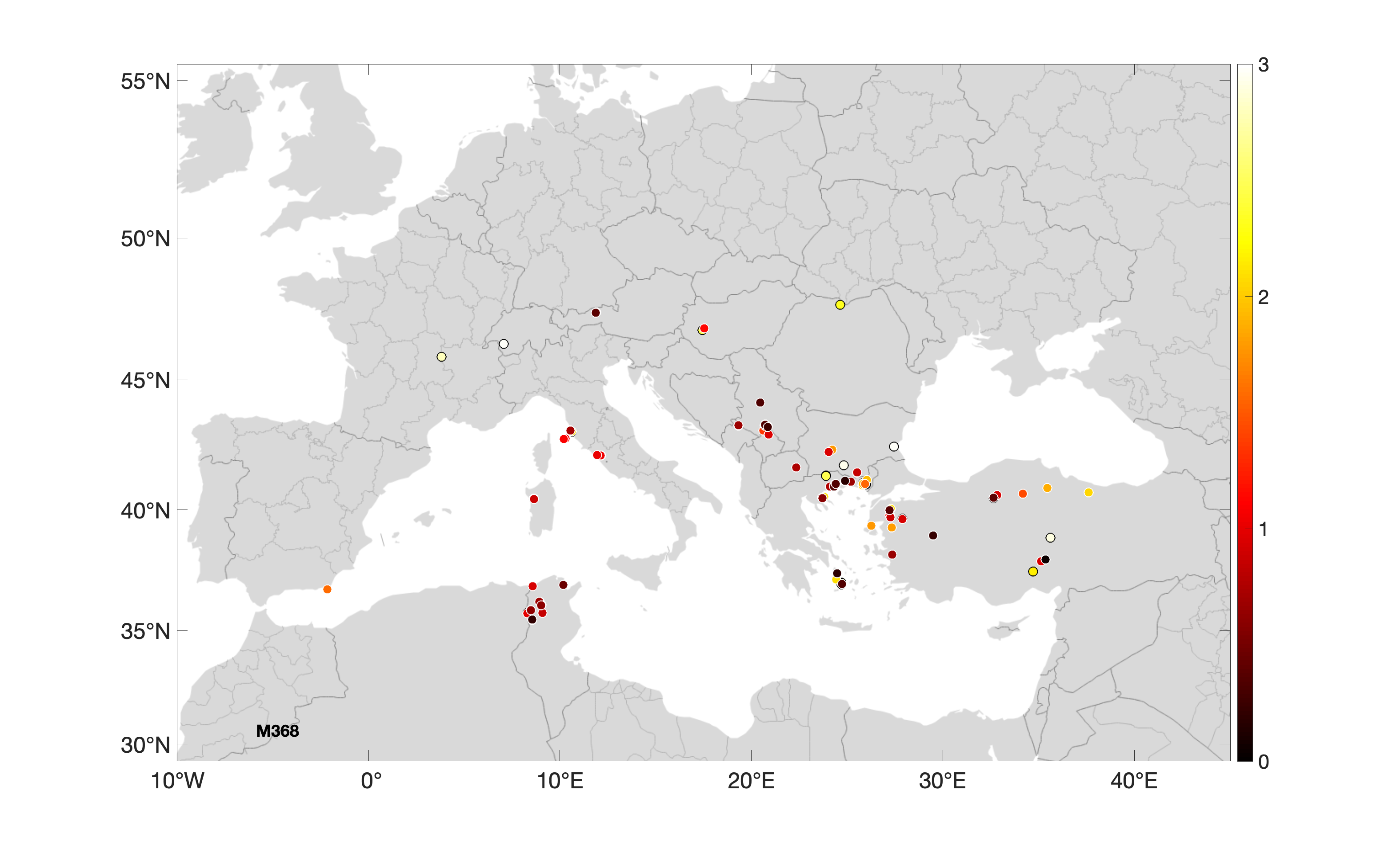

Supplement: Supplementary file 3 — Supplementary Material 3 [file 12520_2024_2106_MOESM3_ESM.zip › ESM3/png_hit maps/M368_map_jittered.png]

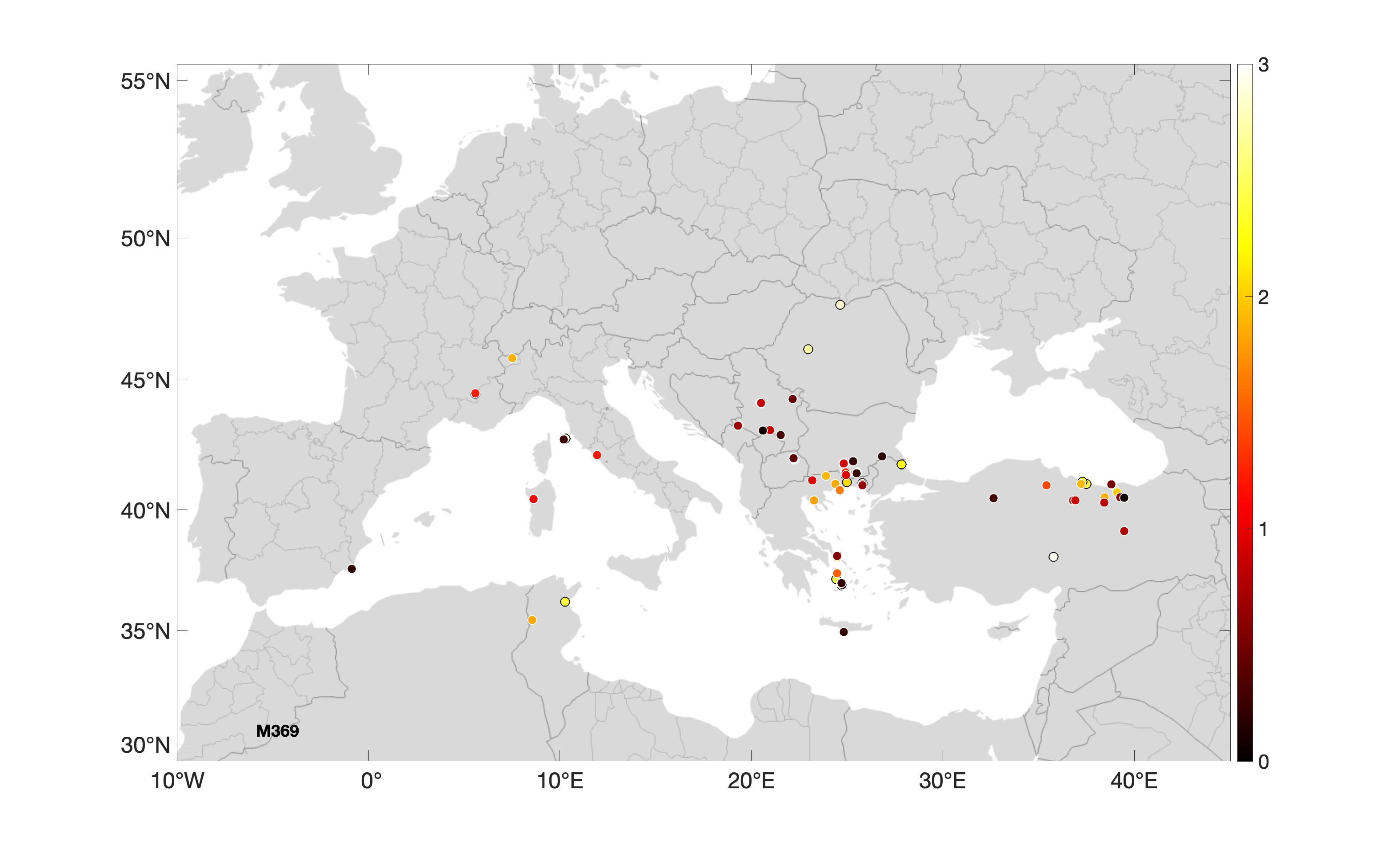

Supplement: Supplementary file 3 — Supplementary Material 3 [file 12520_2024_2106_MOESM3_ESM.zip › ESM3/png_hit maps/M369_map_jittered.png]

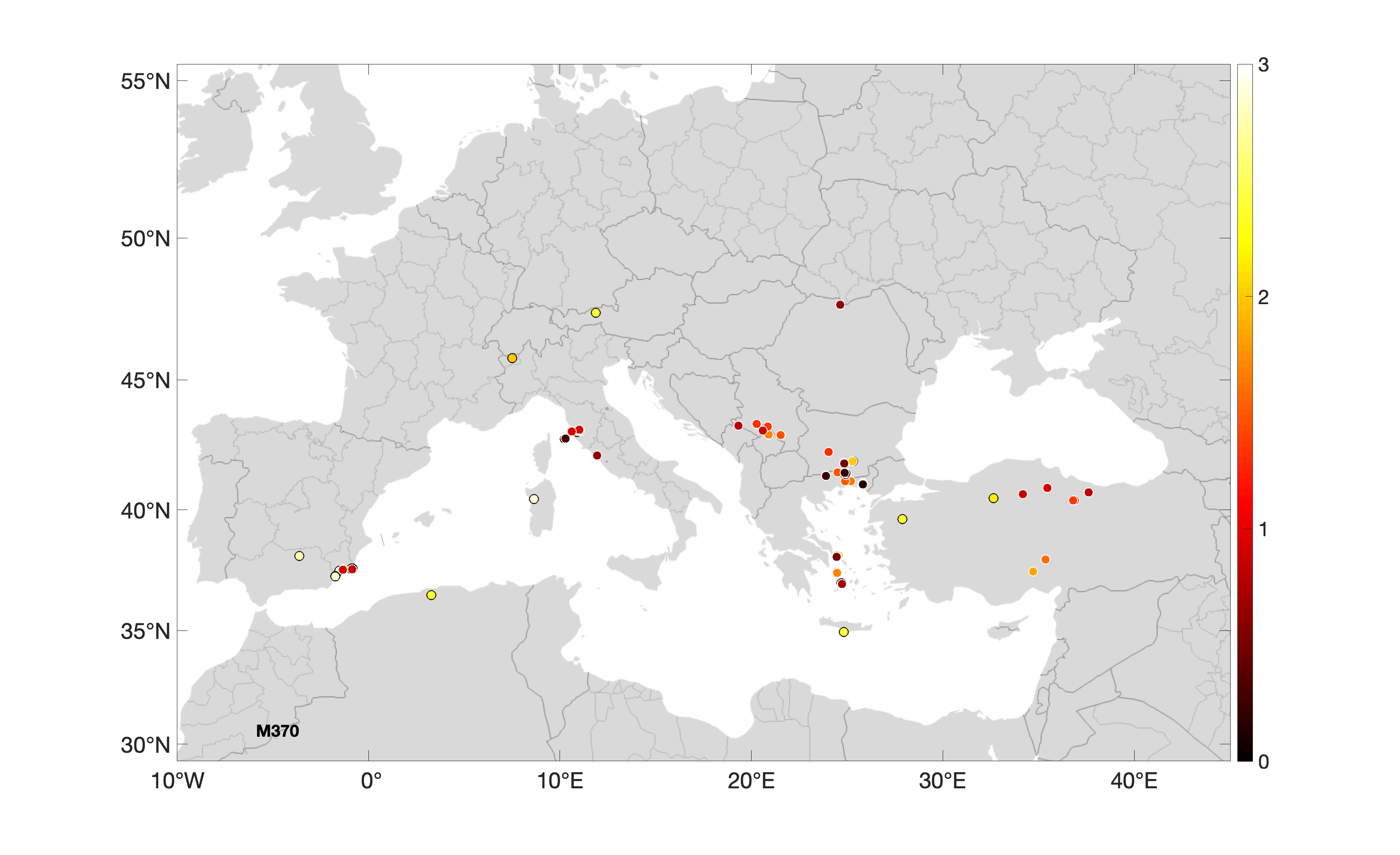

Supplement: Supplementary file 3 — Supplementary Material 3 [file 12520_2024_2106_MOESM3_ESM.zip › ESM3/png_hit maps/M370_map_jittered.png]

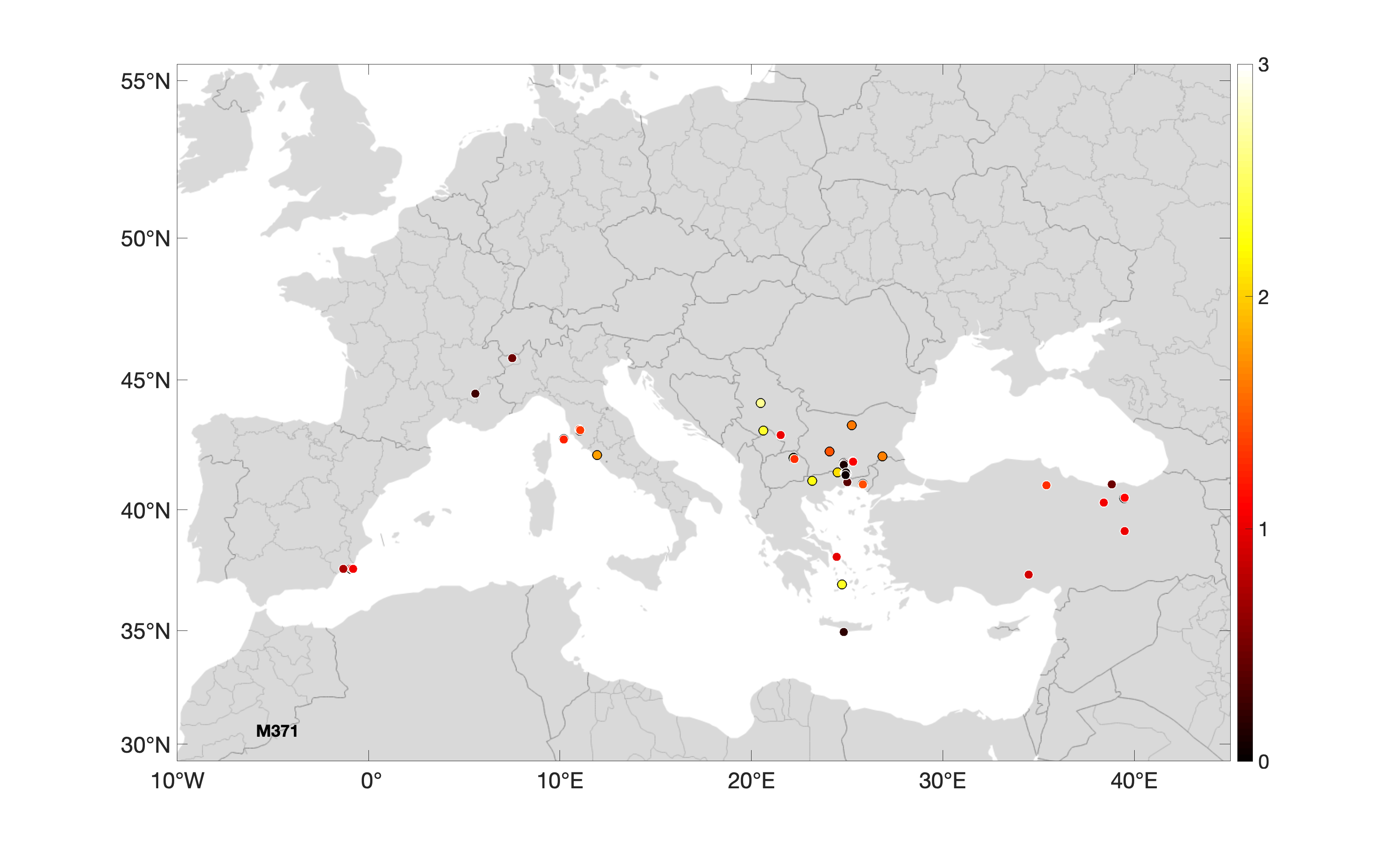

Supplement: Supplementary file 3 — Supplementary Material 3 [file 12520_2024_2106_MOESM3_ESM.zip › ESM3/png_hit maps/M371_map_jittered.png]

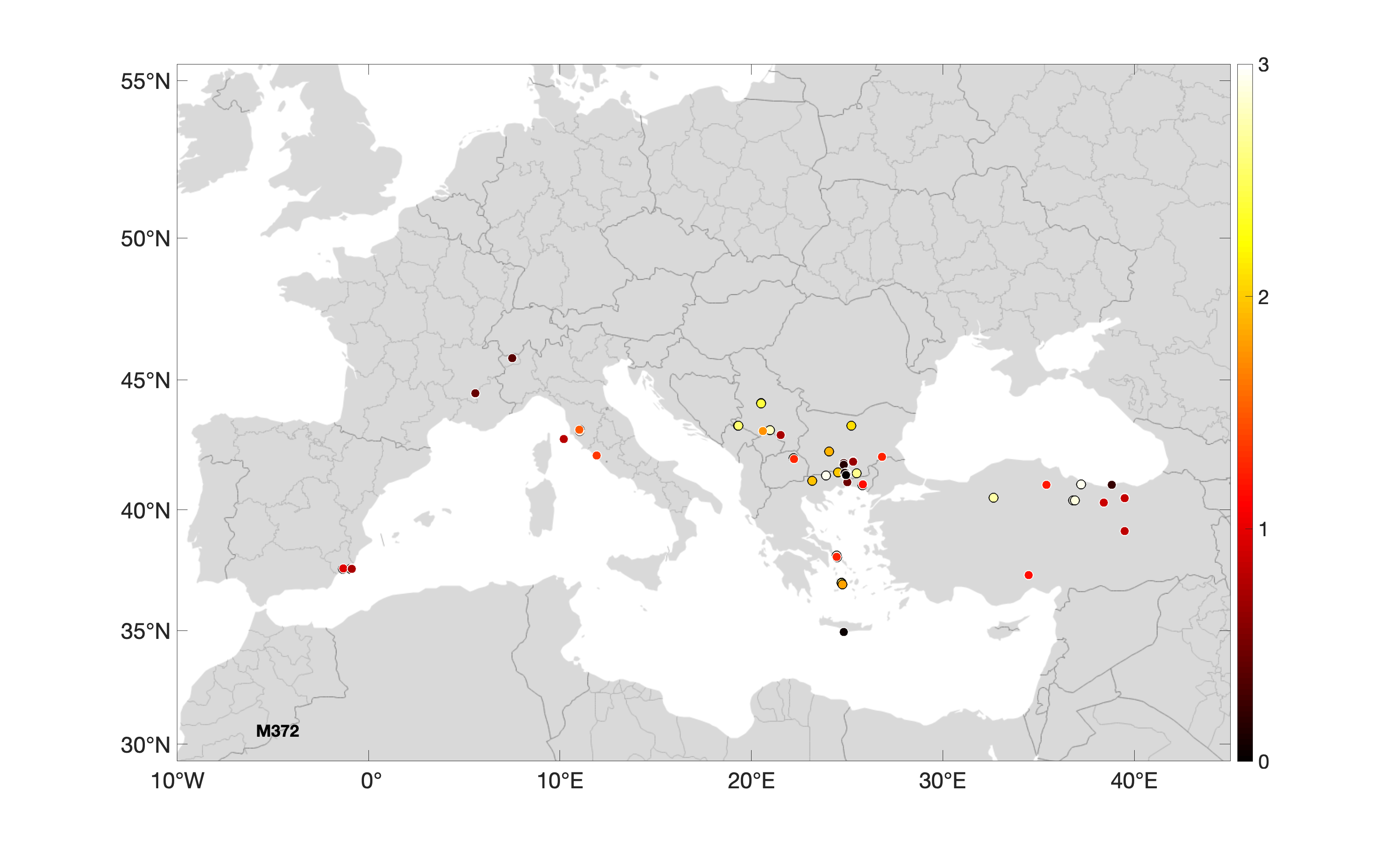

Supplement: Supplementary file 3 — Supplementary Material 3 [file 12520_2024_2106_MOESM3_ESM.zip › ESM3/png_hit maps/M372_map_jittered.png]

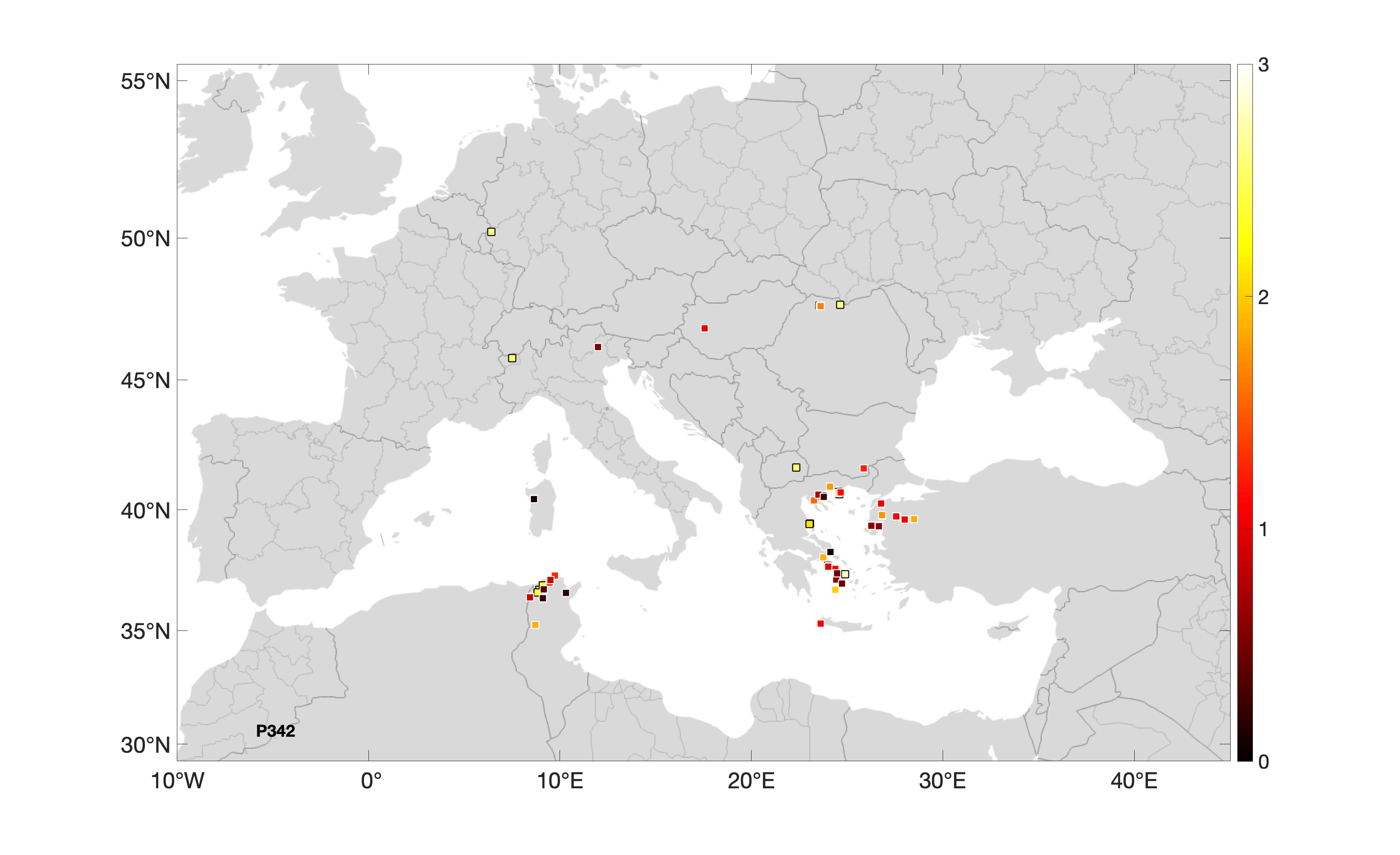

Supplement: Supplementary file 3 — Supplementary Material 3 [file 12520_2024_2106_MOESM3_ESM.zip › ESM3/png_hit maps/P342_map_jittered.png]

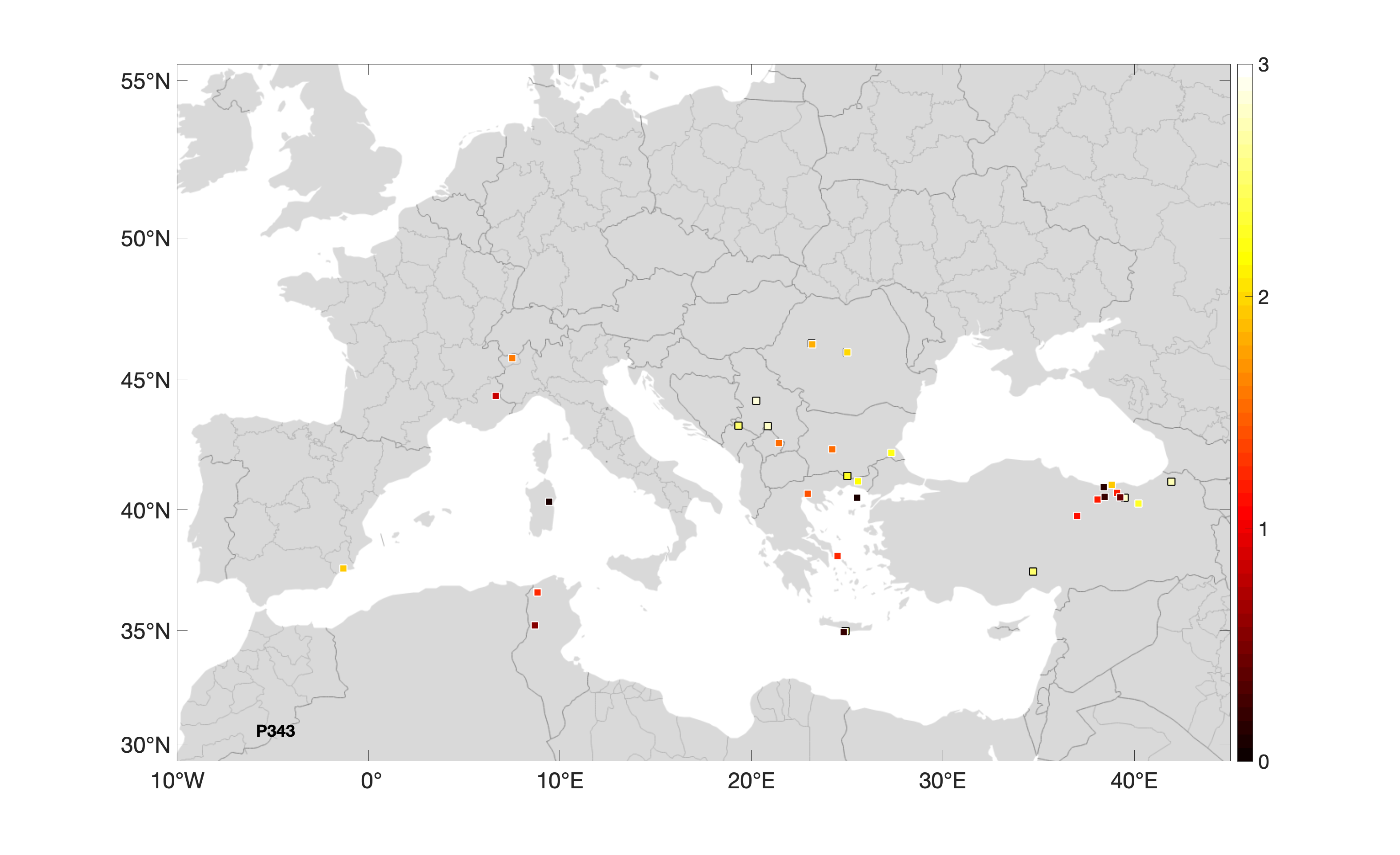

Supplement: Supplementary file 3 — Supplementary Material 3 [file 12520_2024_2106_MOESM3_ESM.zip › ESM3/png_hit maps/P343_map_jittered.png]

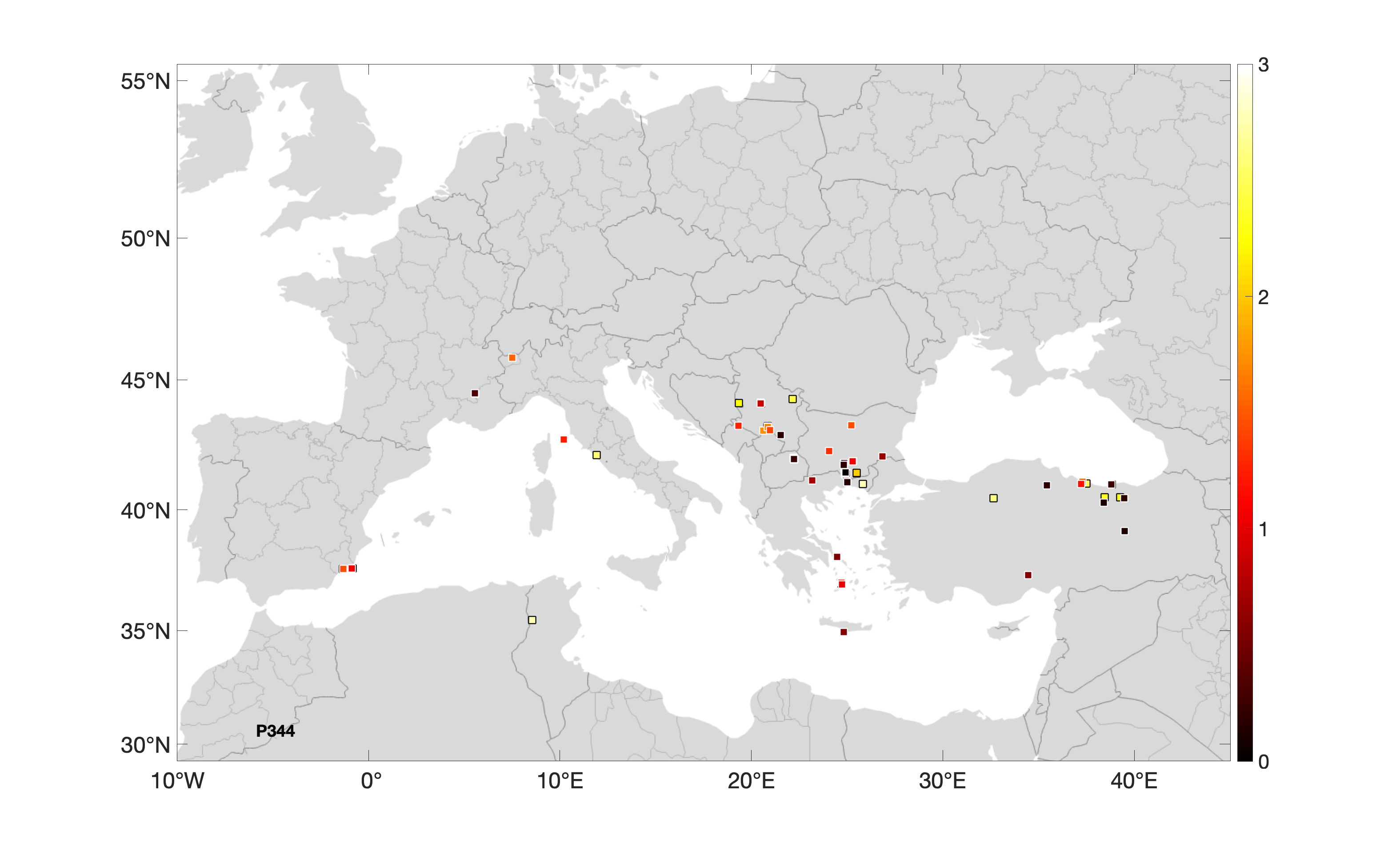

Supplement: Supplementary file 3 — Supplementary Material 3 [file 12520_2024_2106_MOESM3_ESM.zip › ESM3/png_hit maps/P344_map_jittered.png]

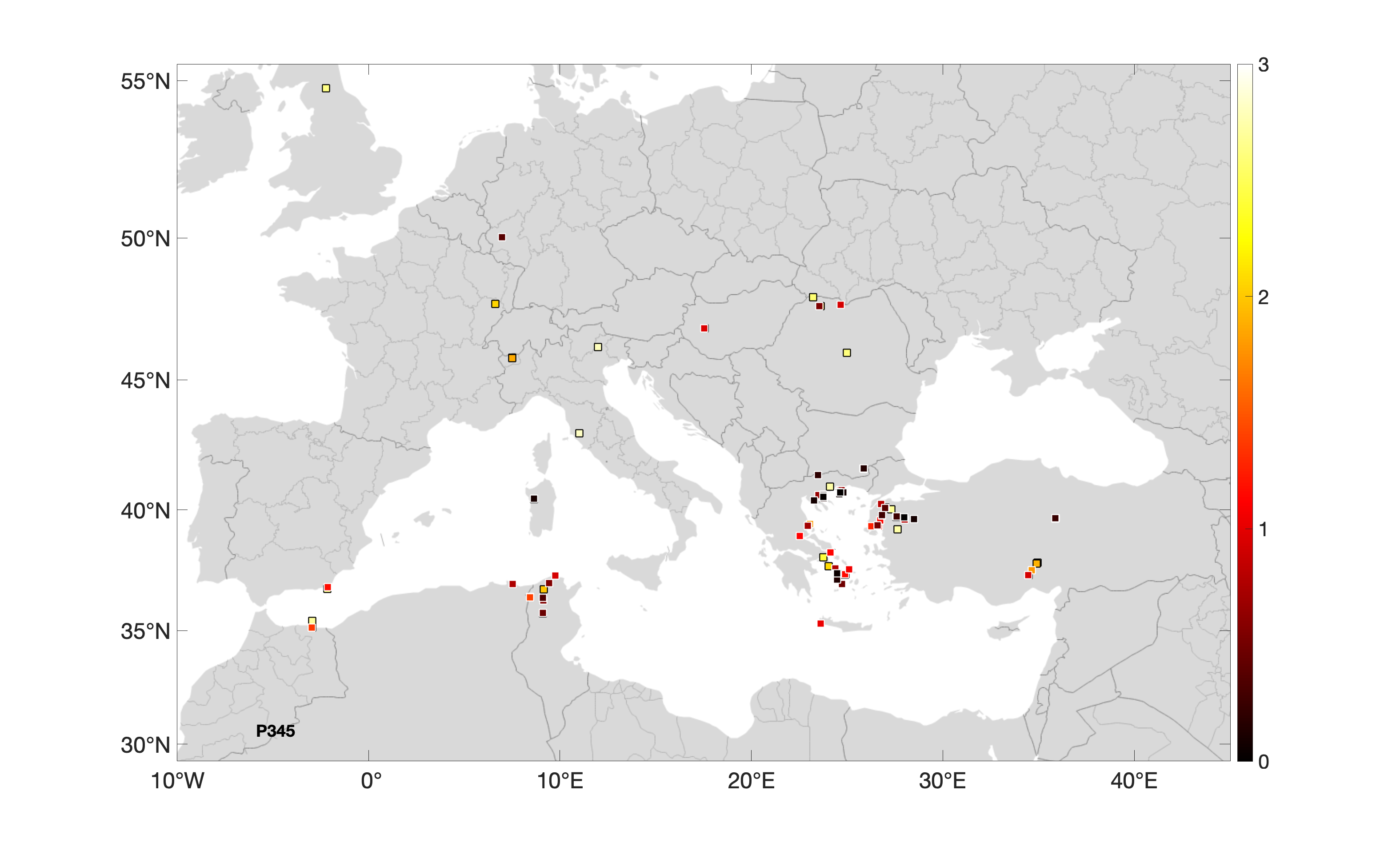

Supplement: Supplementary file 3 — Supplementary Material 3 [file 12520_2024_2106_MOESM3_ESM.zip › ESM3/png_hit maps/P345_map_jittered.png]

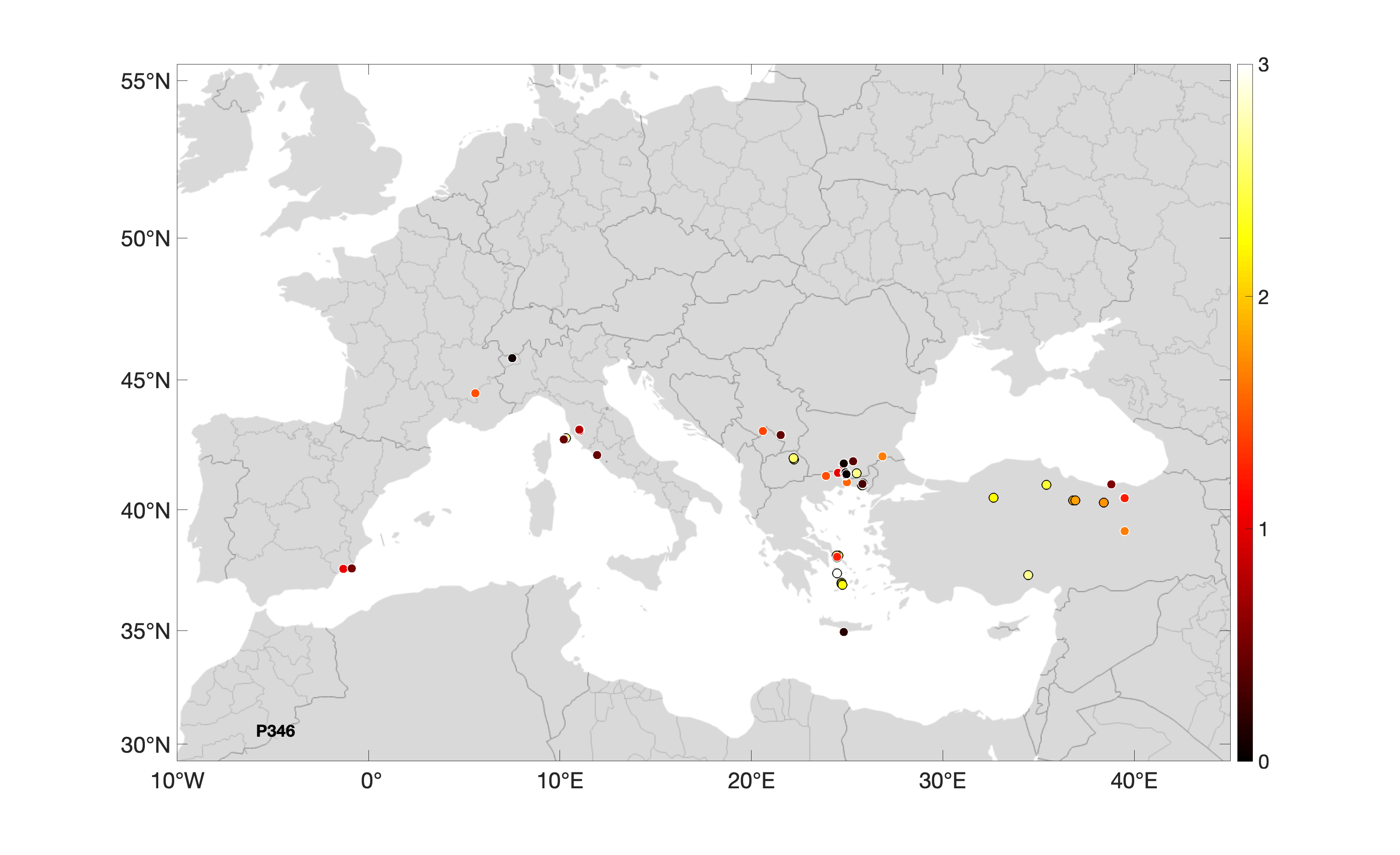

Supplement: Supplementary file 3 — Supplementary Material 3 [file 12520_2024_2106_MOESM3_ESM.zip › ESM3/png_hit maps/P346_map_jittered.png]

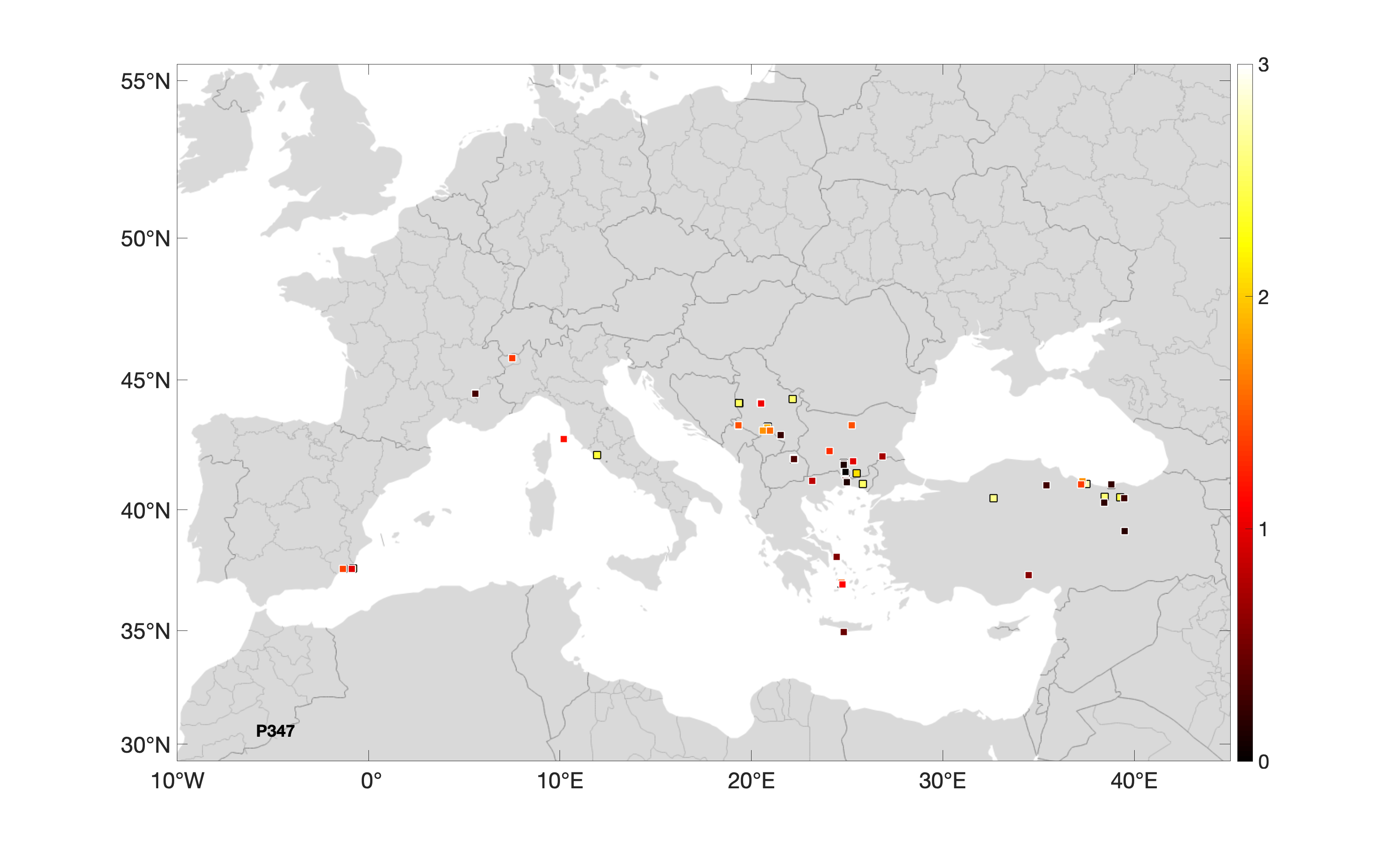

Supplement: Supplementary file 3 — Supplementary Material 3 [file 12520_2024_2106_MOESM3_ESM.zip › ESM3/png_hit maps/P347_map_jittered.png]

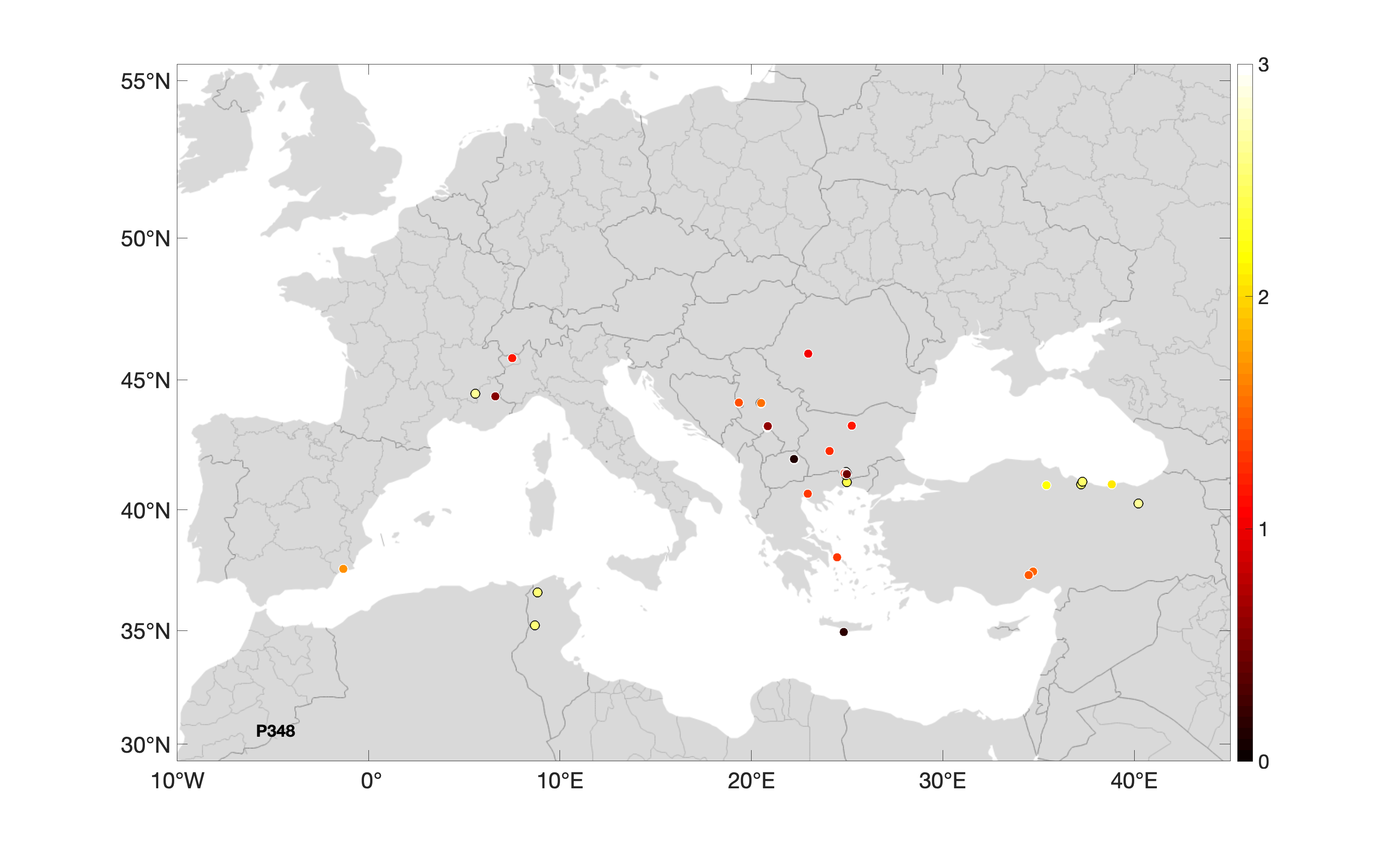

Supplement: Supplementary file 3 — Supplementary Material 3 [file 12520_2024_2106_MOESM3_ESM.zip › ESM3/png_hit maps/P348_map_jittered.png]

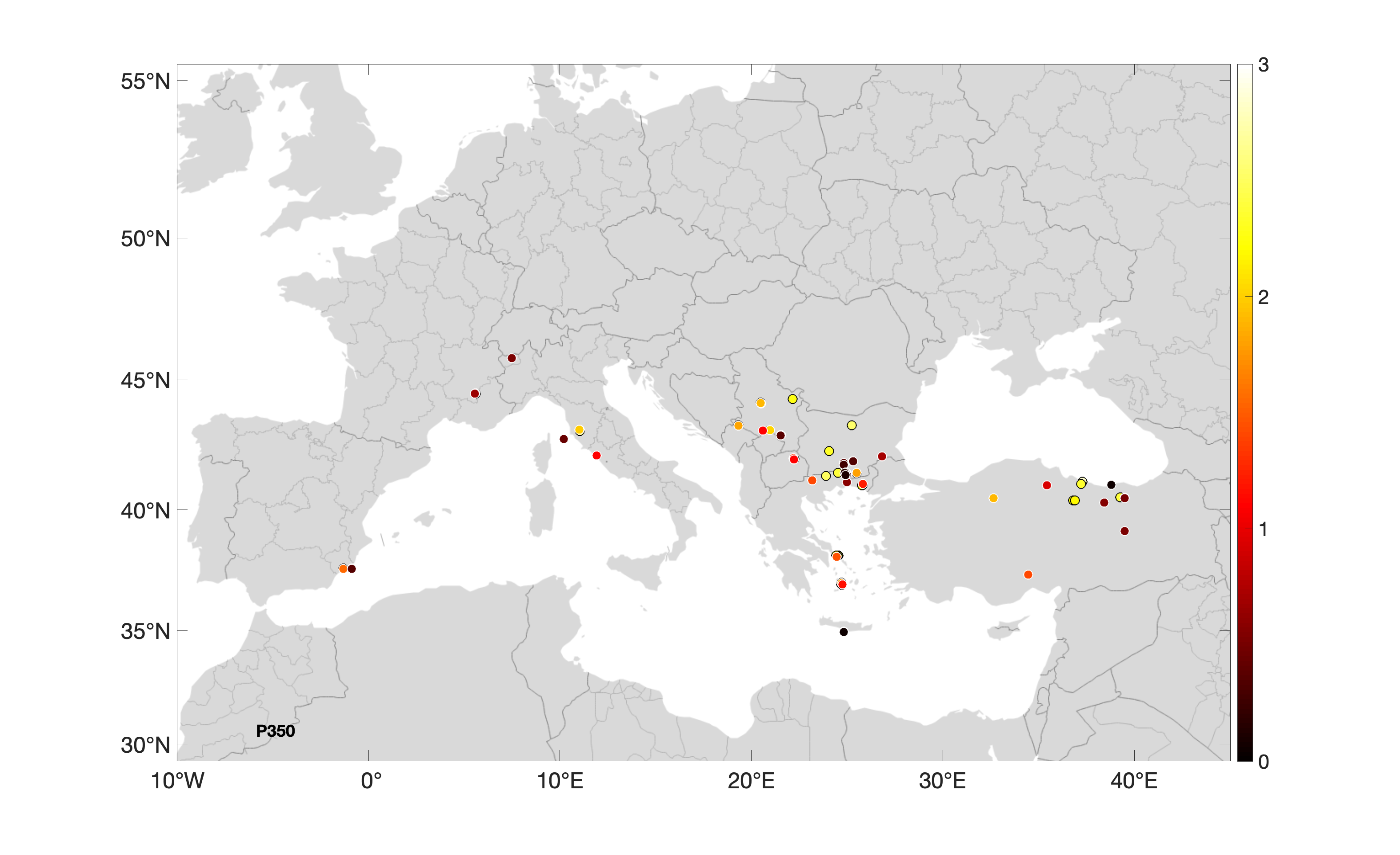

Supplement: Supplementary file 3 — Supplementary Material 3 [file 12520_2024_2106_MOESM3_ESM.zip › ESM3/png_hit maps/P350_map_jittered.png]

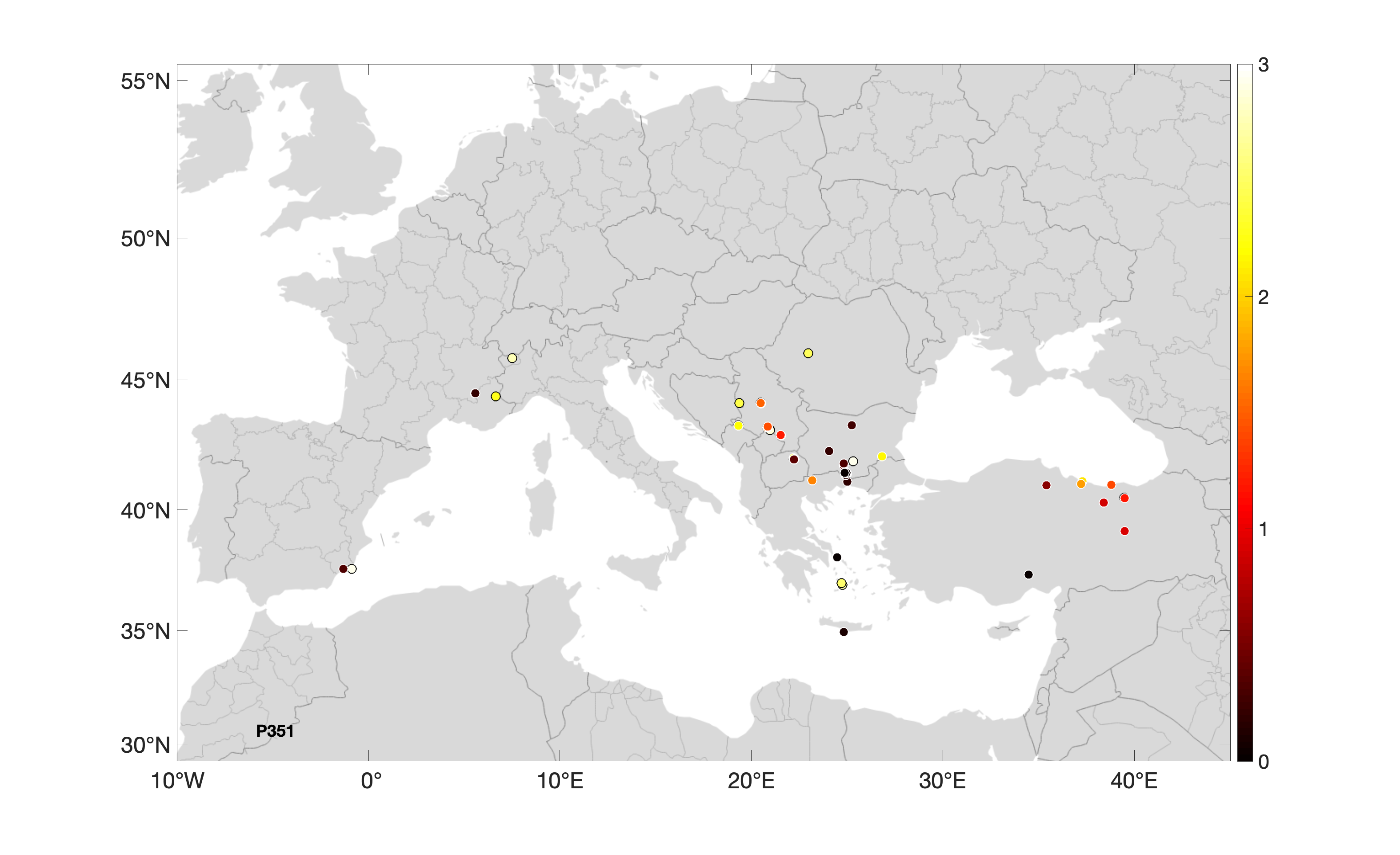

Supplement: Supplementary file 3 — Supplementary Material 3 [file 12520_2024_2106_MOESM3_ESM.zip › ESM3/png_hit maps/P351_map_jittered.png]

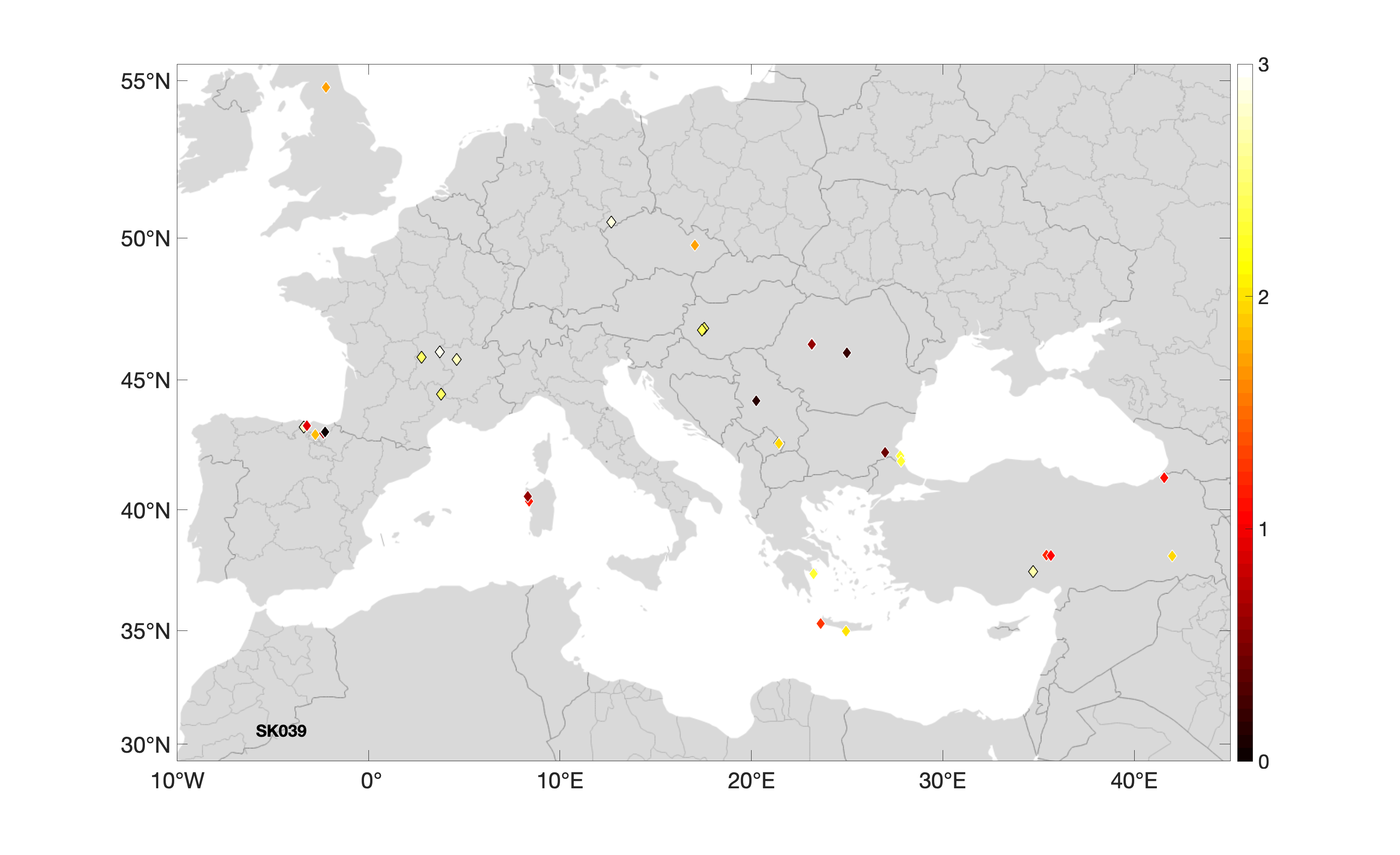

Supplement: Supplementary file 3 — Supplementary Material 3 [file 12520_2024_2106_MOESM3_ESM.zip › ESM3/png_hit maps/SK039_map_jittered.png]

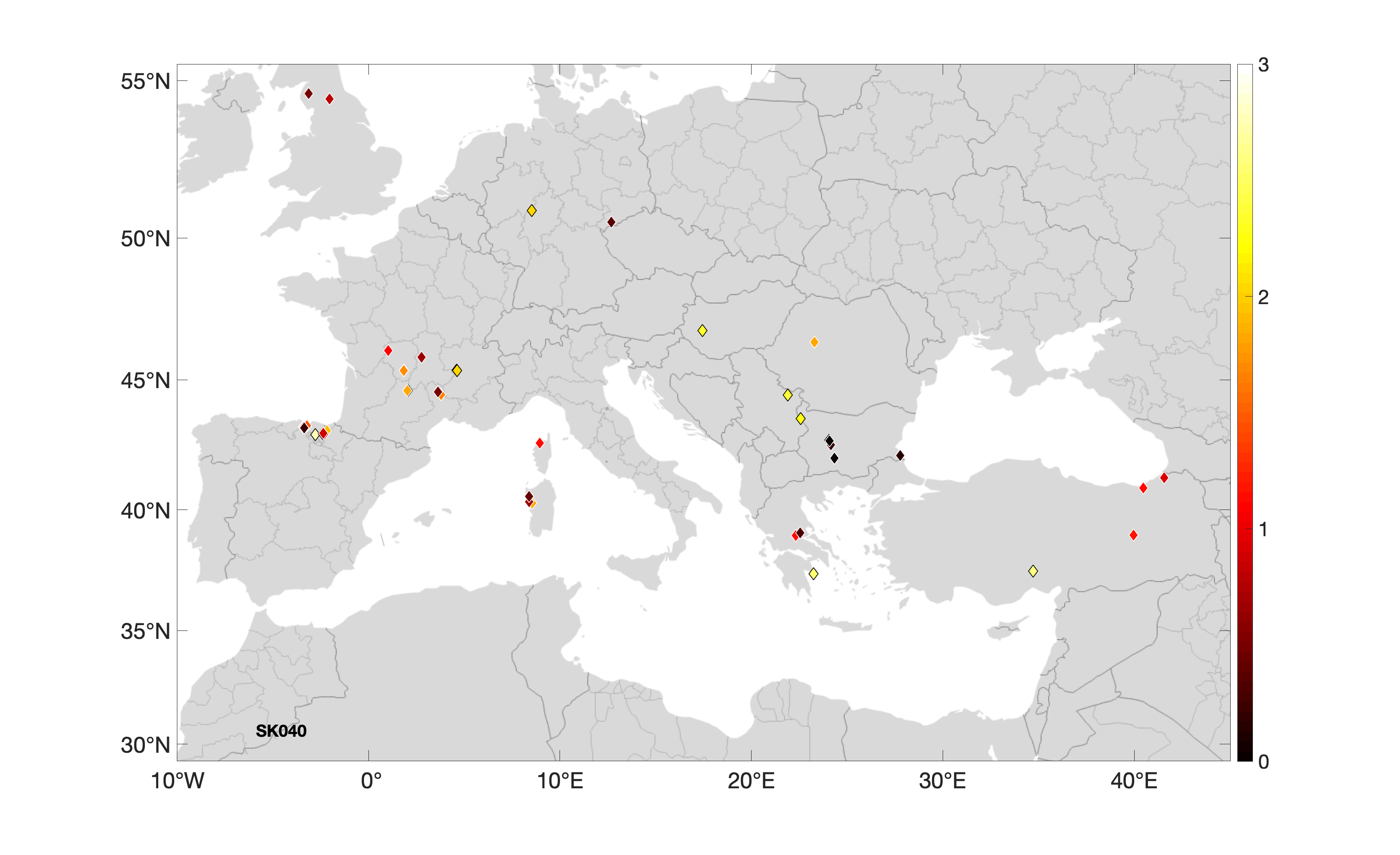

Supplement: Supplementary file 3 — Supplementary Material 3 [file 12520_2024_2106_MOESM3_ESM.zip › ESM3/png_hit maps/SK040_map_jittered.png]

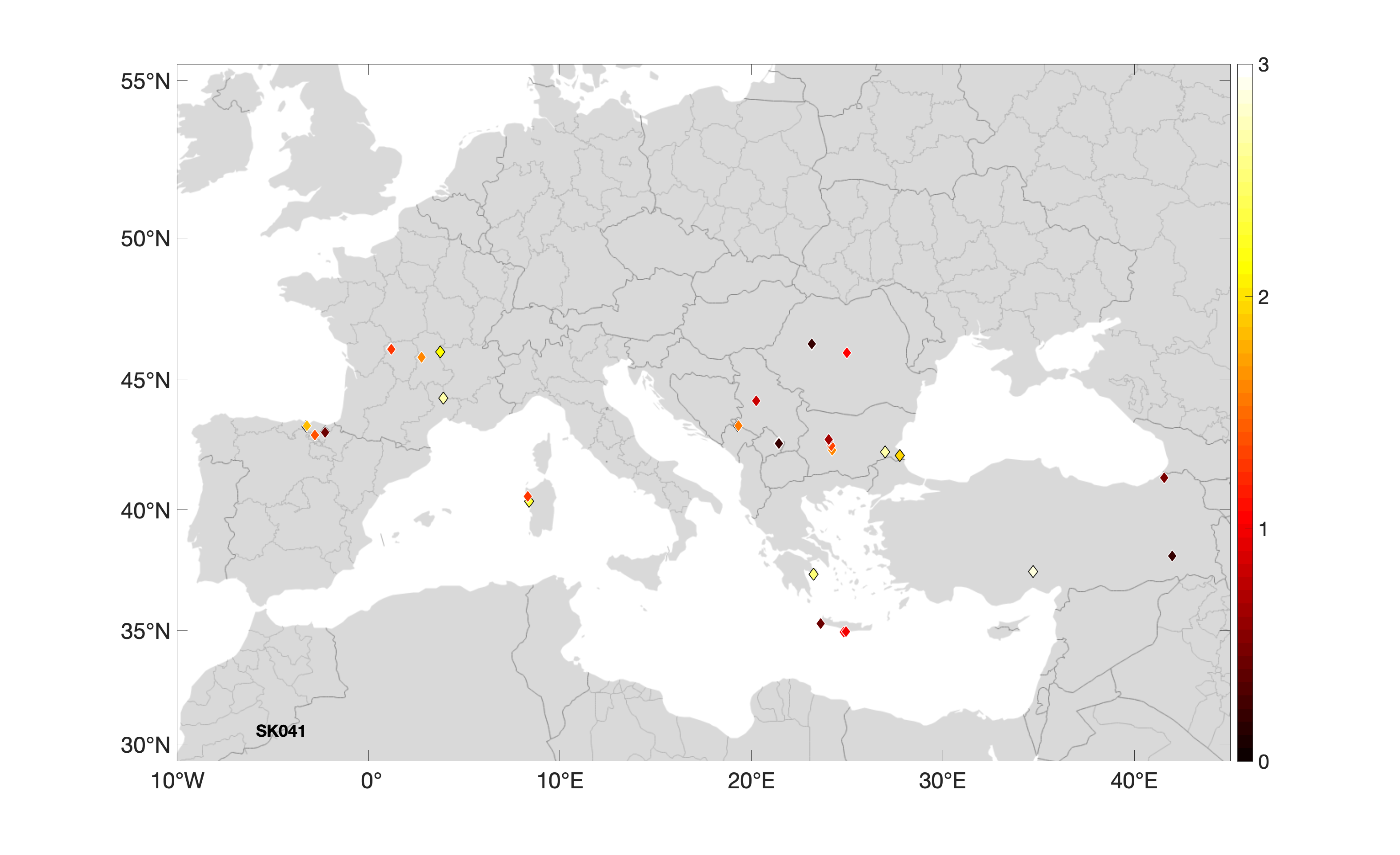

Supplement: Supplementary file 3 — Supplementary Material 3 [file 12520_2024_2106_MOESM3_ESM.zip › ESM3/png_hit maps/SK041_map_jittered.png]

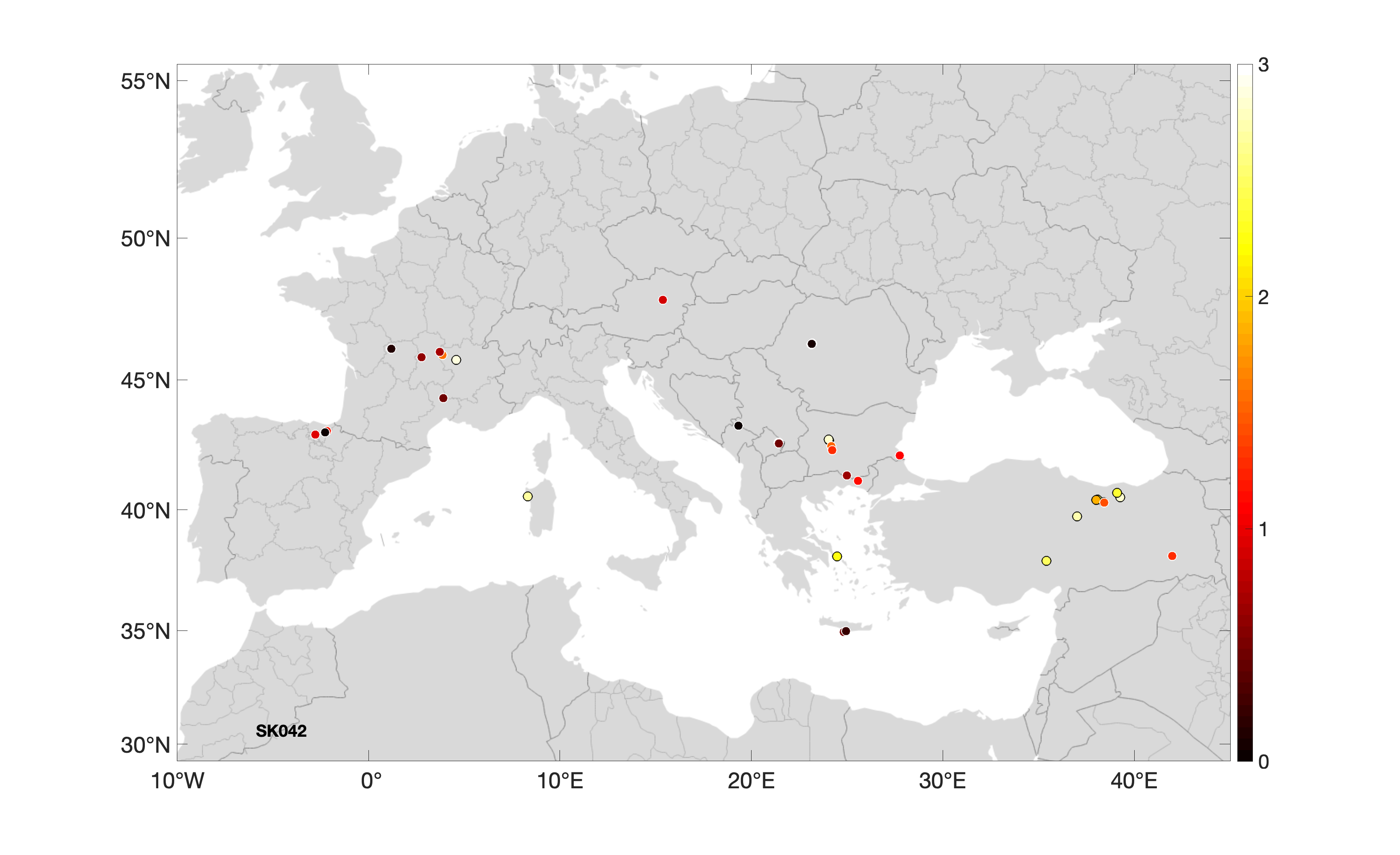

Supplement: Supplementary file 3 — Supplementary Material 3 [file 12520_2024_2106_MOESM3_ESM.zip › ESM3/png_hit maps/SK042_map_jittered.png]

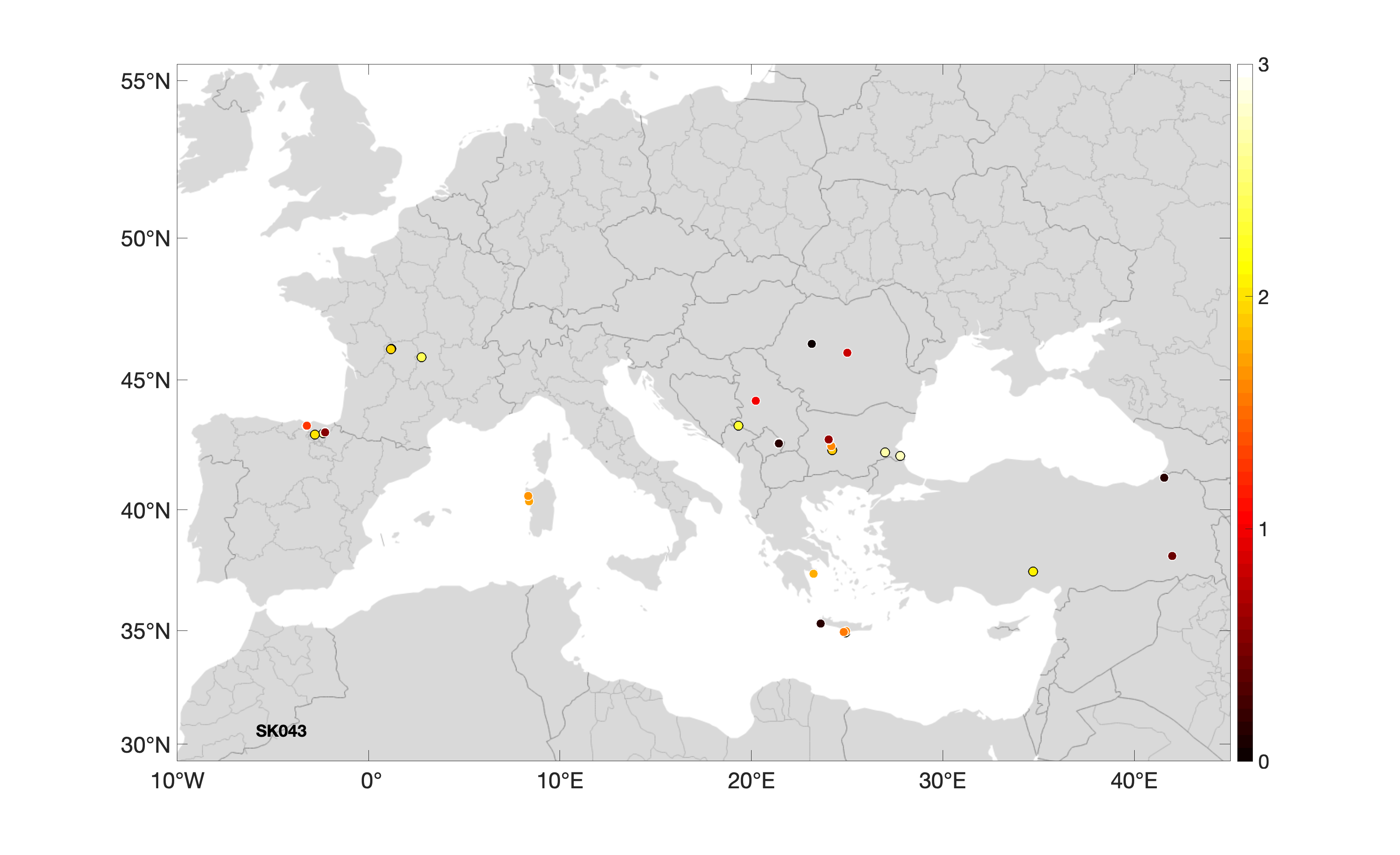

Supplement: Supplementary file 3 — Supplementary Material 3 [file 12520_2024_2106_MOESM3_ESM.zip › ESM3/png_hit maps/SK043_map_jittered.png]

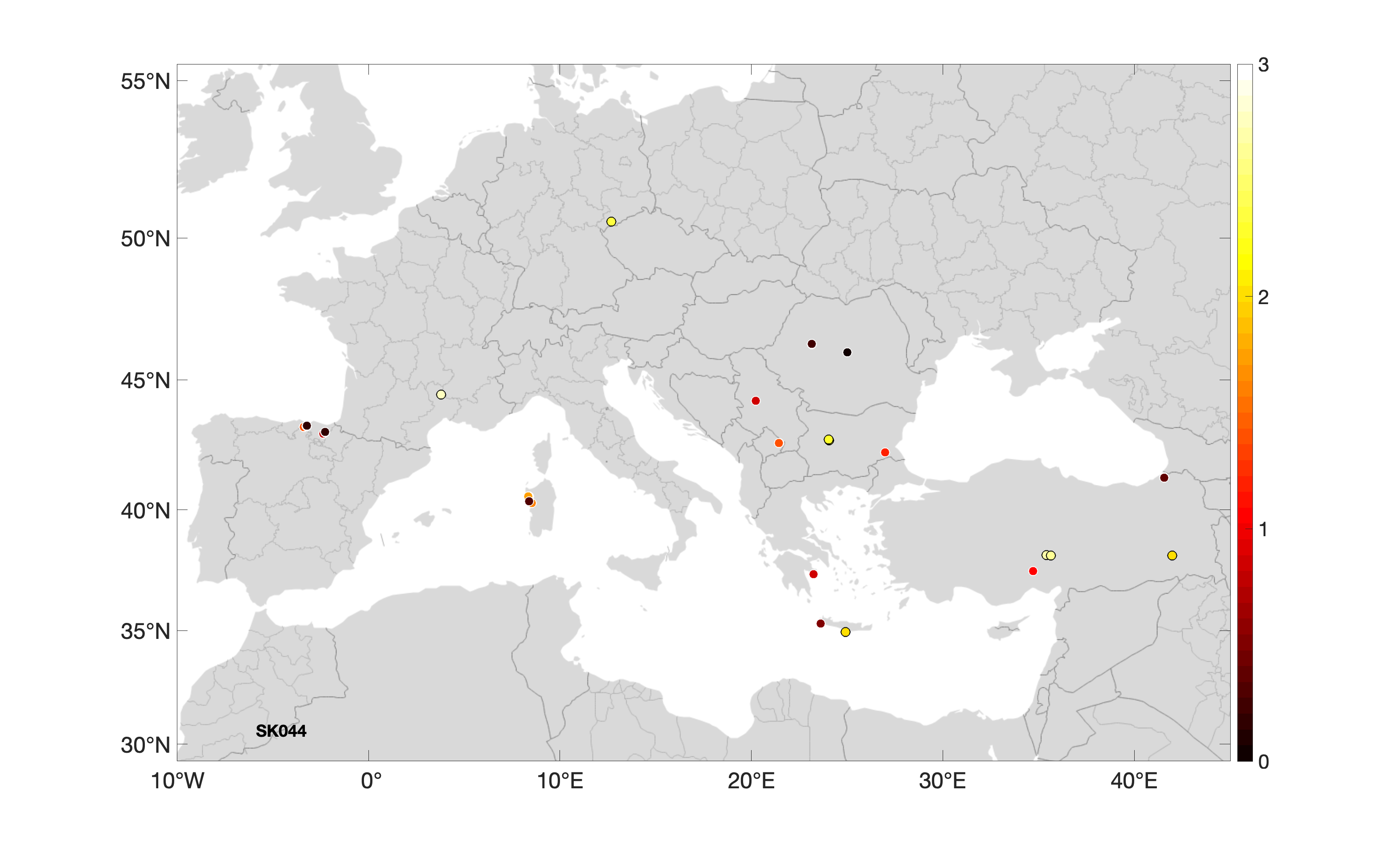

Supplement: Supplementary file 3 — Supplementary Material 3 [file 12520_2024_2106_MOESM3_ESM.zip › ESM3/png_hit maps/SK044_map_jittered.png]

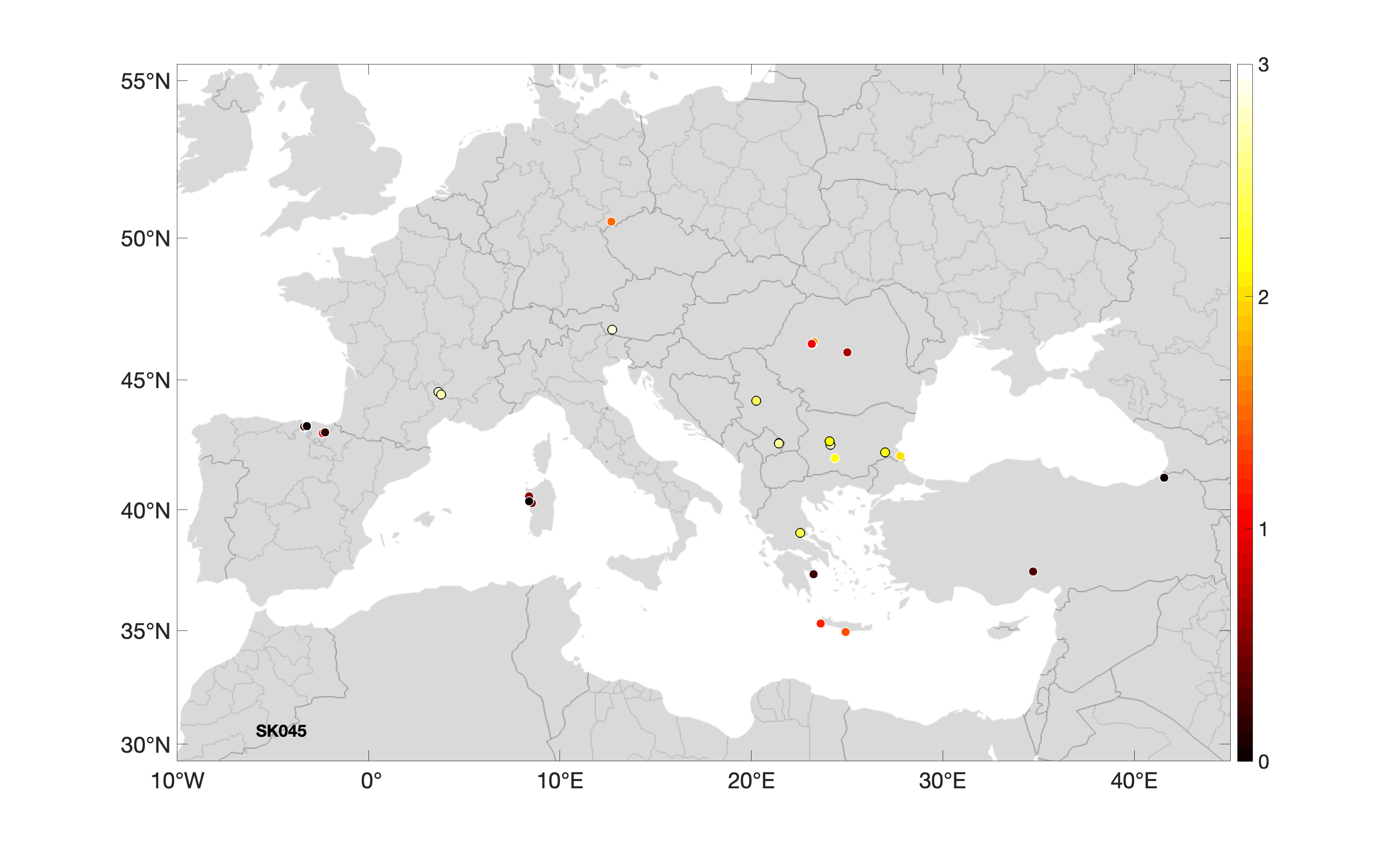

Supplement: Supplementary file 3 — Supplementary Material 3 [file 12520_2024_2106_MOESM3_ESM.zip › ESM3/png_hit maps/SK045_map_jittered.png]

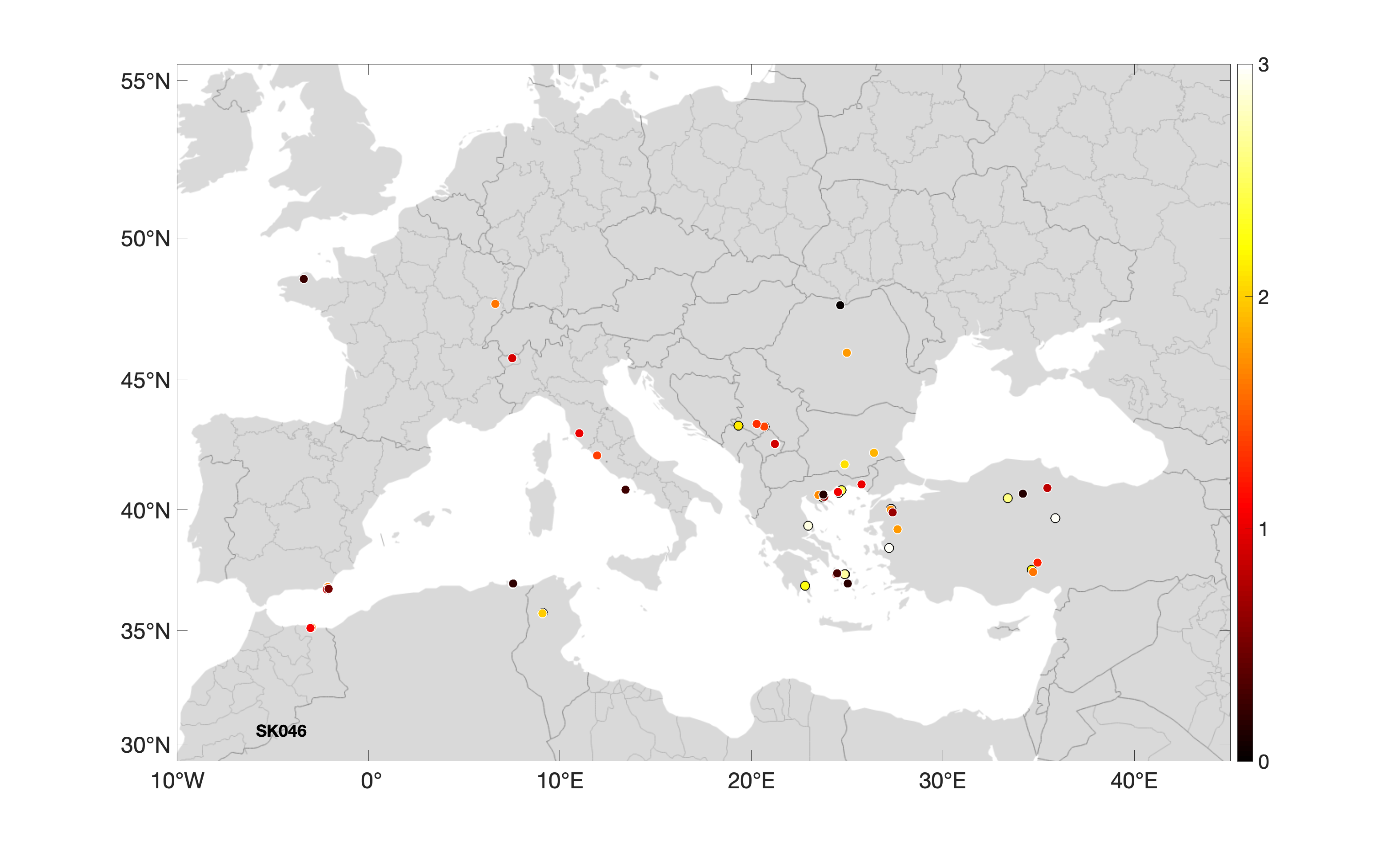

Supplement: Supplementary file 3 — Supplementary Material 3 [file 12520_2024_2106_MOESM3_ESM.zip › ESM3/png_hit maps/SK046_map_jittered.png]

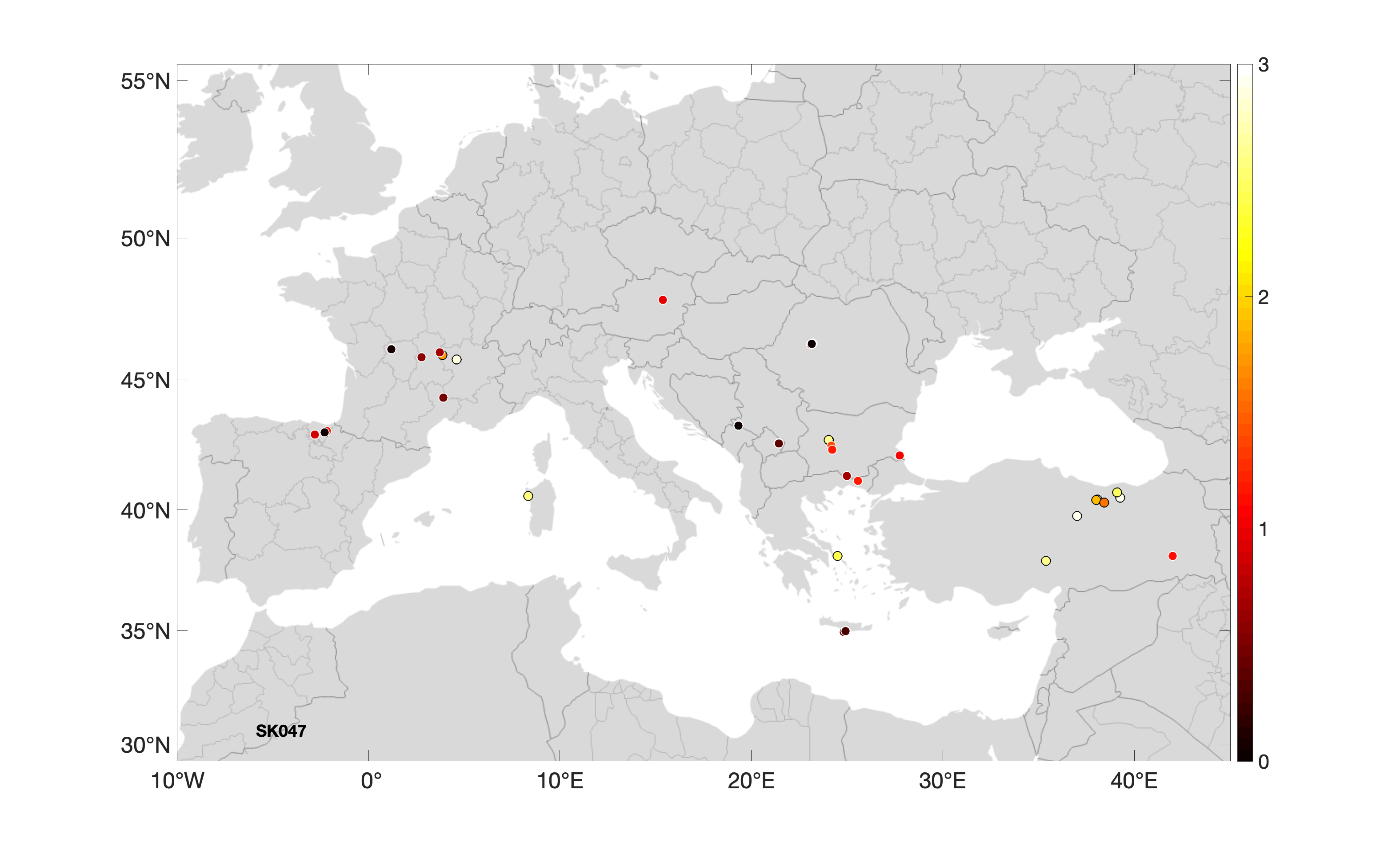

Supplement: Supplementary file 3 — Supplementary Material 3 [file 12520_2024_2106_MOESM3_ESM.zip › ESM3/png_hit maps/SK047_map_jittered.png]

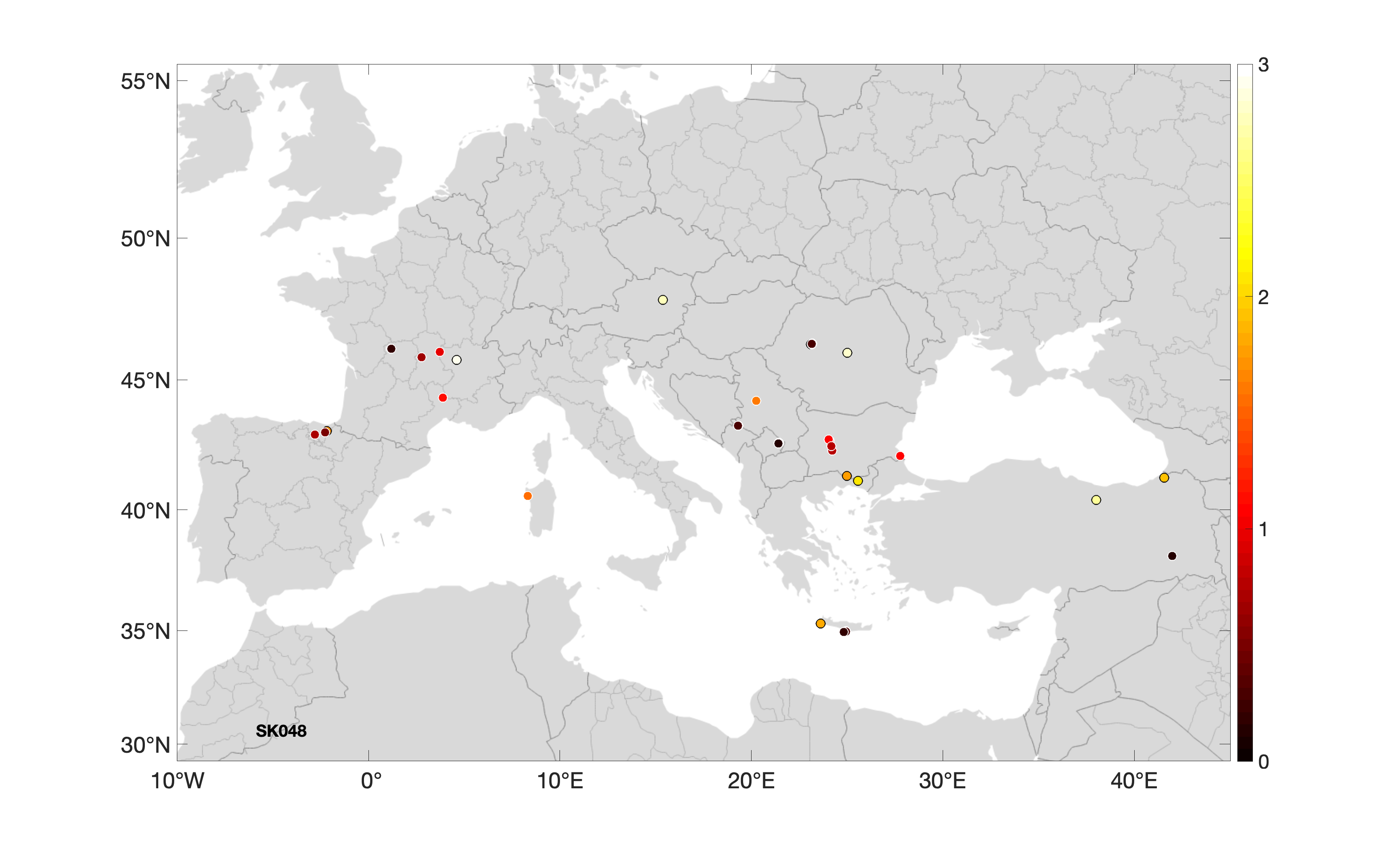

Supplement: Supplementary file 3 — Supplementary Material 3 [file 12520_2024_2106_MOESM3_ESM.zip › ESM3/png_hit maps/SK048_map_jittered.png]

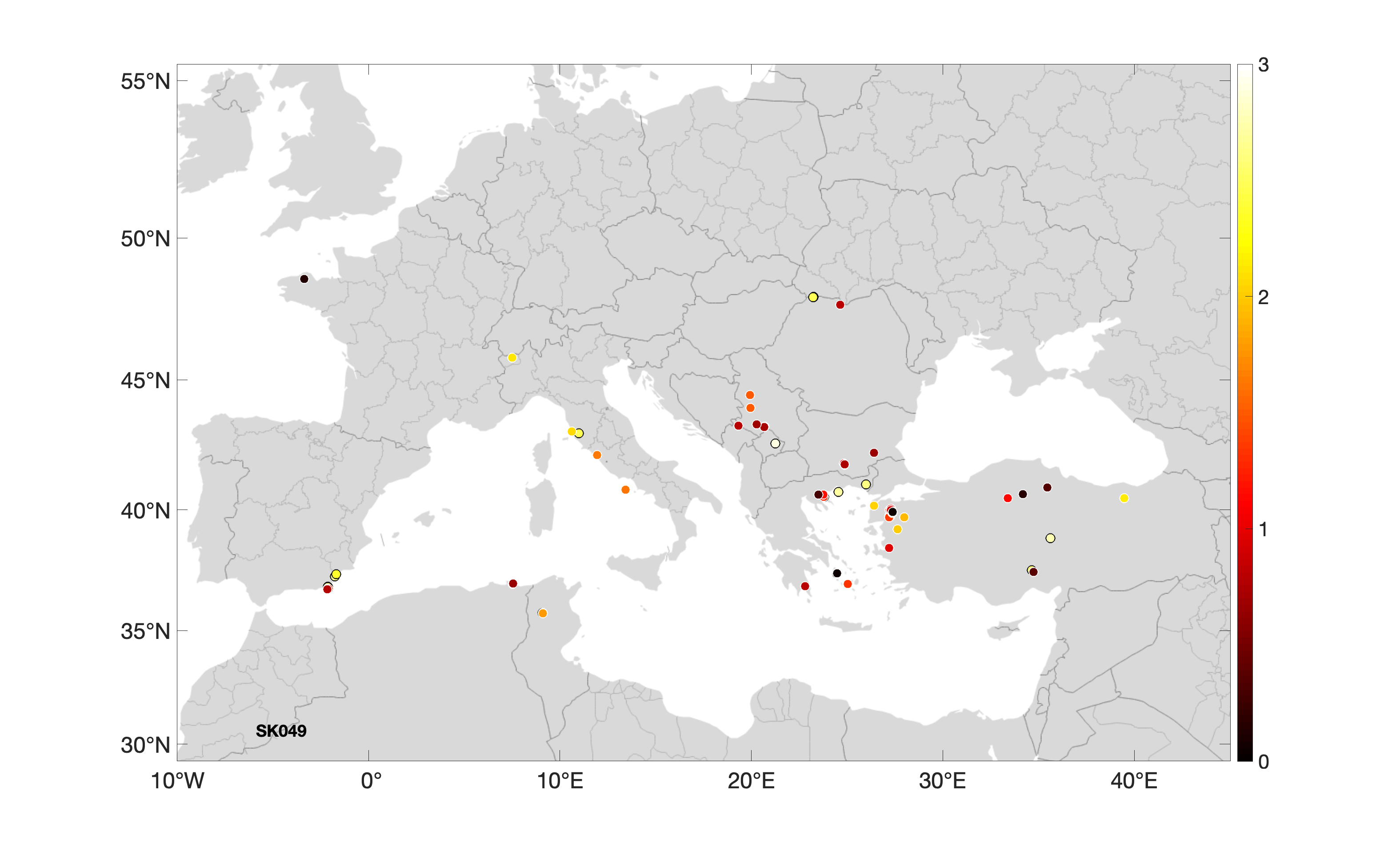

Supplement: Supplementary file 3 — Supplementary Material 3 [file 12520_2024_2106_MOESM3_ESM.zip › ESM3/png_hit maps/SK049_map_jittered.png]

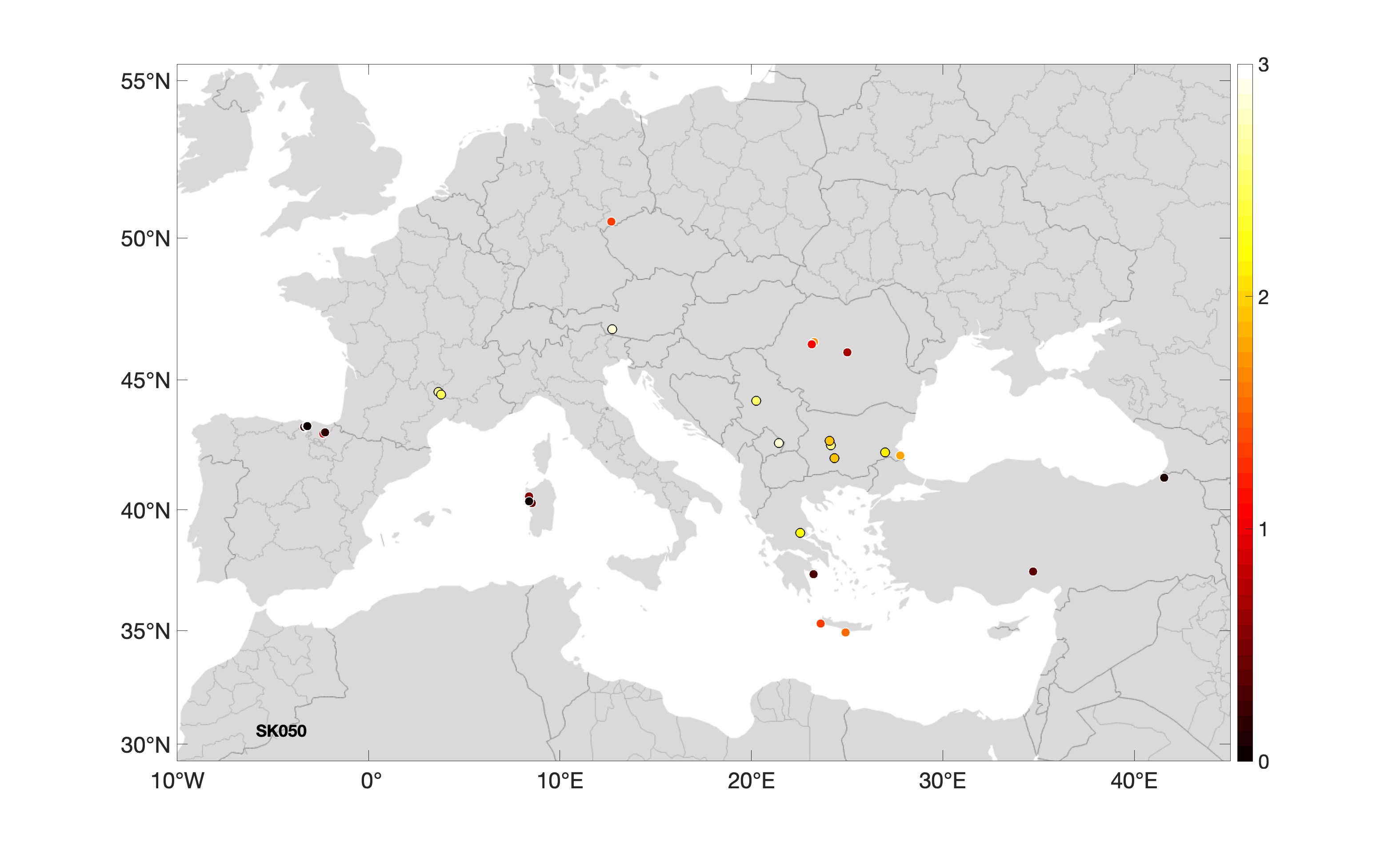

Supplement: Supplementary file 3 — Supplementary Material 3 [file 12520_2024_2106_MOESM3_ESM.zip › ESM3/png_hit maps/SK050_map_jittered.png]

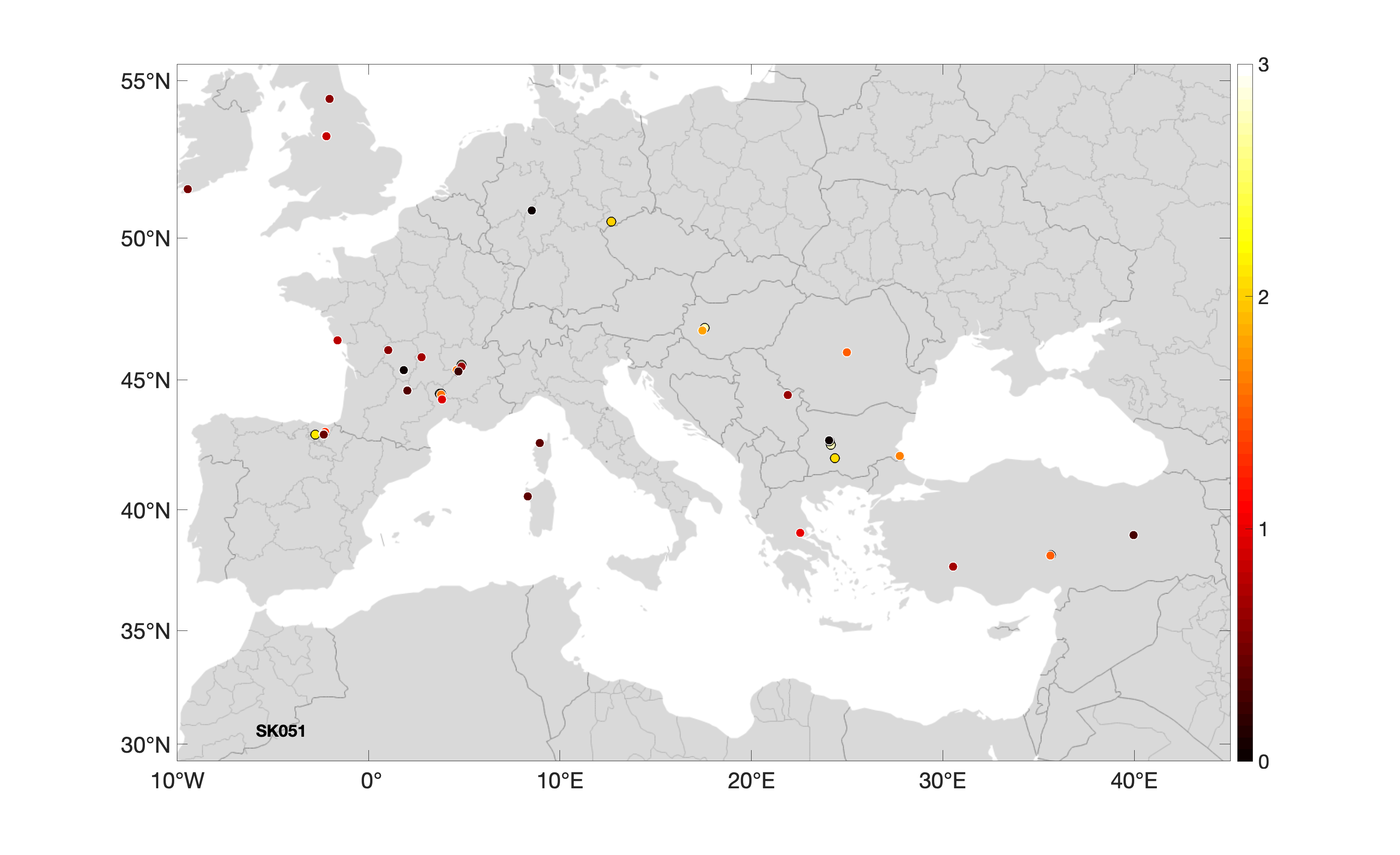

Supplement: Supplementary file 3 — Supplementary Material 3 [file 12520_2024_2106_MOESM3_ESM.zip › ESM3/png_hit maps/SK051_map_jittered.png]

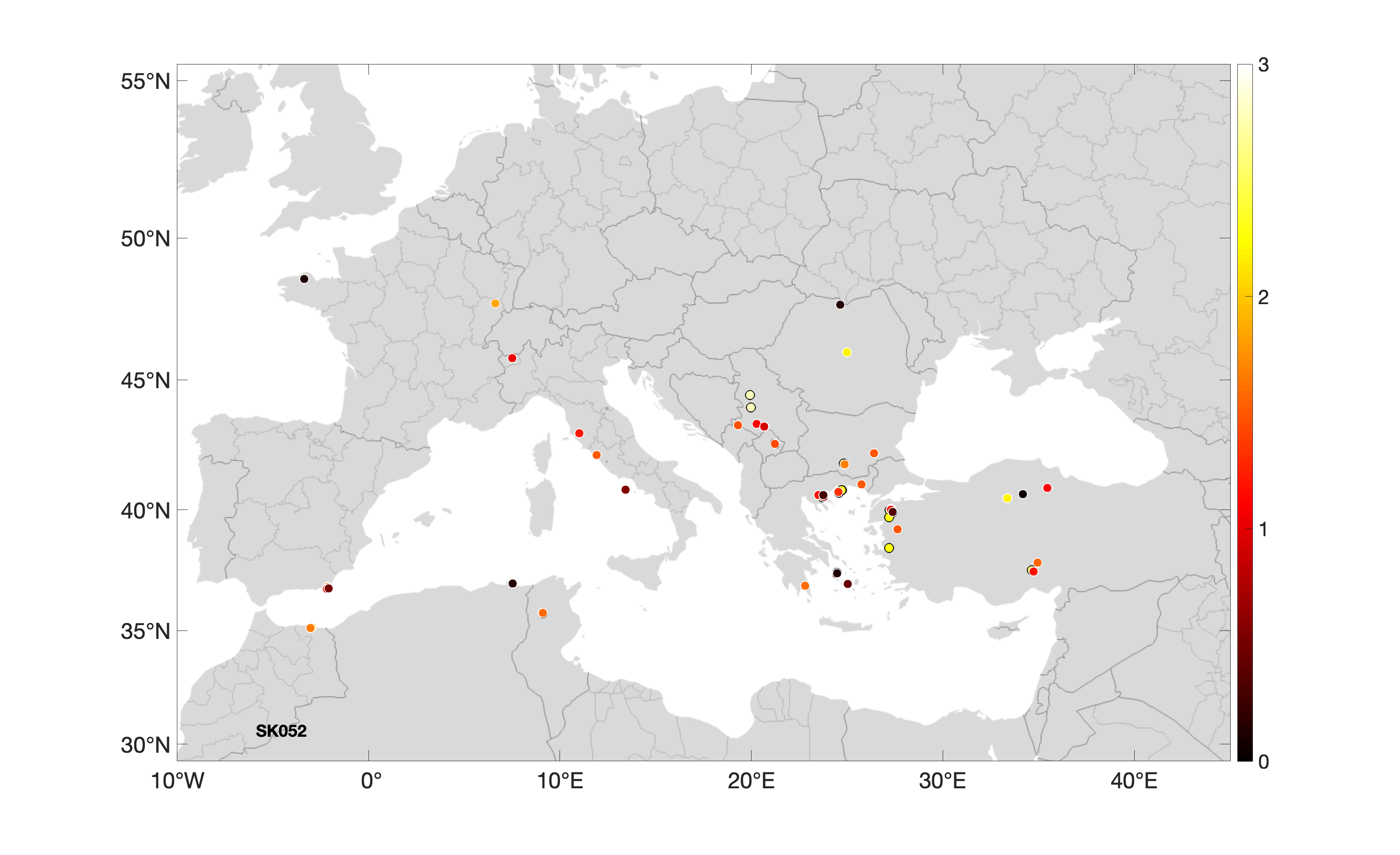

Supplement: Supplementary file 3 — Supplementary Material 3 [file 12520_2024_2106_MOESM3_ESM.zip › ESM3/png_hit maps/SK052_map_jittered.png]

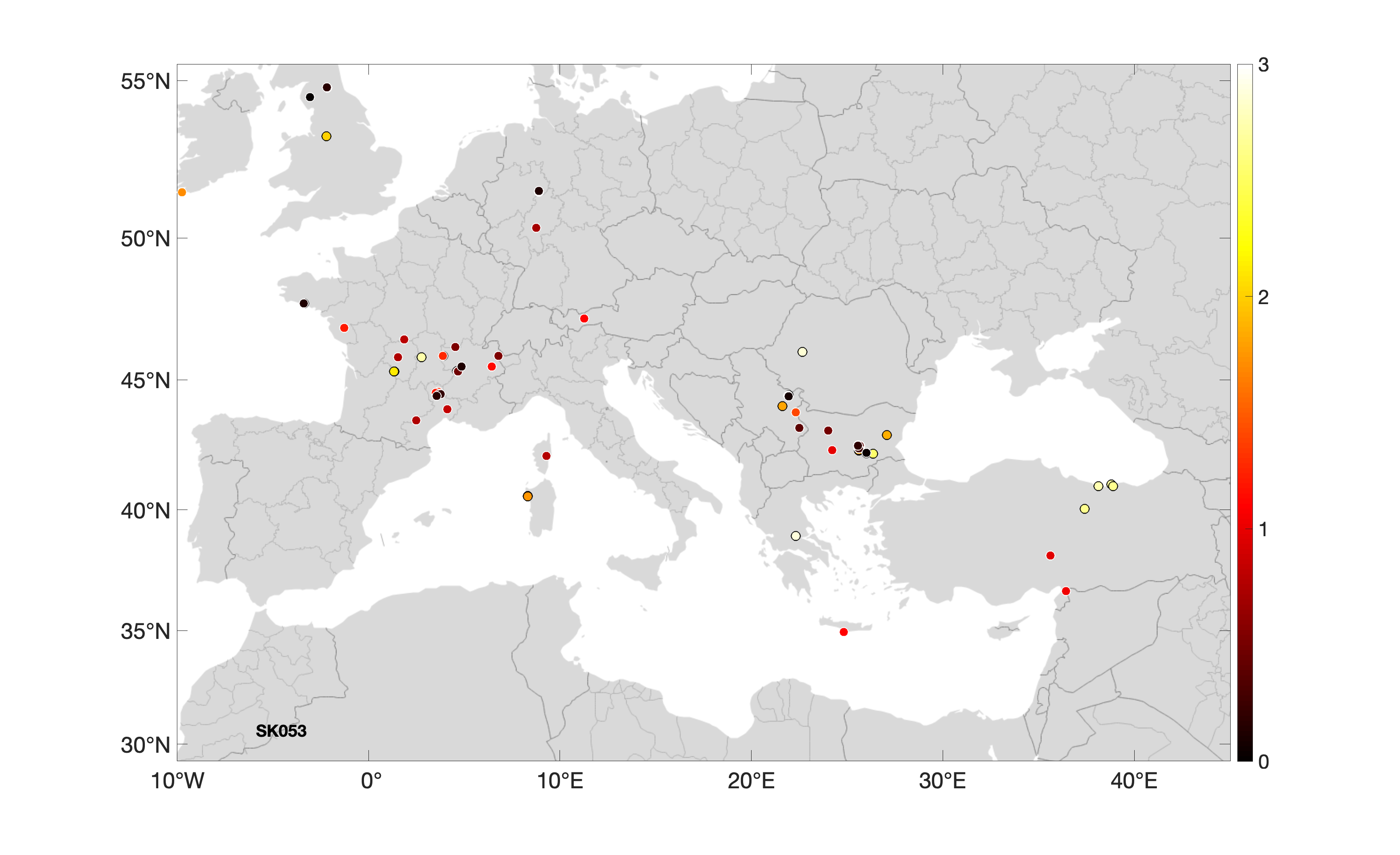

Supplement: Supplementary file 3 — Supplementary Material 3 [file 12520_2024_2106_MOESM3_ESM.zip › ESM3/png_hit maps/SK053_map_jittered.png]

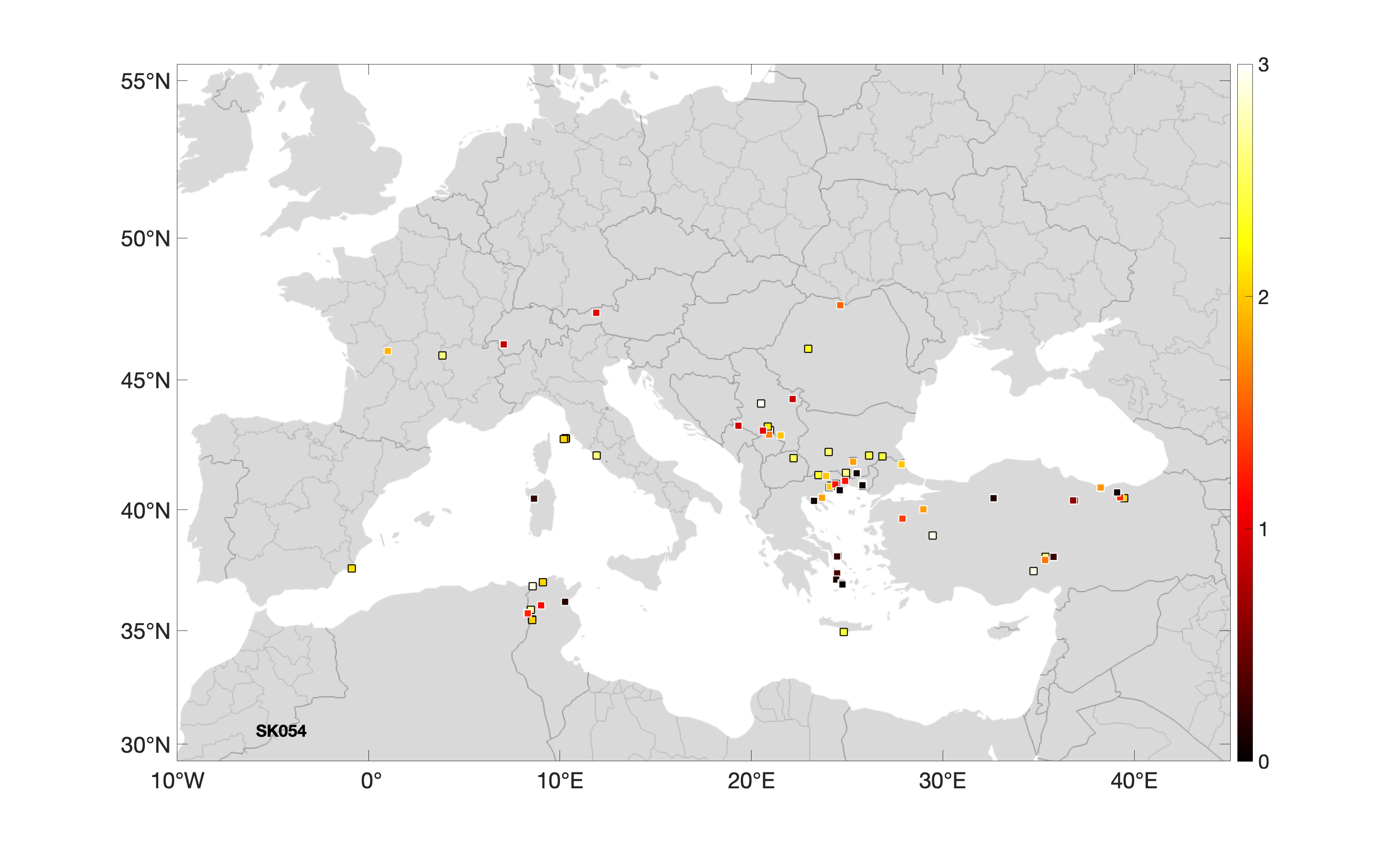

Supplement: Supplementary file 3 — Supplementary Material 3 [file 12520_2024_2106_MOESM3_ESM.zip › ESM3/png_hit maps/SK054_map_jittered.png]

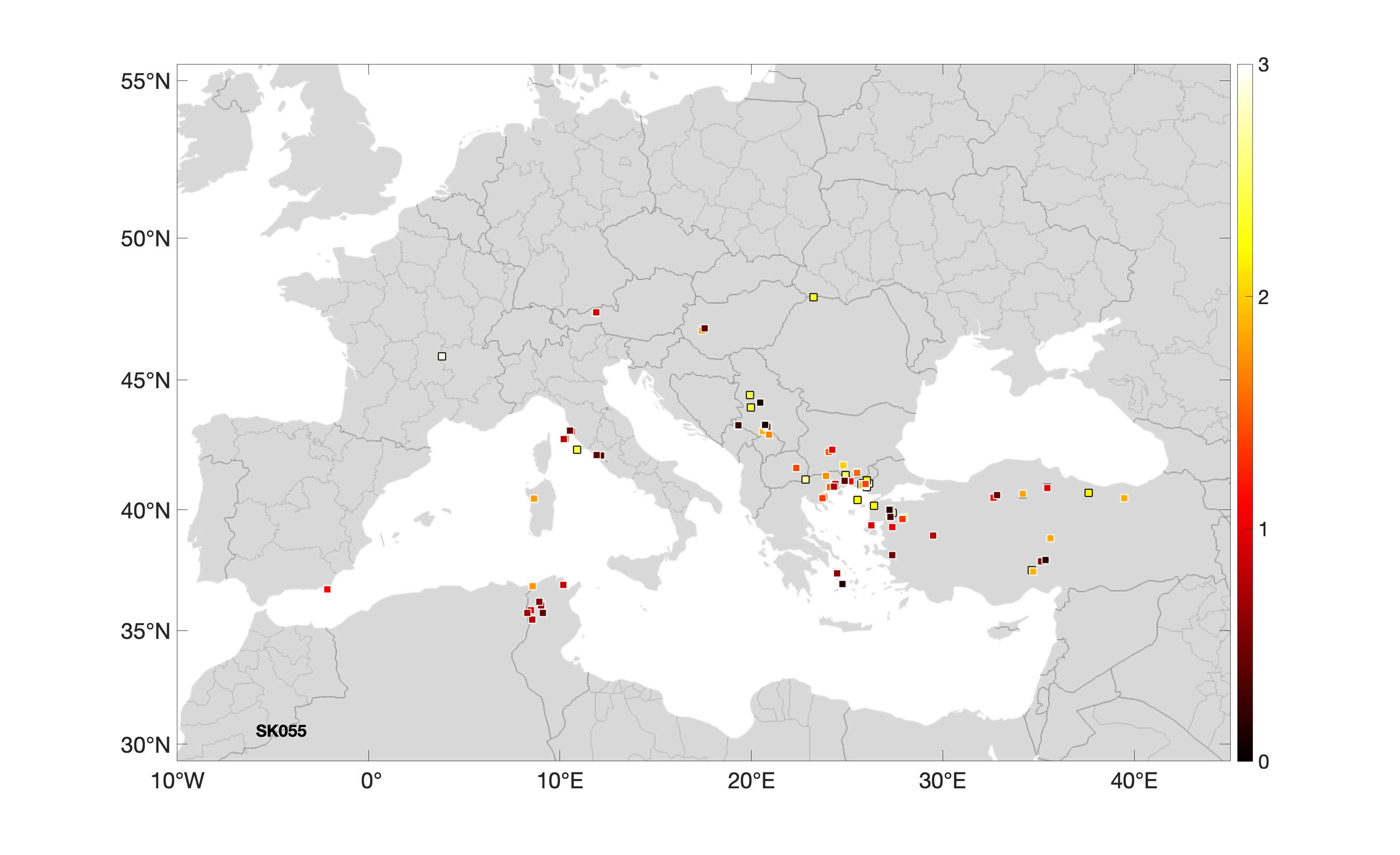

Supplement: Supplementary file 3 — Supplementary Material 3 [file 12520_2024_2106_MOESM3_ESM.zip › ESM3/png_hit maps/SK055_map_jittered.png]

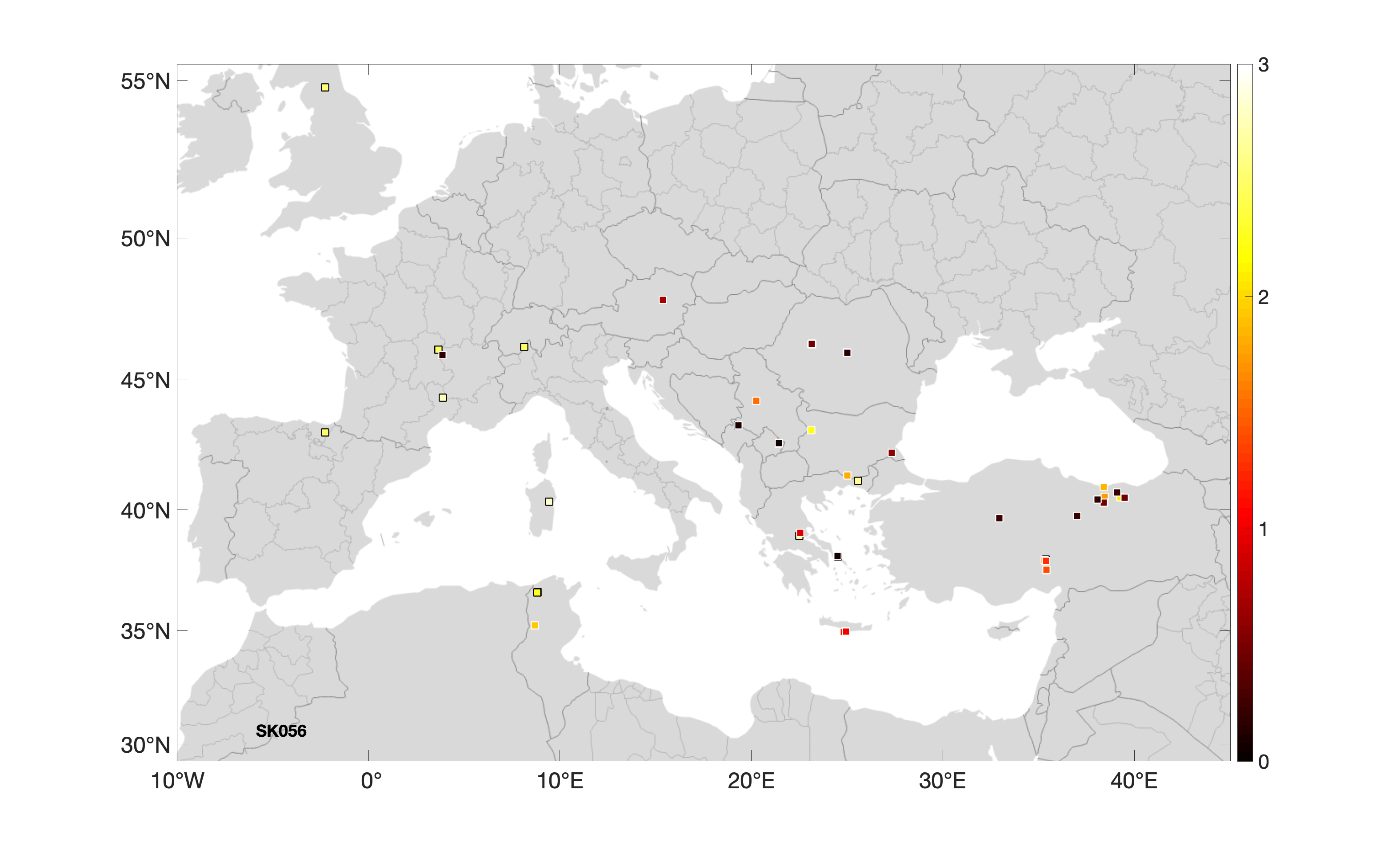

Supplement: Supplementary file 3 — Supplementary Material 3 [file 12520_2024_2106_MOESM3_ESM.zip › ESM3/png_hit maps/SK056_map_jittered.png]
